# Effects of Supplementation with Milk Proteins on Body Composition and Anthropometric Parameters: A Systematic Review and Dose–Response Meta-Analysis

**DOI:** 10.3390/nu17243877

**Published:** 2025-12-12

**Authors:** Shooka Mohammadi, Damoon Ashtary-Larky, Navid Alaghemand, Amneh F. Alnsour, Shokoufeh Shokouhifar, Aida Borzabadi, Milad Mehrbod, Darren G. Candow, Scott C. Forbes, Jose Antonio, Katsuhiko Suzuki, Omid Asbaghi

**Affiliations:** 1 Department of Social and Preventive Medicine, Faculty of Medicine, University of Malaya, Kuala Lumpur 50603, Malaysia; shooka.mohammadi@gmail.com; 2 Nutrition and Metabolic Diseases Research Center, Ahvaz Jundishapur University of Medical Sciences, Ahvaz 6135715794, Iran; n.alaghemand@gmail.com; 3 San Dimas Community Hospital, San Dimas, CA 91773, USA; aalnsour@phsi.us; 4 Department of Pediatrics, Faculty of Medicine, Ahvaz Jundishapur University of Medical Sciences, Ahvaz 6135715794, Iran; dr.shokouhifar2018@gmail.com; 5 Department of Internal Medicine, Faculty of Medicine, Alborz University of Medical Sciences, Karaj 3149969415, Iran; aidaborzabadi@gmail.com; 6 Department of Internal Medicine, Mercy San Juan Medical Center, Carmichael, CA 95608, USA; milad.mehrbod@commonspirit.org; 7 Faculty of Kinesiology and Health Studies, University of Regina, Regina, SK S4S 0A2, Canada; darren.candow@uregina.ca; 8 Department of Physical Education Studies, Faculty of Education, Brandon University, Brandon, MB R7A 6A9, Canada; forbess@brandonu.ca; 9 Department of Health and Human Performance, Nova Southeastern University, Davie, FL 32004, USA; jose.antonio@nova.edu; 10 Faculty of Sport Sciences, Waseda University, Tokorozawa 359-1192, Japan; 11 Cancer Research Center, Shahid Beheshti University of Medical Sciences, Tehran 1985717413, Iran; omid.asbaghi@gmail.com

**Keywords:** milk protein, anthropometric, whey protein, body composition, casein protein, protein supplementation

## Abstract

**Background/Objectives:** There is no consensus regarding the impacts of supplementation with milk proteins (MPs) on body composition (BC). This systematic review and dose–response meta-analysis of randomized controlled trials (RCTs) assessed the effects of MP, casein protein (CP), and whey protein (WP) supplementation on BC and anthropometric parameters. **Methods:** A comprehensive search was performed in several databases to identify eligible RCTs published until October 2025. Random-effects models were applied to estimate the pooled effects of MP supplementation on anthropometric parameters. **Results:** A total of 150 RCTs were included. MP supplementation substantially increased lean body mass (LBM) (weighted mean difference (WMD): 0.41 kg; 95% CI: 0.19, 0.62; *p* < 0.001) and fat-free mass (FFM) (WMD: 0.67 kg; 95% CI: 0.40, 0.94; *p* < 0.001). It also significantly reduced body fat percentage (BFP) (WMD: −0.66%; 95% CI: −1.03, −0.28; *p* = 0.001), fat mass (FM) (WMD: −0.66 kg; 95% CI: −0.91, −0.41; *p* < 0.001), and waist circumference (WC) (WMD: −0.69 cm; 95% CI: −1.16, −0.22; *p* = 0.004). No considerable effects were observed for muscle mass (MM), body mass index (BMI), and body weight (BW). Dose–response analysis revealed that MP dosage was associated with significant changes in BFP, LBM, and MM. **Conclusions:** MP supplementation was associated with favorable modifications in body composition, including increases in LBM and FFM, as well as reductions in FM, BFP, and WC. These findings provide coherent and consistent evidence supporting the potential role of MP supplementation in targeted body composition management.

## 1. Introduction

Supplementation with milk proteins (MPs) has been widely investigated for its potential effects on body composition (BC), particularly in individuals with specific nutritional needs or those engaged in high levels of physical activity [1,2]. Dairy-derived proteins enhance satiety, improve glycemic regulation, and support weight management [3,4]. Whey protein (WP) and milk protein concentrate (MPC) notably affect lean body mass (LBM) and body fat, positioning them as effective strategies for improving BC [1]. Incorporating milk products into the diet improves skeletal muscle mass (MM) and reduces body fat in young women with insufficient protein intake [5]. Among individuals participating in resistance training (RT), MPC supplementation has been associated with reductions in fat mass (FM) and body fat percentage (BFP), along with increases in LBM [6]. It has been indicated that MP supplementation, with or without RT, may improve MM and strength in older adults [7,8].

Cow’s milk provides essential macro- and micronutrients, along with high-quality proteins, making it an important component of a balanced diet [9,10]. Dairy proteins are primarily composed of whey and casein, which account for approximately 20% and 80% of the total amino acids (AAs), respectively [11]. These proteins differ markedly in their digestion and absorption kinetics [12]. WP is rapidly digested, in contrast to casein protein (CP), which is absorbed at a slower rate [13]. CP supplies all essential AAs except cysteine [14], whereas WP is particularly rich in branched-chain amino acids (BCAAs) (isoleucine, valine, and leucine) at higher concentrations than CP [15,16]. Leucine serves as a key regulator that stimulates muscle protein synthesis [17]. Conversely, CP contains higher amounts of non-essential AAs than WP [15]. Both WP and CP have received increasing attention from researchers and consumers because of their potential health benefits [18,19,20,21,22]. WP, one of the most commonly used supplements among athletes [23], provides BCAAs that promote muscle protein synthesis [24] and is safe for improving BC and reducing cardiovascular risk factors [14,20,21,25].

Several reviews and meta-analyses have examined the impacts of MP and WP supplementation, with or without RT, on BC [1,2,26,27,28,29,30]. However, the existing evidence is fragmented. Prior reviews have largely focused on either WP or CP in isolation, emphasized resistance-trained or athletic populations, or have not evaluated dose–response relationships. Furthermore, the effects of MP supplementation across diverse consumer groups on a broader range of anthropometric outcomes remain insufficiently characterized. These limitations have led to inconsistent or contradictory findings, preventing the development of clear, evidence-based recommendations for the use of MP supplementation to improve BC. Therefore, this systematic review and dose–response meta-analysis of randomized controlled trials (RCTs) aimed to comprehensively assess the effects of MP supplementation on BC and anthropometric parameters in adults and provide robust and clinically relevant evidence.

## 2. Methods

This systematic review and meta-analysis were implemented following the recommendations outlined in the Preferred Reporting Items for Systematic Reviews and Meta-Analyses (PRISMA) 2020 statement [31] and the Cochrane Handbook for Systematic Reviews of Interventions. In addition, the systematic review protocol was registered in the International Prospective Register of Systematic Reviews (PROSPERO) (No. CRD42025634923).

### 2.1. Search Strategy

Two investigators searched some databases (Scopus, PubMed/MEDLINE, and Web of Science) for potential RCTs published until October 2025. A grey literature search was performed using Google Scholar and trial registries to detect additional studies. The reference lists of relevant systematic reviews and included trials were also screened to find any further RCTs. When full texts were not accessible, the corresponding authors were contacted to request the necessary information and full texts.

The search strategy was structured around the PICOS framework (Population, Intervention, Comparator, Outcomes, and Study design) [32] to guide the identification of eligible studies. Search strategies were customized for each database. Both Medical Subject Headings (MeSH) and non-MeSH keywords were used. Boolean operators (OR, AND) were applied to combine search terms and enhance the overall sensitivity of the search. Body composition and anthropometric parameters were MM, LBM, FM, BFP, fat-free mass (FFM), body mass index (BMI), waist circumference (WC), and body weight (BW).

The search strategy included the following terms: (“milk protein” OR “milk” OR “milk protein supplementation” OR “milk protein supplement” OR “casein” OR “whey” OR “whey supplementation” OR “whey supplement” OR “casein supplementation” OR “casein supplement” OR “MPC” OR “milk protein concentrate” OR “whey protein hydrolysates” OR “WPH”) AND (“body weight” OR “body mass index” OR “BMI” OR “WC” OR “waist circumference” OR “BFP” OR “body fat percentage” OR “FFM” OR “fat-free mass” OR “FM” OR “fat mass” OR “LBM” OR “lean body mass” OR “muscle mass” OR “MM”) AND (“randomized controlled trial” OR “RCT” OR “clinical trial”). The search strategy in PubMed is provided in Table S1.

### 2.2. Selection Criteria

All citations retrieved for this meta-analysis were transferred into EndNote for reference management. Study selection was performed independently by two researchers, and any differences in assessment were addressed in consultation with a third investigator. Eligible RCTs evaluated the effects of supplementation with MP on BC and anthropometric measurements in adults and compared the intervention with a placebo or standard control. Both crossover and parallel RCTs were included. Studies were required to have an intervention duration of at least 2 weeks, enroll participants aged ≥ 18 years, and report at least one outcome of interest (FFM, BMI, WC, MM, LBM, FM, BFP, or BW) at both baseline and post-intervention. Early anabolic and atrophic responses in muscle protein metabolism can occur within days, and previous meta-analyses have documented measurable lean-mass changes within 14 days [33]. Therefore, a ≥2-week minimum intervention duration was selected to ensure inclusion of trials capable of producing early physiological adaptations while excluding very short exposure periods unlikely to yield meaningful changes. Trials were excluded if MP was provided as part of a multicomponent supplement in the intervention or control group. Additional exclusion criteria were the absence of a control or placebo arm, enrollment of pregnant women or participants < 18 years, the use of observational or other non-randomized designs, failure to meet the ≥2-week minimum intervention duration, or a lack of adequate baseline or post-intervention data for at least one outcome of interest.

### 2.3. Data Extraction

Data extraction was conducted independently by two investigators, and any discrepancies were settled through consultation with another researcher. The extracted information included study characteristics such as trial design, duration, setting, sample size, first author name, publication year, and MP dose. Participant demographics, including BMI, sex, and age, were also collected. The outcomes of interest (WC, FFM, BMI, FM, BW, LBM, MM, and BFP) were recorded at baseline and at the post-intervention time point.

### 2.4. Risk of Bias Assessment

The risk of bias in each included study was independently evaluated by two reviewers using the Cochrane Risk of Bias 2 (RoB 2) tool. Any differences in their assessments were addressed through consultation with a third researcher. The RoB 2 framework evaluated study quality through structured signaling questions across five key areas: how well participants were randomized, whether any departures from assigned interventions may have influenced outcomes, the extent and impact of missing outcome data, the appropriateness and consistency of outcome measurement, and whether the reported findings align with pre-specified analyses. Based on these evaluations, each domain was rated as “low risk,” “some concerns,” or “high risk” of bias [34].

### 2.5. Certainty Assessment

The certainty of evidence for each outcome was evaluated using the Grading of Recommendations Assessment, Development, and Evaluation (GRADE) approach. This framework evaluated five key areas (indirectness, RoB, imprecision, inconsistency, and potential publication bias). GRADE classified the certainty of evidence as high, moderate, very low, or low [35]. Two reviewers conducted the assessments independently, and any differences were resolved through discussion.

### 2.6. Statistical Analysis

All statistical analyses were conducted using STATA software (version 17). Outcomes were summarized as mean values with their corresponding standard deviations (SD), and effect sizes were expressed as mean differences. To compare changes from baseline to post-intervention between the MP and placebo groups, weighted mean differences (WMDs) with 95% confidence intervals (CIs) were calculated [36]. Pooled WMDs were estimated using a random-effects model [36]. Between-trial heterogeneity was assessed using the *I*^2^ statistic and Cochran’s Q test [36]. *I*^2^ values were classified as low (0–25%), moderate (26–50%), substantial (51–75%), or considerable (>75%) heterogeneity [37].

Subgroup analyses were implemented to detect possible factors contributing to heterogeneity, such as participant sex (both sexes, male, female), health status (unhealthy vs. healthy), protein type (MP, WP, CP), baseline BMI (overweight, obesity, and normal), age (>60 vs. ≤60 years), trial duration (>8 vs. ≤8 weeks), and MP dosage (>30 vs. ≤30 g/day). Sensitivity analyses were applied to evaluate the effect of each trial on overall results.

Publication bias was evaluated by inspecting funnel plot symmetry, as well as Begg’s [38] and Egger’s [39] tests. Statistical significance was *p* < 0.05. Dose–response relationships were examined using the fractional polynomial method [40]. It was applied to explore potential non-linear associations between MP dosage (g/day) or intervention duration (weeks) and changes in the outcomes. Meta-regression analyses were carried out to examine linear dose–response associations between MP dosage or trial duration and the corresponding changes in outcomes [41].

## 3. Results

### 3.1. Study Selection

**Figure 1:**
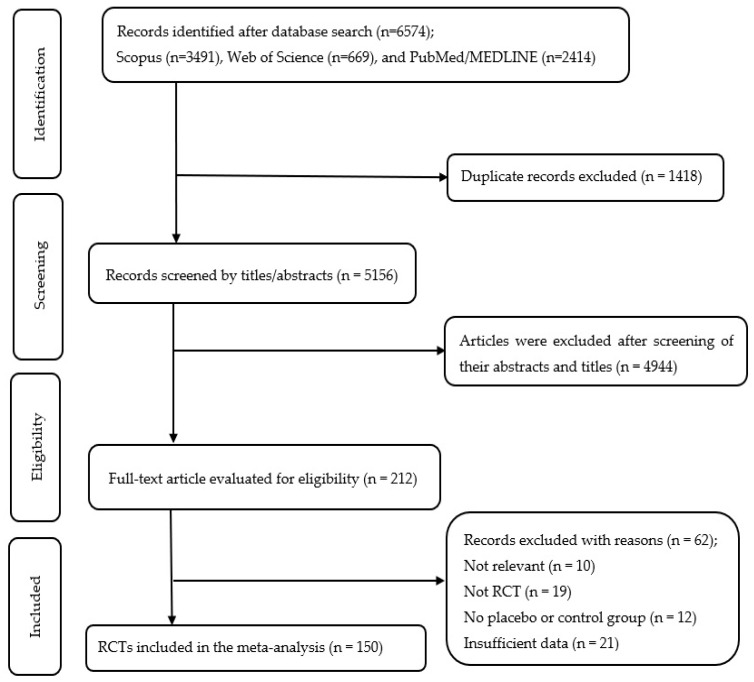
Flow diagram of study selection.

A comprehensive search among several databases retrieved 6574 records, and 1418 duplicate entries were subsequently excluded. Screening of abstracts and titles for the remaining 5156 records led to the exclusion of 4944. The full-text assessment of 212 articles resulted in the inclusion of 150 studies in the current meta-analysis. Figure 1 displays the flow diagram outlining the stages of screening and selecting studies.

### 3.2. Study Characteristics

This systematic review and dose–response meta-analysis included 150 RCTs [12,42,43,44,45,46,47,48,49,50,51,52,53,54,55,56,57,58,59,60,61,62,63,64,65,66,67,68,69,70,71,72,73,74,75,76,77,78,79,80,81,82,83,84,85,86,87,88,89,90,91,92,93,94,95,96,97,98,99,100,101,102,103,104,105,106,107,108,109,110,111,112,113,114,115,116,117,118,119,120,121,122,123,124,125,126,127,128,129,130,131,132,133,134,135,136,137,138,139,140,141,142,143,144,145,146,147,148,149,150,151,152,153,154,155,156,157,158,159,160,161,162,163,164,165,166,167,168,169,170,171,172,173,174,175,176,177,178,179,180,181,182,183,184,185,186,187,188,189,190]. Their characteristics are summarized in Table 1. Across 150 studies, 7998 participants were enrolled (MP group: n = 3979; control group: n = 4019), with sample sizes ranging from 10 to 208. The mean age of participants ranged from 18 to 86 years, with a mean BMI ranging from 18.5 to 46.5 kg/m^2^. In addition, 73 trials recruited mixed-sex samples [42,43,44,45,46,47,48,49,50,51,52,53,54,55,56,57,58,59,60,61,62,63,64,65,66,67,68,69,70,71,72,73,74,75,76,77,78,79,80,81,82,83,84,85,86,87,88,89,90,91,92,93,94,95,96,97,98,99,100,101,102,176,177,178,180,181,183,185,186,187,188,189,190], 28 were performed exclusively among female participants [12,103,104,105,106,107,108,109,110,111,112,113,114,115,116,117,118,119,120,121,122,123,124,125,126,127,174,179], and 49 included only men [128,129,130,131,132,133,134,135,136,137,138,139,140,141,142,143,144,145,146,147,148,149,150,151,152,153,154,155,156,157,158,159,160,161,162,163,164,165,166,167,168,169,170,171,172,173,175,182,184].

The trials were conducted across diverse participants, including dialysis patients [57,61,81,82,86]; older adults [52,66,68,71,75,77,83,84,85,87,89,90,93,94,95,96,98,99,103,108,110,111,114,119,121,124,159,164,184,185,186,187,188,189,190] with sarcopenic obesity [106,169]; individuals with overweight or obesity [42,43,48,49,50,53,54,55,56,59,67,70,78,80,105,107,112,115,116,117,128,129,130,137,174], abdominal obesity [76,91] hypertension (HTN) and pre-HTN [44,58,132], or increased visceral fat [45]; individuals who underwent laparoscopic sleeve gastrectomy [180]; patients with type 2 diabetes mellitus (T2DM) [64,101,109,126,158,175], human immunodeficiency virus (HIV) infection [69,104], metabolic syndrome (MetS) [47], cystic fibrosis (CF) [51], amyotrophic lateral sclerosis (ALS) [60], cancer [63,88], chronic obstructive pulmonary disease (COPD) [65,72,100], sarcopenia [123,139], chronic liver disease [97], or hyperlipidemia [145]; pre-menopausal women [179]; postmenopausal women [118,154] who underwent bariatric surgery [125]; patients who underwent one anastomosis gastric bypass (OAGB) [181]; patients with chronic heart disease (CHD) [102]; and women with polycystic ovary syndrome (PCOS) [127]. Trials were also performed among healthy individuals [62,79,92,113,133,134,138,140,141,143,144,146,150,151,157,161,163,165,166,167,168,170,171,177], nursing home residents [73], midlife adults [46], sedentary individuals [183], basketball players [120,135], futsal players [136], trained men [74,131,147,148,153,155,156], male bodybuilders [142], physically active men [149,182], recreationally active men [172], well-trained endurance athletes [173], master triathletes [152], untrained individuals [176,178], collegiate female athletes [12], collegiate female dancers [122], and army soldiers [160,162].

The articles were published between 2000 and 2025. The RCTs were carried out in multiple countries, including Finland [73,138,150], the Netherlands [42,83,89,172,182,190], Australia [43,67,103,119,142], Japan [45,72,93,94,121,123,132], Iran [61,65,109,115,117,126,128,129,130,135,144,174,181], France [66,149], Tunisia [173], Brazil [60,97,99,101,102,106,110,111,116,118,124,125,175], Germany [47,169,177,178], Denmark [49,54,56,91,98], Canada [51,76,78,80,113,133,139,141,161,164,186], and the United States of America (USA) [12,44,46,48,50,52,53,55,57,59,62,69,70,74,75,77,79,85,92,95,104,105,107,112,114,120,122,127,131,134,140,143,145,147,148,151,154,155,156,157,160,162,165,170,176,183,188]. Trials were also conducted in China [58,90,96,100,108], Thailand [63], Italy [64,88], Portugal [136], Sweden [137,146], the Czech Republic [68], Norway [71,84], the United Kingdom (UK) [152,153,184,187,189], Israel [81,82], New Zealand [158,159,179], Malaysia [86], South Korea [168,171], Spain [87], Turkey [180], Iceland [185], Serbia [163], Chile [166], and Saudi Arabia [167]. Trial durations varied from 2 to 96 weeks, and the daily doses of CP, MP, and WP ranged between 3.14 and 137 g.

### 3.3. Effect of Supplementation with MP on BW

**Figure 2:**
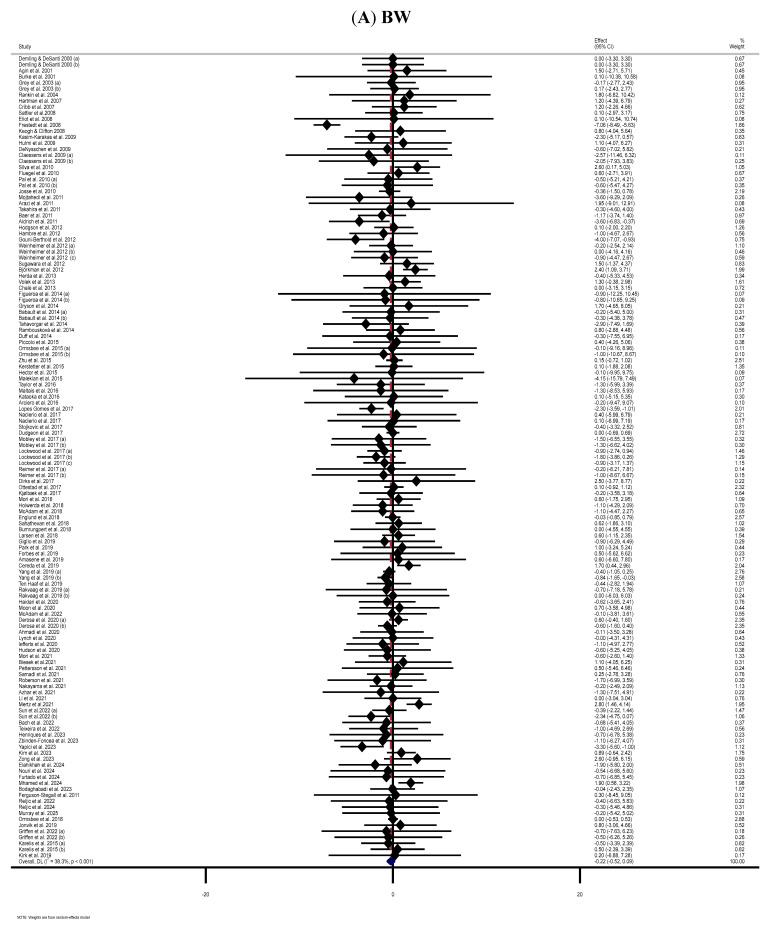

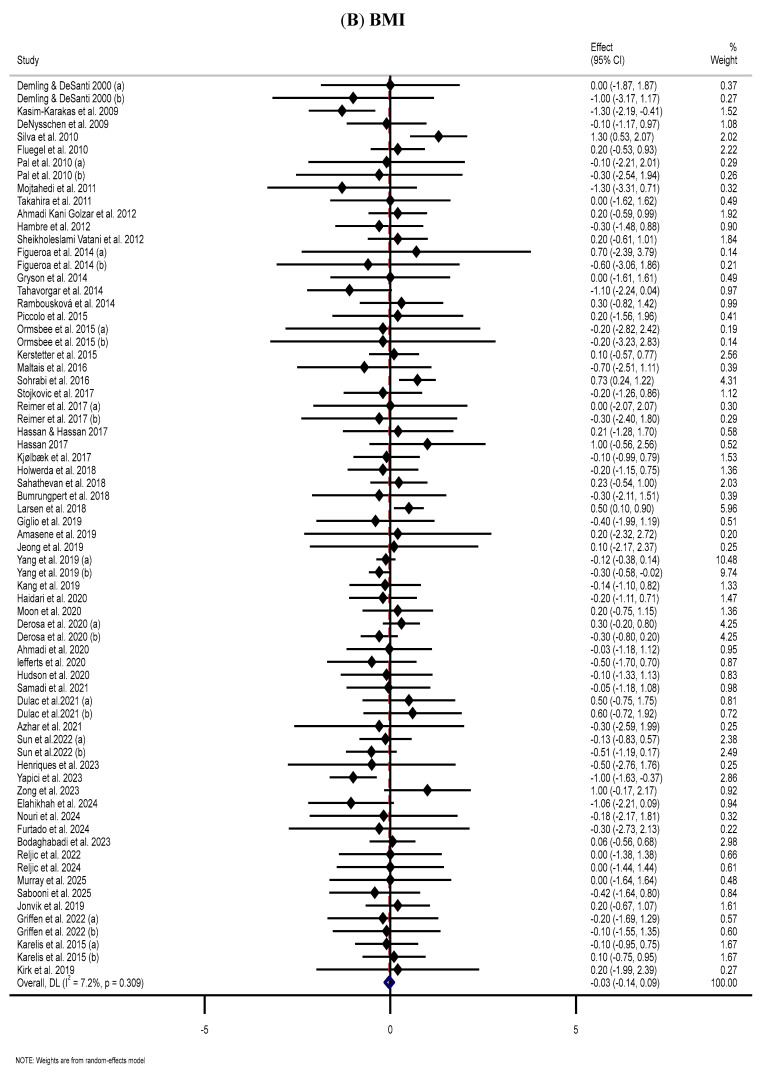

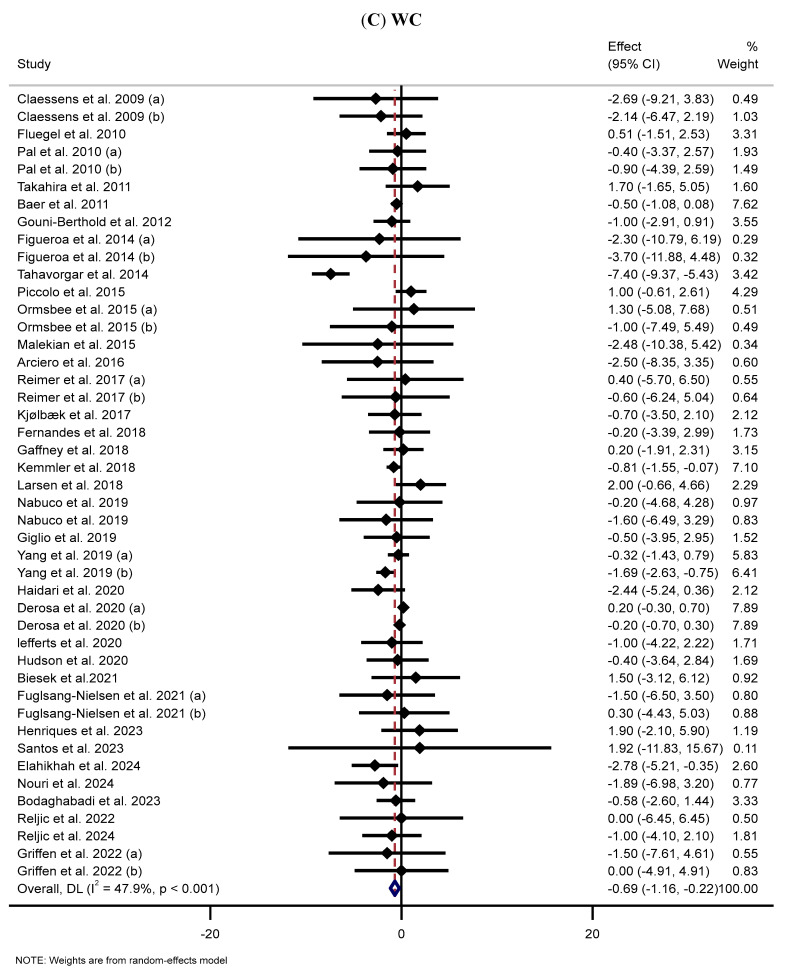

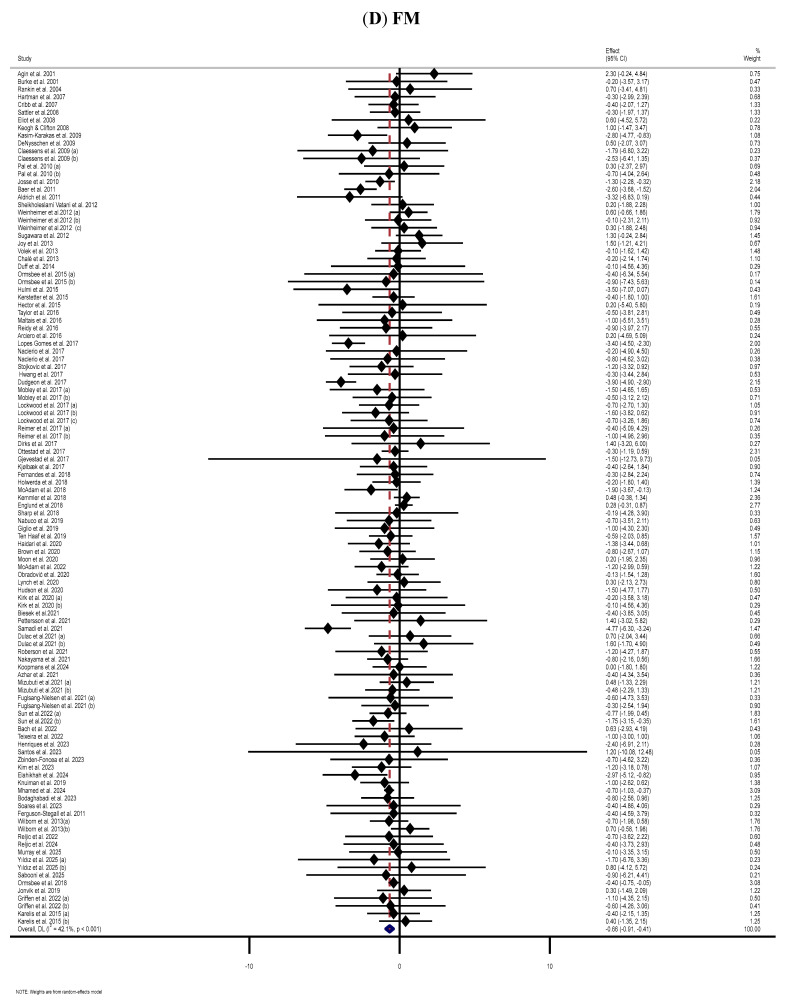

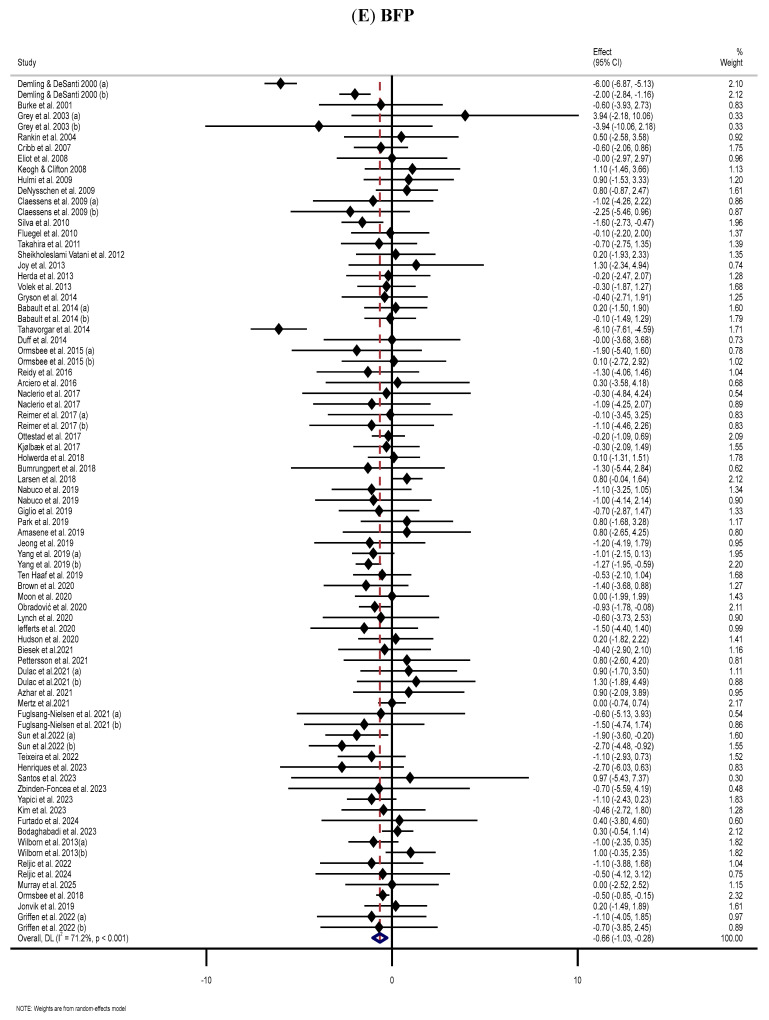

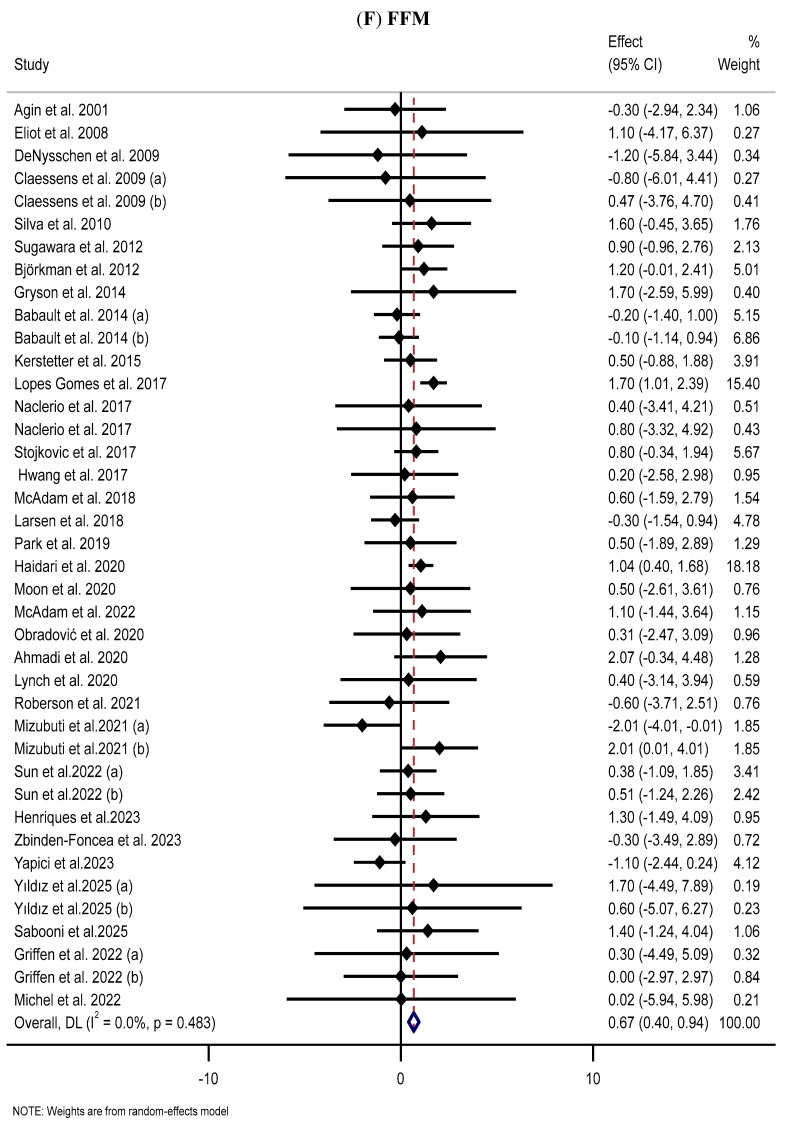

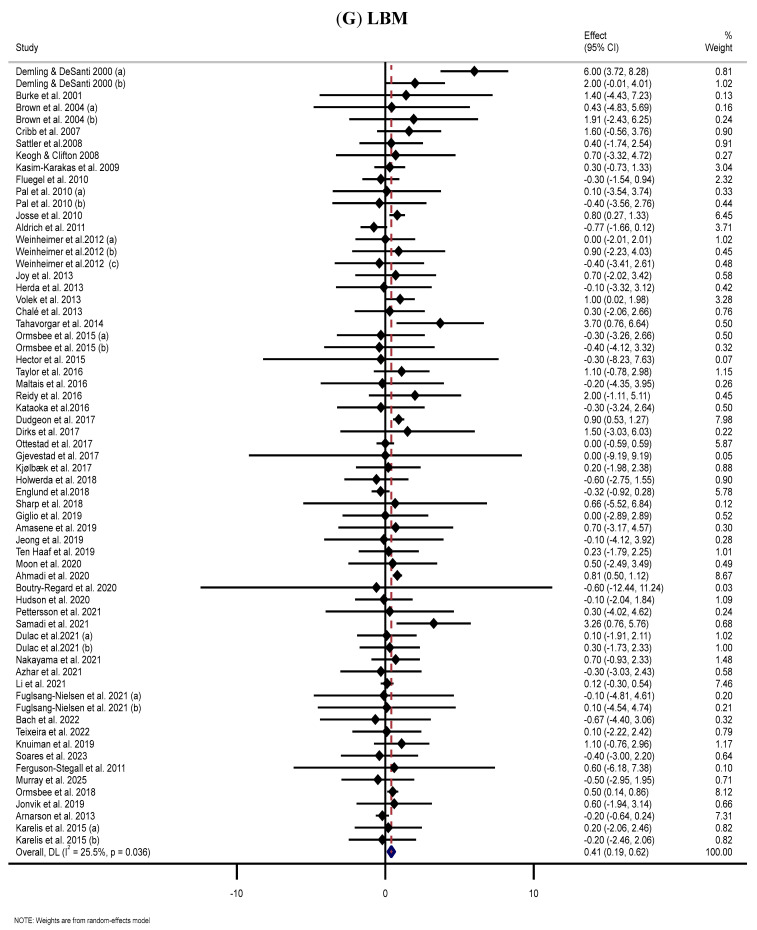

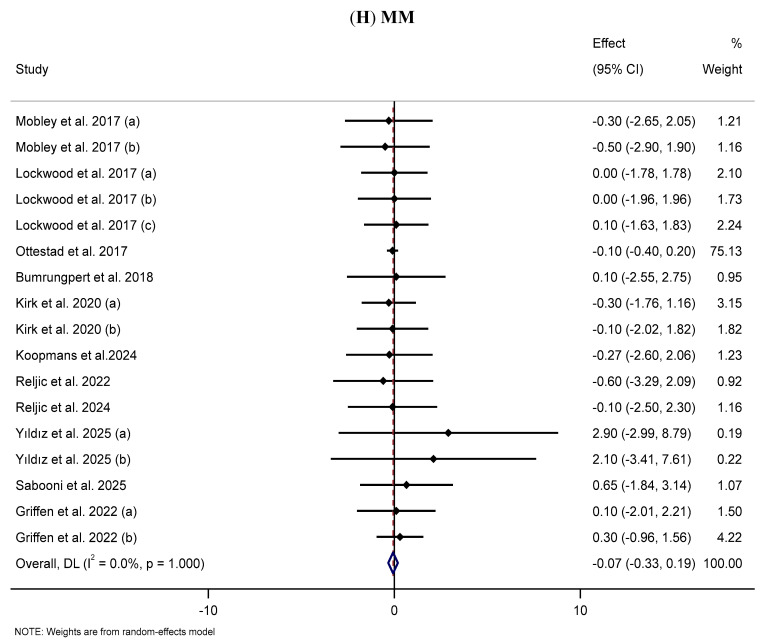
The forest plots illustrate the WMDs and 95% CIs regarding the impact of MP supplementation on (**A**) BW (Kg), (**B**) BMI (kg/m^2^), (**C**) WC (cm), (**D**) FM (kg), (**E**) BFP (%), (**F**) FFM (Kg), (**G**) LBM (kg), and (**H**) MM (kg) [12,42,43,44,45,46,47,48,49,50,51,52,53,54,55,56,57,58,59,60,61,62,63,64,65,66,67,68,69,70,71,72,73,74,75,76,77,78,79,80,81,82,83,84,85,86,87,88,89,90,91,92,93,94,95,96,97,98,99,100,101,102,103,104,105,106,107,108,109,110,111,112,113,114,115,116,117,118,119,120,121,122,123,124,125,126,127,128,129,130,131,132,133,134,135,136,137,138,139,140,141,142,143,144,145,146,147,148,149,150,151,152,153,154,155,156,157,158,159,160,161,162,163,164,165,166,167,168,169,170,171,172,173,174,175,176,177,178,179,180,181,182,183,184,185,186,187,188,189,190].

The meta-analysis of 114 RCTs [42,43,44,45,46,47,48,49,50,51,52,53,55,56,58,59,60,63,64,65,66,67,68,69,70,71,72,73,74,75,76,77,78,79,80,83,85,86,87,88,89,91,92,94,95,96,98,99,100,101,103,104,105,107,108,112,113,114,115,116,117,118,119,120,121,123,124,125,126,127,130,131,132,133,134,135,136,137,138,139,141,142,143,144,145,146,148,149,152,153,154,156,157,159,160,161,162,165,166,167,168,170,171,173,174,176,177,178,179,182,183,184,186,187] found no statistically significant impact of MP consumption on BW in the MP-treated group compared to the control group (WMD: −0.22 kg, 95% CI: −0.52, 0.09; *p* = 0.160). Moderate heterogeneity was observed among the included RCTs (*I*^2^ = 38.3%, *p* < 0.001) (Figure 2A). Subgroup analyses showed significant reductions in BW with MP supplementation among women, participants aged ≤60 years, and individuals with obesity. However, it significantly increased BW in participants older than 60 years (Table 2).

### 3.4. Effect of Supplementation with MP on BMI

The meta-analysis of 59 trials [43,44,45,49,50,52,53,56,57,58,60,61,63,64,65,66,68,77,80,81,82,86,87,90,95,100,101,105,107,108,112,114,115,116,117,125,126,127,128,129,130,131,135,139,145,146,154,159,164,167,174,177,178,179,181,182,184,186,187] revealed no statistically substantial differences in BMI between the MP and placebo groups (WMD: −0.03 kg/m^2^, 95% CI: −0.14, 0.09; *p* = 0.626) (Figure 2B). Subgroup analyses indicated substantial reductions in BMI among female participants and those who consumed MP supplements (Table 2).

### 3.5. Effect of Supplementation with MP on WC

The meta-analysis of 36 studies [42,43,44,45,47,48,49,52,53,54,56,58,64,70,79,80,102,105,106,107,110,111,112,115,116,117,124,125,126,130,158,169,174,177,178,184] revealed that MP supplementation significantly reduced WC in the MP group compared to the placebo group (WMD: −0.69 cm, 95% CI: −1.16, −0.22; *p* = 0.004) (Figure 2C). Moderate heterogeneity was identified among the included studies (*I*^2^ = 47.9%, *p* < 0.001). Subgroup analyses displayed that long-term supplementation (>8 weeks) with high doses (>30 g/day) of WP markedly decreased WC in healthy participants and individuals with obesity (regardless of sex or age) (Table 2).

### 3.6. Effect of Supplementation with MP on FM

The meta-analysis, which included 93 trials [12,42,43,46,48,53,54,55,56,62,67,69,70,71,72,74,75,76,77,78,80,83,84,85,89,92,94,95,97,99,102,104,106,107,108,111,113,115,116,117,118,120,122,124,125,127,129,131,133,134,135,136,137,138,139,141,142,143,145,147,151,152,153,154,155,156,157,159,160,162,163,164,165,166,168,169,170,172,173,174,175,176,177,178,179,180,181,182,183,184,186,189,190] demonstrated that supplementation with MP substantially decreased FM in the MP group compared with the placebo group (WMD: −0.66 kg, 95% CI: −0.91, −0.41; *p* < 0.001) (Figure 2D). Moderate heterogeneity was detected among the trials (*I*^2^ = 42.1%, *p* < 0.001). Subgroup analyses further revealed that supplementation with MP or WP significantly decreased FM, particularly in healthy participants aged ≤ 60 years (irrespective of dose, duration, sex, or BMI) (Table 2).

### 3.7. Effect of Supplementation with MP on BFP

The meta-analysis of 68 RCTs [12,42,44,45,48,49,50,51,52,53,54,56,57,58,60,63,66,67,71,74,76,80,87,89,92,95,98,101,102,106,107,108,110,116,122,124,125,129,130,131,133,134,136,137,142,143,145,147,148,149,150,151,152,153,159,163,164,166,167,168,171,174,177,178,179,182,183,184] displayed substantial reductions in BFP following MP supplementation compared to the placebo group (WMD: −0.66%, 95% CI: −1.03, −0.28; *p* = 0.001) (Figure 2E). The analysis also revealed a very high level of heterogeneity among the included RCTs (*I*^2^ = 71.2%, *p* < 0.001). Subgroup analyses indicated that BFP significantly reduced during supplementation with WP or MP among participants aged ≤ 60 years and those with normal BMI (independent of dose, duration, sex, and health status) (Table 2).

### 3.8. Effect of Supplementation with MP on FFM

The effect of MP supplementation on FFM was assessed through the analysis of 34 RCTs [42,49,60,65,66,72,73,77,92,97,104,108,117,118,125,131,143,145,149,152,153,154,155,160,162,163,165,166,167,171,180,181,184,188]. The meta-analysis indicated that MP supplementation substantially increased FFM in the MP group compared with that in the placebo group (WMD: 0.67 kg, 95% CI: 0.40, 0.94; *p* < 0.001) (Figure 2F). Subgroup analyses further revealed that long-term supplementation with low WP doses significantly increased FFM among healthy participants and those with obesity (regardless of age or sex) (Table 2).

### 3.9. Effect of Supplementation with MP on LBM

The meta-analysis of 56 RCTs [43,44,46,50,53,54,55,56,57,62,65,67,69,71,74,75,78,83,84,85,87,89,93,94,95,96,99,107,113,116,120,127,130,131,132,133,135,136,137,139,140,142,147,148,151,156,159,164,172,175,176,179,182,183,185,186] revealed that MP supplementation significantly increased LBM in the MP group compared with the placebo group (WMD: 0.41 kg, 95% CI: 0.19, 0.62; *p* < 0.001) (Figure 2G). The analysis also revealed low heterogeneity among the included RCTs (*I*^2^ = 25.5%, *p* = 0.036). Subgroup analyses further indicated that LBM significantly increased after supplementation with WP or MP among participants with normal BMI (irrespective of dose, duration, sex, age, or health status) (Table 2).

### 3.10. Effect of Supplementation with MP on MM

The meta-analysis of 11 RCTs [63,71,157,170,177,178,180,181,184,189,190] did not demonstrate statistically significant impacts of MP supplementation on MM in the MP group compared with the placebo group (WMD: −0.07 kg, 95% CI: −0.33, 0.19; *p* = 0.588) (Figure 2H). Subgroup analyses also did not reveal any significant effects of supplementation with MP on MM (Table 2).

### 3.11. Publication Bias

Visual inspection of the funnel plots displayed asymmetry for all outcomes (Figure S1). However, Egger’s and Begg’s tests did not detect any evidence of publication bias for BMI, WC, FFM, BW, FM, LBM, BFP, and MM.

### 3.12. Risk of Bias Evaluation

The overall RoB of 150 included RCTs is summarized in Table S2. Among these studies, 99 RCTs were rated low RoB, while 51 were rated high RoB.

### 3.13. GRADE

Table S3 shows the certainty of evidence for the outcomes evaluated after MP supplementation. The evidence for BW, FM, FFM, BMI, WC, MM, and LBM was rated as high certainty, whereas the evidence for BFP was rated as moderate certainty.

### 3.14. Linear and Non-Linear Dose–Response Relations

Dose–response analyses revealed significant linear (−4.48, *p* = 0.011; Figure S4E) and non-linear (−0.04, *p* < 0.001; Figure S2E) associations between MP dose and changes in BFP. A significant linear relationship was also detected between MP dose and changes in LBM (5.66, *p* = 0.030; Figure S4G). In addition, a substantial non-linear association was identified between MP supplementation dose and change in MM (22.97, *p* = 0.003; Figure S2H).

### 3.15. Sensitivity Analysis

The leave-one-out sensitivity analysis revealed no changes in any of the evaluated outcomes.

## 4. Discussion

This systematic review and dose–response meta-analysis included 150 RCTs. It revealed that MP supplementation may beneficially influence specific BC and anthropometric parameters, as evidenced by increases in LBM and FFM and reductions in FM, BFP, and WC. However, it had no substantial effects on BW, MM, and BMI.

Subgroup analyses revealed that MP substantially reduced BW in women, participants aged ≤60 years, and individuals with obesity. However, it significantly increased BW in participants aged 60 years or older. In addition, significant reductions in BMI were observed among female participants. Long-term supplementation (>8 weeks) with high WP doses (>30 g/day) markedly decreased WC in healthy participants and those with obesity (regardless of sex or age). Supplementation with MP or WP significantly reduced FM in healthy participants aged ≤ 60 years (independent of dose, duration, sex, and BMI). Furthermore, BFP significantly declined during supplementation with WP or MP among participants aged ≤ 60 years and those with normal BMI (irrespective of dose, duration, sex, or health status). Long-term supplementation with low WP doses significantly increased FFM among healthy participants and those with obesity (independent of age or sex). Moreover, LBM significantly increased after supplementation with WP or MP among participants with normal BMI (independent of dose, duration, sex, age, and health status).

Dose–response analyses demonstrated significant linear and non-linear associations between MP dosage and changes in BFP. A substantial linear relationship was also observed between MP dose and changes in LBM, whereas a significant non-linear association was found between MP dose and changes in MM.

A meta-analysis of 35 RCTs demonstrated that WP supplementation improved several BC indicators, including FM, BMI, LBM, and WC [27]. The beneficial effects of WP on BC appeared to be most pronounced when combined with RT and an overall calorie restriction [27]. Another meta-analysis of 10 trials reported that concurrent MP supplementation and RT yielded favorable effects on FFM in older adults, although no significant changes were observed in FM or BW [28]. Moreover, a meta-analysis of nine studies indicated that WP supplementation may increase BW and total FM in individuals with obesity or overweight [1]. These divergent findings likely reflect differences in participant characteristics, baseline adiposity, energy intake, and concurrent RT across trials. A meta-analysis of 17 RCTs suggested that MP is more effective than WP in improving RT-induced LBM or FFM gains in older adults [26]. A recent meta-analysis reported that WP supplementation did not significantly improve anthropometric indicators, including FM, BFP, LBM, or WC, in older adults [30]. It has been revealed that WP is more effective than CP in stimulating protein synthesis in older adults [191]. In addition, milk proteins, particularly WP, may play a critical role in mitigating sarcopenia, a condition characterized by a progressive decline in MM [192,193,194].

### 4.1. Possible Underlying Mechanisms

The impact of MP on BC appears to be mediated through multiple physiological pathways involving satiety regulation, energy metabolism, and hormonal responses [3,195]. WP and CP exert distinct metabolic effects that influence weight management and BC [195]. Dairy proteins have been shown to enhance satiety more effectively than carbohydrates or fats, thereby reducing overall energy intake [3]. WP is primarily associated with short-term satiety, whereas CP contributes to prolonged feelings of fullness [195]. Additionally, dairy proteins may modulate energy expenditure and lipid metabolism via calcium- and vitamin D-dependent mechanisms that regulate lipolysis and fatty acid oxidation [196]. MP also improves postprandial glycemic control by attenuating blood glucose responses when co-ingested with carbohydrates [3], an effect linked to enhanced insulin sensitivity and more favorable long-term regulation of BW and BC [197,198].

The rapid digestion and absorption of WP lead to elevated circulating AAs [199]. This stimulates muscle protein synthesis and modestly inhibits muscle protein degradation after RT [200]. Therefore, the influence of WP on BC is closely associated with metabolic regulation and MM preservation [1]. WP also stimulates the release of appetite-regulating hormones, including dipeptidyl peptidase 4 (DPP-4), cholecystokinin (CCK), and glucagon-like peptide-1 (GLP-1) [201], contributing to appetite regulation [195]. Owing to its high biological value and rich BCAA profile, WP effectively supports muscle protein synthesis, which is a key determinant of BC maintenance during weight loss [125]. Furthermore, WP may promote the browning of white adipose tissue (WAT) and activate brown adipose tissue (BAT), thereby increasing energy expenditure and facilitating fat loss [202]. It has been suggested that uncoupling proteins and reduced lipogenesis may act as mechanisms contributing to improved weight management [202]. WP also enhances fat oxidation while preserving LBM, providing additional benefits for BC optimization [4,203].

In contrast, CP undergoes slower digestion, leading to the gradual release of AAs and prolonged satiety [195]. This sustained absorption may help maintain energy levels and reduce hunger, thereby supporting effective weight management [195]. CP intake has also been associated with the modulation of gastrointestinal hormones involved in appetite regulation, although evidence regarding its superiority over other protein sources is inconclusive [195]. Moreover, CP may influence metabolic hormones, potentially improving glucose metabolism and attenuating fat accumulation [204]. Overall, WP, CP, and MP exhibited distinct but complementary effects on BC, and their outcomes may vary according to individual metabolic profiles, physiological status, and dietary context.

### 4.2. Strengths and Limitations

This systematic review is the first dose–response meta-analysis that thoroughly assessed the effect of MP supplementation on BC. It included a large number of RCTs (n = 150) with sufficient sample sizes to identify statistically significant relationships between variables. The systematic literature search was unrestricted by publication date or language, reducing potential selection bias. Including recent studies from various regions improves the external validity and applicability of the results. The included RCTs enrolled adults with diverse health conditions, which enhances the generalizability of the findings and captures a wide range of potential responses across different populations. Additionally, the majority of studies demonstrated low RoB, and the GRADE assessment was high for all variables except BFP, which was rated as moderate.

However, this study had several limitations. Considerable heterogeneity was observed across the trials in terms of characteristics of participants, intervention duration, and supplement dosage. Further sources of heterogeneity included the use of different body composition assessment methods (e.g., dual-energy x-ray absorptiometry (DXA), bioelectrical impedance analysis (BIA), or skinfolds). Only short- to moderate-term trials were available, limiting the ability to assess long-term effects of WP, CP, or MP supplementation. Differences between the non-intervention and placebo groups also contributed to variability in outcomes. Additionally, variations in macronutrient composition, particularly total protein intake, between the intervention and control groups could have influenced BC outcomes independent of supplementation with MPs. Energy intake, a major determinant of BC, also differed among the studies and may have confounded their results. Moreover, most included trials focused on WP supplementation, whereas fewer studies investigated whole MP or CP supplementation. Therefore, additional RCTs are required to clarify the distinct and combined effects of CP and MP on BC and related anthropometric parameters. However, this meta-analysis provides a comprehensive and valuable insight for future studies.

## 5. Conclusions

This dose–response meta-analysis revealed that MP supplementation improved LBM, FFM, FM, BFP, and WC, supporting its potential as a feasible dietary approach to enhance BC. However, MP supplementation had no significant effect on BW, BMI, or MM. These findings should be interpreted cautiously due to heterogeneity across trials and the presence of several studies with high RoB. Well-designed, large-scale RCTs with longer follow-up periods are required to confirm these findings and determine the specific contributions of whole milk or CP supplementation to BC outcomes.

## Figures and Tables

**Table 1:** Characteristics of included RCTs in the meta-analysis.

Reference	Country	Study Design	Participants	Sex	Sample Size		Trial Duration (Weeks)	Mean Age		Mean BMI		Intervention		
					IG	CG		IG	CG	IG	CG	Type	SupplementDose (g/day)	CG
Claessens et al., 2009 (a) [42]	Netherlands	R, P, SB, PC	Individuals with OW & OB	♂/♀	14	16	12	45.4 ± 8.2	46 ± 8.8	32.9 ± 6	32.4 ± 4.8	CP	50	MD
Claessens et al., 2009 (b) [42]	Netherlands	R, P, SB, PC	Individuals with OW & OB	♂/♀	18	16	12	44.9 ± 8.5	46 ± 8.8	33.4 ± 4	32.4 ± 4.8	WP	50	MD
Pal et al., 2010 (a) [43]	Australia	R, P, SB, PC	Individuals with OW & OB	♂/♀	25	25	12	48.5 ± 1	48.4 ± 7.5	32 ± 4	30.6 ± 4	WP	54	CHO
Pal et al., 2010 (b) [43]	Australia	R, P, SB, PC	Individuals with OW & OB	♂/♀	20	25	12	48 ± 10.5	48.4 ± 7.5	31.3 ± 4.5	30.6 ± 4	CP	54	CHO
Fluegel et al., 2010 [44]	USA	R, P, PC	Patients with HTN & pre-HTN	♂/♀	36	35	6	20.4 ± 1.7	20.7 ± 1.9	25.1 ± 2.6	24.2 ± 2.4	WP	28	Non-hydrolyzed WP beverage
Takahira et al., 2011 [45]	Japan	R, P, DB, PC	Individuals with visceral fat OB	♂/♀	23	21	32	54.4 ± 13	56.8 ± 12.2	29.3 ± 3.8	29 ± 4.5	MP	22	Soy PR
Aldrich et al., 2011 [46]	USA	R, P, CO	Midlife adults	♂/♀	5	5	20	49.2 ± 3.9	51.3 ± 5.1	30.6 ± 1.5	29.9 ± 1.5	WP	45	Control diet
Hodgson et al., 2012 [103]	Australia	R, P, DB, PC	Older women	♀	93	87	96	74.3 ± 2.7	74.3 ± 2.6	26.3 ± 3.8	27.2 ± 3.9	WP	30	Low-PR, high-CHO beverage
Gouni-Berthold et al., 2012 [47]	Germany	R, P, DB, PC	Patients with MetS	♂/♀	83	88	12	52.9 ± 10.3	53.9 ± 9.5	30.8 ± 4.2	31.3 ± 4	WP	15.3	Yogurt
Agin et al., 2001 [104]	USA	R, P, CO	Women with HIV	♀	10	10	14	43.4 ± 10.6	41 ± 10.2	23 ± 2.3	24.8 ± 2.5	WP	57	PRE
Ahmadi Kani Golzar et al., 2012 [128]	Iran	R, P, SB, PC	Young men with OW	♂	10	10	6	22.7± 2.3	21.2± 1.0	26.5 ± 1.1	27.1 ± 1.5	WP	30	Starch solution
Sheikholeslami Vatani et al., 2012 [129]	Iran	R, P, SB, PC	Young men with OW	♂	9	10	6	23 ± 2	21 ± 1	26.5 ± 1.2	27.2 ± 1.6	WP	90	Starch solution
Figueroa et al., 2014 (a) [105]	USA	R, P, DB, PC	Young women with OB & high BP	♀	11	11	4	31 ± 9.9	31 ± 6.6	37.9 ± 6.6	33.5 ± 4	CP	30	MD
Figueroa et al., 2014 (b) [105]	USA	R, P, DB, PC	Young women with OB & high BP	♀	11	11	4	28 ± 3.3	31 ± 6.6	34.3 ± 4.6	33.5 ± 4	WP	30	MD
Tahavorgar et al., 2015 [130]	Iran	R, P, DB, PC	Men with OW & OB	♂	26	19	12	39.4 ± 6.9	38.8 ± 8.8	32.1 ± 3.2	32.1 ± 2.7	WP	65	Soy PR
Arciero et al., 2016 [48]	USA	R, P, CO	Individuals with OW	♂/♀	12	9	16	48 ± 1	52 ± 4	32 ± 7	33 ± 3	WP	20–25	Food PR
Larsen et al., 2018 [49]	Denmark	R, P, SB, PC	Individuals with OW & OB	♂/♀	14	15	4	41	41	34.9 ± 5.4	35.1 ± 5.8	WP	41	MD
Demling & DeSanti 2000 (a) [50]	USA	R, P, CO	Police officers with OW	♂/♀	14	10	12	33 ± 4	35 ± 4	30 ± 3.9	29 ± 3.5	CP	74	Hypocaloric diet
Demling & DeSanti 2000 (b) [50]	USA	R, P, CO	Police officers with OW	♂/♀	14	10	12	34 ± 3	35 ± 4	31 ± 4.5	29 ± 3.5	WP	74	Hypocaloric diet
Grey et al., 2003 (a) [51]	Canada	R, P, DB, PC	Patients with CF	♂/♀	10	11	12	25.5 ± 6.3	24.2 ± 3.9	21 ± 4.3	20.6 ± 2.7	WP	20	CP
Grey et al., 2003 (b) [51]	Canada	R, P, DB, PC	Patients with CF	♂/♀	11	10	12	24.2 ± 3.9	25.5 ± 6.3	20.6 ± 2.7	21 ± 4.3	CP	20	WP
Nabuco et al., 2019 [106]	Brazil	R, P, DB, PC	Older women with sarcopenic OB	♀	13	13	12	68 ± 4.2	70.1 ± 3.9	26.4 ± 3	27.4 ± 3	WP	15	MD
Moon et al., 2020 [131]	USA	R, P, DB, PC	Trained men	♂	12	12	8	32.8 ± 6.7	32.8 ± 6.7	27.2 ± 1.9	27.8 ± 1.9	WP	24	Rice
Lefferts et al., 2020 [52]	USA	R, P, DB, PC	Older adults	♂/♀	53	46	12	69 ± 7	67 ± 6	27.9 ± 5.6	27 ± 3.9	WP	50	MD
Hudson et al., 2020 [53]	USA	R, P, DB, PC	Individuals with OW & OB	♂/♀	21	23	16	53 ± 9.2	52 ± 4.8	31 ± 3.2	30.3 ± 3.4	MP	64	MD
Fuglsang-Nielsen et al., 2021 (a) [54]	Denmark	R, P, DB, PC	Individuals with abdominal OB	♂/♀	15	16	12	64	64	29.7 ± 3.9	30.1 ± 3.7	WP+ low fiber	60	MD
Fuglsang-Nielsen et al., 2021 (b) [54]	Denmark	R, P, DB, PC	Individuals with abdominal OB	♂/♀	17	17	12	64	64	29.1 ± 3.4	28.7 ± 3.8	WP+ high fiber	60	MD
Weinheimer et al., 2012 (a) [55]	USA	R, P, DB, PC	Individuals with OW & OB	♂/♀	81	84	36	47± 8.1	49 ± 7	30.4 ± 2.6	29.9 ± 2.7	WP	20	MD
Weinheimer et al., 2012 (b) [55]	USA	R, P, DB, PC	Individuals with OW & OB	♂/♀	25	84	36	46 ± 9.4	49 ± 7	29.4 ± 2.3	29.9 ± 2.7	WP	40	MD
Weinheimer et al., 2012 (c) [55]	USA	R, P, DB, PC	Individuals with OW & OB	♂/♀	30	84	36	50 ± 7.1	49 ± 7	30.7 ± 3.4	29.9 ± 2.7	WP	60	MD
Kjølbæk et al., 2017 [56]	Denmark	R, P, DB, PC	Individuals with OW & OB	♂/♀	39	38	16	41.2 ± 10.2	38.3 ± 11.5	28.5 ± 3.1	28.9 ± 2.7	WP	45	MD
Jeong et al., 2019 [57]	USA	R, P, PC	Hemodialysis patients	♂/♀	38	34	48	56.6 ± 13.0	54.4 ± 12.3	30.6 ± 7.1	31.5 ± 7.6	WP	12.85	Non-nutritive beverage
Yang et al., 2019 (a) [58]	China	R, P, PC	Individuals with pre- or mild HTN, and normal weight	♂/♀	12	12	12	42.3 ± 11.6	43.8 ± 11.7	24.1 ± 3.1	24.3 ± 2.3	WP	30	MD
Yang et al., 2019 (b) [58]	China	R, P, PC	Individuals with pre- or mild HTN, OW, and OB	♂/♀	15	15	12	42.3 ± 11.6	43.8 ± 11.7	24.1 ± 3.1	24.3 ± 2.3	WP	30	MD
Kataoka et al., 2016 [132]	Japan	R, P, PC	Patients with HTN	♂	10	11	8	69 ± 3.1	69 ± 3.3	22 ± 3.1	23 ± 3.3	WP	4.28	CHO
Ormsbee et al., 2015 (a) [107]	USA	R, P, DB, PC	Women with OW & OB	♀	13	10	4	29.3 ± 4.3	27.7 ± 7.3	34.4 ± 4.7	33.1 ± 5.4	WP	30	MD
Ormsbee et al., 2015 (b) [107]	USA	R, P, DB, PC	Women with OW & OB	♀	14	10	4	30.0 ± 7.1	27.7 ± 7.3	36.5 ± 6.7	33.1 ± 5.4	CP	30	MD
Sun et al., 2022 (a) [108]	China	R, P, SB, CO	Older women	♀	16	18	8	61.3 ± 7.7		27.2 ± 1.6		WP + ERD	15.2	ERD
Sun et al., 2022 (b) [108]	China	R, P, SB, CO	Older women	♀	14	18	8	61.3 ± 7.7		27.2 ± 1.6		WPH + RD	16.8	ERD
Nouri et al., 2022 [109]	Iran	R, P, DB, PC	Women with OW, OB & T2DM	♀	18	17	12	44.0 ± 6.2	46.9 ± 5.1	32.5 ± 4.2	31.6 ± 5.0	WP	20	Unfortified bread
Nabuco et al., 2019 [110]	Brazil	R, P, DB, PC	Older women	♀	15	15	12	69.2 ± 4.1	68.4 ± 4.5	27.4 ± 5.1	26.6 ± 3.4	WP	15	MD
Frestedt et al., 2008 [59]	USA	R, P, DB, PC	Individuals with OB	♂/♀	31	28	12	43.6 ± 6.1	42 ± 6.3	35.7 ± 3.9	35.4 ± 3.7	WP	20	MD
Silva et al., 2010 [60]	Brazil	R, P, DB, PC	Patients with ALS	♂/♀	8	8	16	53	53	21.7 ± 1.1	22.9 ± 1.1	WP	22	MD
Sohrabi et al., 2016 [61]	Iran	R, P, CO	Hemodialysis patients	♂/♀	23	23	8	57 ± 9.6	55 ± 6.5	24.3 ± 4.2	22.4 ± 3.5	WP	6.42	NI
Sharp et al., 2018 [62]	USA	R, P, DB, PC	Healthy individuals	♂/♀	10	10	8	19 ± 2	21 ± 2	25.4 ± 4.8	25.2 ± 3.8	WP	46	MD
Bumrungpert et al., 2018 [63]	Thailand	R, P, DB, PC	Patients with cancer	♂/♀	23	19	12	54.1 ± 9.3	51.5 ± 9.6	24.9 ± 5.7	23.6 ± 3.7	WP	40	MD
Derosa et al., 2020 (a) [64]	Italy	R, P, DB, PC	Patients with T2DM	♂/♀	59	58	12	59.7 ± 9.1	58.6 ± 8.8	22.7 ± 2.1	22.7 ± 2.1	WP	5	CP
Derosa et al., 2020 (b) [64]	Italy	R, P, DB, PC	Patients with T2DM	♂/♀	58	59	12	58.6 ± 8.8	59.7 ± 9.1	22.7 ± 2.1	22.7 ± 2.1	CP	5	WP
Ahmadi et al., 2020 [65]	Iran	R, P, SB, CO	Patients with COPD	♂/♀	23	21	8	62.0 ± 7	63.4 ± 7.2	20.6 ± 3.4	21.5 ± 2.5	WP	15.9	Dietary advice
Burke et al., 2001 [133]	Canada	R, P, DB, PC	Healthy men	♂	10	5	6	18–31	18–31	NR	NR	WP	102	MD
Rankin et al., 2004 [134]	USA	R, P, PC	Healthy men	♂	10	9	10	20.5 ± 2	21 ± 1.4	NR	NR	MP	7.02	CHO
Samadi et al., 2021 [135]	Iran	R, P, DB, PC	Basketball players	♂	22	22	8	20–30	20–30	23.8 ± 2.3	22.8 ± 1.8	WP	25	Starch
Teixeira et al., 2022 [136]	Portugal	R, P, DB, PC	Futsal players	♂	20	20	8	18–35	18–35	NR	NR	WP	25	Plant-based PR
Pettersson et al., 2021 [137]	Sweden	R, P, DB, PC	Individuals with OW & OB	♂	10	10	6	28.2 ± 5.5	27.9 ± 5	29.8 ± 2.3	30.4 ± 1.8	MP	8.57	CHO
Gryson et al., 2014 [66]	France	R, P, DB, PC	Older adults	♂/♀	9	9	16	60.9± 0.5	60.5± 0.7	26.2 ± 1.8	26.8 ± 2.7	MP	10	4g MP
Hulmi et al., 2015 [138]	Finland	R, P, DB, PC	Healthy man	♂	22	21	12	31.4 ± 6.6	36.4 ± 19.2	25.6 ± 0.9	25.4 ± 0.9	WP	13	MD
Maltais et al., 2016 [139]	Canada	R, P, DB, PC	Patients with sarcopenia	♂	8	8	16	68 ± 5.1	64 ± 4.9	25.8 ± 3	27 ± 2.7	MP	13.53	Soy milk
Keogh & Clifton 2008 [67]	Australia	R, P, DB, PC	Individuals with OW & OB	♂/♀	34	38	52	49.6 ± 12.3	50.3 ± 12.4	34.4 ± 3.7	34.4 ± 3.7	WP	15	Skim milk
Fernandes et al., 2018 [111]	Brazil	R, P, DB, PC	Older women	♀	16	16	12	67.3 ± 4.1	67.8 ± 4	25.9 ± 2.7	25.4 ± 2.6	WP	15	MD
Rambousková et al., 2014 [68]	Czech Republic	R, P, CO	Older adults	♂/♀	23	24	8	84.2 ± 9.7	85.3 ± 9.2	20.3 ± 2.9	20.4 ± 2.8	MP	18.2	NI
Piccolo et al., 2015 [112]	USA	R, P, DB, PC	Women with OB	♀	16	11	8	41 ± 9.8	41 ± 9.8	36.9 ± 3.1	36 ± 4.8	WP	20	Gelatin
Brown et al., 2004 (a) [140]	USA	R, P, DB, PC	Healthy men	♂	9	9	9	20.3 ± 1	21.6± 0.2	25.0 ± 2.7	24.7 ± 2.4	WP	33	Soy PR
Brown et al., 2004 (b) [140]	USA	R, P, DB, CO	Healthy men	♂	9	9	9	20.3 ± 1	20.4 ± 1.9	25.0 ± 2.7	24.9 ± 0.8	WP	33	Training
Hartman et al., 2007 [141]	Canada	R, P, PC	Healthy men	♂	18	19	12	18–30	18–30	25.6 ± 3.6	23.9 ± 3.0	MP	12.5	CHO
Cribb et al., 2007 [142]	Australia	R, P, DB, PC	Male bodybuilders	♂	5	7	11	24 ± 5	24 ± 7	21.4 ± 3.9	24.3 ± 4.0	WP	105	CHO
Sattler et al., 2008 [69]	USA	R, P, DB, PC	Patients with HIV	♂/♀	29	30	12	41 ± 25.9	41 ± 23.7	20.7 ± 2.3	21.1 ± 2.8	WP	80	CHO
Eliot et al., 2008 [143]	USA	R, P, DB, PC	Middle-aged healthy men	♂	11	10	14	48–72	48–72	NR	NR	WP	15	Gatorade
Josse et al., 2010 [113]	Canada	R, P, SB, PC	Healthy women	♀	10	10	12	23.2 ± 8.9	22.4 ± 7.6	26.2 ± 13.3	25.2 ± 12	MP	25.71	MD
Mojtahedi et al., 2011 [114]	USA	R, P, DB, PC	Older women	♀	13	13	24	64.7 ± 4.4	64.6 ± 5.2	32.3 ± 3.9	32.7 ± 4.2	WP	50	MD
Arazi et al., 2011 [144]	Iran	R, P, DB, PC	Healthy men	♂	20	20	8	21.3 ± 1.2	22.5 ± 3.4	24.1 ± 1.3	23.9 ± 1.4	WP	131	Starch
Baer et al., 2011 [70]	USA	R, P, DB, PC	Individuals with OW & OB	♂/♀	23	25	23	49 ± 43.2	51 ± 45	31 ± 10.6	31.1 ± 12.5	WP	55	CHO
Elahikhah et al., 2024 [115]	Iran	R, P, SB, CO	Women with OB	♀	21	20	8	37.1 ± 5.7	36.7 ± 9.0	33.6 ± 2.9	35.0 ± 3.0	MPC	20	WLD
Giglio et al., 2019 [116]	Brazil	R, P, DB, PC	Women with OW	♀	17	20	8	37.8 ± 12	43 ± 8	31.1 ± 4	30.9 ± 3.6	WP	25	Collagen
DeNysschen et al., 2009 [145]	USA	R, P, DB, PC	Men with hyperlipidemia	♂	10	9	12	38		28.5 ± 2.1	27.9 ± 1.2	WP	26.6	CHO
Haidari et al., 2020 [117]	Iran	R, P, CO	Pre-menopausal women with OB	♀	30	30	8	31 ± 6.2	32.2 ± 5.1	33.5 ± 3.1	33.3 ± 2.6	WP+ WLD	30	WLD
Hambre et al., 2012 [146]	Sweden	R, P, CO	Healthy men	♂	12	12	12	24.2 ± 3.7	23.2 ± 3.4	22.6 ± 2.5	22.3 ± 1.9	WP	33	A meal of fast food
Ottestad et al., 2017 [71]	Norway	R, P, DB, PC	Older adults	♂/♀	17	19	12	76.8 ± 6.2	77.1 ± 4.7	27.6 ± 4.2	25.9 ± 4.9	MP	40	CHO
Lopes Gomes et al., 2017 [118]	Brazil	R, P, CO	Postmenopausal women	♀	15	15	16	41 ± 10	49 ± 10	36 ± 6	35 ± 4	WP	69	Hypocaloric diet
Sugawara et al., 2012 [72]	Japan	R, P, DB, CO	Patients with COPD	♂/♀	17	14	12	77.4 ± 5.2	77.1 ± 5.8	NR	NR	WP	20	Normal diet
Björkman et al., 2012 [73]	Finland	R, P, CO	Nursing home residents	♂/♀	46	51	24	84.1 ± 7.6	83 ± 8.7	24.8 ± 4.3	24 ± 5.5	WP	20	Regular fruit juice
Joy et al., 2013 [147]	USA	R, P, DB, PC	Resistance-trained men	♂	12	12	8	21.3 ± 19	21.3 ± 1.9	NR	NR	WP	28.57	MD
Herda et al., 2013 [148]	USA	R, P, DB, PC	Trained men	♂	22	21	8	21.0 ± 1.6	20.9 ± 1.7	23.6 ± 1.9	24.6 ± 4.3	WP	28.57	MD
Volek et al., 2013 [74]	USA	R, P, DB, PC	Non-resistance-trained men	♂/♀	19	22	36	22.8 ± 3.7	22.3 ± 3.1	25.1 ± 6.2	24.5 ± 5.8	WP	22	MD
Chalé et al., 2013 [75]	USA	R, P, DB, PC	Older adults	♂/♀	42	38	28	78 ± 4	77.3 ± 3.9	27 ± 3.2	26.9 ± 3.1	WP	40	MD
Babault et al., 2014 (a) [149]	France	R, P, DB, PC	Physically active men	♂	22	24	10	22.2 ± 3.9	22 ± 3.9	23.7 ± 3.5	23.4 ± 3.7	CP	24.8	MD
Babault et al., 2014 (b) [149]	France	R, P, DB, PC	Physically active men	♂	22	24	10	22.5 ± 4.1	22 ± 3.9	22.7 ± 2.4	23.4 ± 3.7	MP	24.8	MD
Duff et al., 2014 [76]	Canada	R, P, DB, PC	Individuals with AO	♂/♀	21	19	8	57.5 ± 6.3	61.8 ± 4.8	25.9 ± 7.3	26.9 ± 6.4	WP	38	Bovine colostrum
Zhu et al., 2015 [119]	Australia	R, P, DB, PC	Older women	♀	101	95	96	74.2 ± 2.8	74.3 ± 2.6	26.1 ± 3.8	27.2 ± 4	WP	30	Skim MP
Hulmi et al., 2009 [150]	Finland	R, P, DB, PC	Young men	♂	9	9	21	24.7 ± 5	27.4 ± 3.1	23.2 ± 2.5	23.2 ± 2.5	WP	8.5	PL
Kerstetter et al., 2015 [77]	USA	R, P, DB, PC	Older adults	♂/♀	106	102	72	69.9 ± 6.1	70.5 ± 6.4	26.1 ± 3.4	26.4 ± 4	WP	45	MD
Hector et al., 2015 [78]	Canada	R, P, DB, PC	Adults with OW & OB	♂/♀	7	12	2	52 ± 7.5	48 ± 10.4	34.7 ± 4.1	36.9 ± 4.1	WP	54	MD
Malekian et al., 2015 [79]	USA	R, P, PC	African American men & women	♂/♀	15	13	24	35 ± 4	32 ± 8	43 ± 8	43 ± 8	WP	56	Starch
Taylor et al., 2016 [120]	USA	R, P, DB, PC	Basketball players	♀	8	6	8	20 ± 2	21 ± 3	22.8 ± 1.9	23.8 ± 3.1	WP	27.4	MD
Reidy et al., 2016 [151]	USA	R, P, DB, PC	Young men	♂	18	18	12	25 ± 4.7	25 ± 4.8	25.8 ± 3.3	24.6 ± 2.9	WP	21.5	MD
Naclerio et al., 2017 [152]	UK	R, P, DB, PC	Master triathletes	♂	8	8	10	45.3 ± 8.9	46.2 ± 7	25.2 ± 4.4	23.8 ± 2.6	WP	20	MD
Naclerio et al., 2017 [153]	UK	R, P, DB, PC	Resistance-trained men	♂	8	8	8	26 ± 5	29 ± 9	23.1 ± 3.9	24.9 ± 5.1	WP	20	MD
Stojkovic et al., 2017 [154]	USA	R, P, DB, PC	Post-menopausal women	♂	38	46	72	68.9 ± 5.5	69.3 ± 6.1	26 ± 3.7	25.8 ± 4.1	WP	20	MD
Hwang et al., 2017 [155]	USA	R, P, DB, PC	Resistance-trained men	♂	11	9	10	20.9 ± 1.3	21 ± 1.1	25.1 ± 3.7	24.4 ± 3.1	WP	25	MD
Dudgeon et al., 2017 [156]	USA	R, P, SB, PC	Resistance-trained men	♂	8	8	8	24 ± 1.6	24 ± 1.6	NR	NR	WP	32	CHO
Mobley et al., 2017 (a) [157]	USA	R, P, DB, PC	College-aged men	♂	17	15	12	21 ± 4.1	21 ± 3.9	NR	NR	WP	50	MD
Mobley et al., 2017 (b) [157]	USA	R, P, DB, PC	College-aged men	♂	14	15	12	21 ± 3.7	21 ± 3.9	NR	NR	WP	50	MD
Reimer et al., 2017 (a) [80]	Canada	R, P, DB, PC	Adults with OW& OB	♂/♀	22	26	12	38.7 ± 12.1	40.4 ± 13.6	31.5 ± 6.1	31.1 ± 4.5	WP	10	Prebiotic bar (ITF)
Reimer et al., 2017 (b) [80]	Canada	R, P, DB, CO	Adults with OW& OB	♂/♀	21	27	12	40.7 ± 15.5	39.8 ± 12.6	31.7 ± 5.3	31.3 ± 6.1	WP	10	Control snack bar
Hassan & Hassan 2017 [81]	Israel	R, P, CO	Peritoneal dialysis patients	♂/♀	18	18	12	59.7 ± 11.5	58.1 ± 12.3	28.7 ± 3.3	28.6 ± 3.5	WP	26.3	PR without WP
Hassan 2017 [82]	Israel	R, P, CO	Peritoneal dialysis patients	♂/♀	19	17	12	58.4 ± 11.8	56.9 ± 12.6	28.3 ± 3.3	27.9 ± 4.3	WP	28.1	PR without WP
Dirks et al., 2017 [83]	Netherland	R, P, DB, PC	Frail elderly	♂/♀	17	17	24	76 ± 8	77 ± 8	29.5 ± 4.8	28.6 ± 3.6	MP	30	PL
Gjevestad et al., 2017 [84]	Norway	R, P, DB, PC	Older adults	♂/♀	14	17	12	76.9 ± 4.9	77.7 ± 4.8	27.1 ± 3.8	26.4 ± 4.9	MP	40	CHO
Mori et al., 2018 [121]	Japan	R, P, SB, CO	Older adults	♀	25	25	24	70.6 ± 4.6	70.6 ± 4.2	22.1 ± 2.1	22.9 ± 2.9	WP	6.37	Exercise
Gaffney et al., 2018 [158]	New Zealand	R, P, DB, PC	Patients with T2DM	♂	12	12	10	53.5 ± 5.6	57.8 ± 5.2	29.6 ± 2.7	30.1 ± 4.9	WP	20	CHO
Holwerda et al., 2018 [159]	Netherlands	R, P, DB, PC	Active older men	♂	21	20	12	69 ± 4.6	71 ± 4.5	25.5 ± 2.7	245.1 ± 2.2	WP	30	PL
Englund et al., 2018 [85]	USA & Sweden	R, P, DB, PC	Older adults	♂/♀	60	57	24	78.1 ± 5.8	76.9 ± 4.9	27.9 ± 3.3	28.4 ± 3.9	WP	20	Nonnutritive sweetened drink
Sahathevan et al., 2018 [86]	Malaysia	R, P, CO	Peritoneal dialysis patients	♂/♀	37	37	24	50.8 ± 15.2	42.1 ± 14.5	21.6 ± 2.8	21.2 ± 2.3	WP	27.4	Dietary counseling
Park et al., 2019 [171]	South Korea	R, P, DB, PC	Young men	♂	10	8	12	37.8 ± 12	43 ± 8	22.6 ± 2.9	25.1 ± 2.6	WP	17.4	PL
Forbes et al., 2019 [161]	Canada	R, P, DB, PC	Healthy men	♂	9	9	6	27 ± 7	27 ± 7	NR	NR	WP	79	CHO
Amasene et al., 2019 [87]	Spain	R, P, DB, PC	Post-hospitalized older adults	♂/♀	15	13	12	82.9 ± 5.5	81.7 ± 6.4	27.4 ± 3.5	30.8 ± 6.5	WP	5.7	PL
Cereda et al., 2019 [88]	Italy	R, P, CO	Malnourished advanced cancer patients	♂/♀	82	84	12	65.1 ± 11.7	65.7 ± 11.4	22 ± 4.1	22.3 ± 3.9	WP	20	Nutritional counseling
Ten Haaf et al., 2019 [89]	Netherlands	R, P, DB, PC	Physically active older adults	♂/♀	58	56	12	69 ± 3.7	69 ± 4.4	27.2 ± 2.6	26.3 ± 2.5	MP	31	PL
Kang et al., 2019 [90]	China	R, P, CO	Frail older adults	♂/♀	66	49	12	76.7 ± 7.11	78.0 ± 6.8	21.0 ± 3.4	22.7 ± 4.4	WP	32.4	Resistance exercise
Rakvaag et al., 2019 (a) [91]	Denmark	R, P, DB, PC	Adults with AO	♂/♀	15	16	12	67 ± 6.7	62 ± 7.4	28.4 ± 4.1	30.3 ± 4.5	WP + low fiber	60	MD + low fiber
Rakvaag et al., 2019 (b) [91]	Denmark	R, P, DB, PC	Adults with AO	♂/♀	17	17	12	65 ± 6.7	64 ± 8.1	29.6 ± 2.3	29.1 ± 3.6	WP + high fiber	60	MD+ high fiber
Brown et al., 2020 [122]	USA	R, P, DB, PC	Female collegiate dancers	♀	10	11	12	19.9 ± 0.7	19.4 ± 1.5	21.7 ± 2.4	21.8 ± 1.9	WP	75	MD
McAdam et al., 2022 [162]	USA	R, P, DB, PC	Army soldiers	♂	39	42	9	21 ± 3	23 ± 4	25.7 ± 4.4	25.4 ± 5.1	WP	38.6	CHO
McAdam et al., 2018 [160]	USA	R, P, DB, PC	Army soldiers	♂	34	35	8	19 ± 1	19 ± 1	24.5 ± 4.2	24.1 ± 3.7	WP	77	CHO
Obradović et al., 2020 [163]	Serbia	R, P, PC	Male college athletes	♂	10	10	8	23 ± 4	23 ± 4	25.0 ± 1.7	24.7 ± 1.4	WP	45.4	MD
Lynch et al., 2020 [92]	USA	R, P, DB, PC	Untrained young individuals	♂/♀	19	26	12	18–35	18–35	18.5–29.9	18.5–29.9	WP	19	Soy PR
Boutry-Regard et al., 2020 [93]	Japan	R, P, DB, PC	Elderly adults	♂/♀	15	12	12	78 ± 3.9	78 ± 6.9	21.3 ± 3.5	20.8 ± 2.8	WP	20	MD
Mori et al., 2021 [123]	Japan	R, P, CO	Older women with sarcopenia	♀	20	19	24	78.1 ± 2.7	78.1 ± 4.6	20.3 ± 2.5	19.6 ± 2.2	WP	3.14	Exercise
Biesek et al., 2021 [124]	Brazil	R, P, SB, PC	Older women	♀	16	15	12	73.1 ± 5.3	70.4 ± 3.9	28.1 ± 3.8	27.1 ± 4.3	WP	21	MD
Dulac et al., 2021 (a) [164]	Canada	R, P, DB, PC	Older men	♂	21	19	12	68·3 ± 5.3	70.7 ± 8.6	26.7 ± 3	25.4 ± 3.4	WP	30	MD
Dulac et al., 2021 (b) [164]	Canada	R, P, DB, PC	Older men	♂	20	19	12	69 ± 6.1	70.7 ± 8.6	26 ± 3.5	25.4 ± 3.4	CP	30	MD
Roberson et al., 2021 [165]	USA	R, P, DB, PC	College-aged men	♂	17	12	12	21 ± 2	21 ± 1	NR	NR	WP	52.6	MD
Nakayama et al., 2021 [94]	Japan	R, P, DB, PC	Healthy older adults	♂/♀	61	61	24	71.4 ± 6.2	70.4 ± 5.5	23.1 ± 3.1	22.8 ± 3.1	MP	10	PL
Koopmans et al., 2024 [190]	Netherlands	R, P, DB, PC	Physically active older adults	♂/♀	23	20	11	70 ±5	68 ± 5	24.4 ± 2.3	23.8 ± 2.7	WP	30	MD
Azhar et al., 2021 [95]	USA	R, P, DB, CO	Older adults	♂/♀	32	29	12	66–86	66–86	31.7 ± 6.1	32.7 ± 1.5	WP	15	Nutrition education
Li et al., 2021 [96]	China	R, P, CO	Older adults with low lean mass	♂/♀	16	30	24	71 ± 4	71 ± 4	21.8 ± 2	20.8 ± 2.2	WP	16	NI
Mizubuti et al., 2021 (a) [97]	Brazil	R, P, DB, PC	Patients with chronic liver disease	♂/♀	35	40	2	51.6 ± 9.4	52.6 ± 11.4	NR	NR	WP	40	CP
Mizubuti et al., 2021 (b) [97]	Brazil	R, P, DB, PC	Patients with chronic liver disease	♂/♀	40	35	2	52.6 ± 11.4	51.6 ± 9.4	NR	NR	CP	40	WP
Mertz et al., 2021 [98]	Denmark	R, P, DB, PC	Healthy older adults	♂/♀	44	34	48	70.3 ± 4.3	69.6 ± 3.9	25.2 ± 3.6	26 ± 3.9	WP	40	MD+ sucrose
Bach et al., 2022 [99]	Brazil	R, P, DB, PC	Older adults	♂/♀	15	16	12	66.9 ± 4.3	65.8 ± 5.0	26.3 ± 2.2	25.4 ± 2.0	WP	40	MD
Henriques et al., 2023 [125]	Brazil	R, P, DB, PC	Patients underwent bariatric surgery	♀	17	15	8	46 ± 8.26	47.6 ± 7.4	31.2 ± 3.1	32.9 ± 6.3	WP	30	MD
Zbinden-Foncea et al., 2023 [166]	Chile	R, P, SB, PC	Untrained young men	♂	6	6	8	22.4 ± 3.1		22.3 ± 1.9		WP	9.85	Sugar-free orange juice
Yapici et al., 2023 [167]	Saudi Arabia	R, P, CO	Untrained young men	♂	11	11	8	20.9 ± 0.9	19.8 ± 0.7	21.7 ± 1.4	23.8 ± 1.1	MP	12.85	Resistance training program
Kim et al., 2023 [168]	South Korea	R, P, DB, PC	Healthy sedentary men	♂	17	15	12	23.5 ± 2.7	24.5 ± 3.3	24 ± 1.2	24.3 ± 1.8	WP	60	CHO
Zong et al., 2023 [100]	China	R, P, CO	Elderly inpatients with COPD	♂/♀	27	29	12	80.5 ± 7.7	81.1 ± 12	21.6 ± 3.6	24.6 ± 3.8	WP	20	Low-intensity exercise
Nouri et al., 2024 [126]	Iran	R, P, DB, PC	Women with T2DM, OW, & OB	♀	18	17	12	44 ± 6.2	46.9 ± 5.1	32.5 ± 4.2	31.6 ± 5.0	WP	20	Unfortified bread
Furtado et al., 2024 [101]	Brazil	R, P, DB, PC	Older adults with T2DM	♂/♀	19	20	12	68.0 ± 5.7	66.6 ± 6.3	30.3 ± 6.1	30.7 ± 6.1	WP	9.42	MD
Kemmler et al., 2018 [169]	Germany	R, P, CO	Patients with sarcopenic OB	♂	33	34	16	78.1 ± 5.4	76.9 ± 5.2	26.3 ± 2.5	26 ± 2.5	WP	137	NI
Kirk et al., 2020 (a) [189]	UK	R, P, CO	Older adults	♂/♀	22	24	16	69 ± 6	66 ± 4	27.4 ± 4.9	28.1 ± 7.4	WP	111	Exercise
Kirk et al., 2020 (b) [189]	UK	R, P, CO	Older adults	♂/♀	23	31	16	72 ± 6	68 ± 6	27.1 ± 4.1	26.2 ± 4.5	WP	111	NI
Santos et al., 2023 [102]	Brazil	R, P, SB, PC	Patients with CHD	♂/♀	15	10	12	64 ± 4.4	61 ± 14.8	28.6 ± 4.6	26.8 ± 3.5	WPI	30	MD
Kasim-Karakas et al., 2009 [127]	USA	R, P, SB, PC	Women with PCOS, OW& OB	♀	11	13	8	28 ± 3		38.9 ± 1.6	35.4 ± 1.2	WP	60	CHO
Lockwood et al., 2017 (a) [170]	USA	R, P, DB, PC	Healthy men	♂	15	15	8	21.8 ± 3.5	20.9 ± 1.5	24.9 ± 8.5	23.8 ± 8.5	WPC-L	60	CHO
Lockwood et al., 2017 (b) [170]	USA	R, P, DB, PC	Healthy men	♂	13	15	8	21.3 ± 2.5	20.9 ± 1.5	25.9 ± 5.8	23.8 ± 8.5	WPC	60	CHO
Lockwood et al., 2017 (c) [170]	USA	R, P, DB, PC	Healthy men	♂	13	15	8	21.5 ± 3.2	20.9 ± 1.5	25.1 ± 6.5	23.8 ± 8.5	WPH	60	CHO
Knuiman et al., 2019 [172]	Netherlands	R, P, DB, PC	Recreationally active men	♂	19	21	10	21.5 ± 1.7	22.5 ± 2.3	22.3 ± 1.7	22.4 ± 1.4	CP	41	CHO
Mhamed et al., 2024 [173]	Tunisia	R, P, CO	Well-trained endurance athletes	♂	20	9	8	NR	NR	19.7 ± 0.6	20.2 ± 0.9	WP	30	NI
Bodaghabadi et al., 2023 [174]	Iran	R, P, CO	Women with OW	♀	26	21	2	37.8 ± 6.5	37.8 ± 6.5	26.9 ± 1.6	27.8 ± 1.7	CP	40	High PR, low-dairy diet
Soares et al., 2023 [175]	Brazil	R, P, TB, PC	Men with T2DM	♂	13	13	12	68.1 ± 4.5	68.9 ± 4.1	29.3 ± 2.6	26.8 ± 3.8	WPI	5.71	MD
Ferguson-Stegall et al., 2011 [176]	USA	R, P, PC	Untrained individuals	♂/♀	11	10	4.5	22.1 ± 2.3	21.3 ± 3.2	24.8 ± 1.5	25.7 ± 1.6	MP	5.24	Isocaloric fat
Wilborn et al., 2013 (a) [12]	USA	R, P, DB, PC	Collegiate female athletes	♀	8	8	8	20.0 ± 1.9	21.0 ± 2.8	26.4 ± 9.2	29.0 ± 11.0	WP	13.71	CP
Wilborn et al., 2013 (b) [12]	USA	R, P, DB, PC	Collegiate female athletes	♀	8	8	8	21.0 ± 2.8	20.0 ± 1.9	29.0 ± 11.0	26.4 ± 9.2	CP	13.71	WP
Reljic et al., 2022 [177]	Germany	R, P, DB, PC	Sedentary, healthy adults	♂/♀	19	20	8	30.0 ± 7.8	32.5 ± 8.0	24.4 ± 3.2	24.9± 3.8	WP	18.6	MD
Reljic et al., 2024 [178]	Germany	R, P, DB, PC	Untrained healthy adults	♂/♀	19	17	8	26 ± 4	27 ± 6	21.8 ± 2.2	25.0 ± 4.3	WP	12.4	MD
Murray et al., 2025 [179]	New Zealand	R, P, DB, PC	Pre-menopausal women	♀	15	12	12	34.2 ±9.1	32.8 ± 9.7	24 ± 3.9	27.1 ± 3.1	WP	17.14	Milo powder
Yıldız et al., 2025 (a) [180]	Turkey	R, P, CO	Individuals underwent laparoscopic SG	♂/♀	15	15	12	35.1 ± 9.7	35.1 ± 9.7	42.3 ± 6.1	41.0 ± 3.1	CP	15	Standard PR diet
Yıldız et al., 2025 (b) [180]	Turkey	R, P, CO	Individuals underwent laparoscopic SG	♂/♀	15	15	12	35.1 ± 9.7	35.1 ± 9.7	41.0 ± 5.2	41.0 ± 3.1	CP	15	Standard PR diet
Sabooni et al., 2025 [181]	Iran	R, P, DB, PC	Patients underwent OAGB	♂/♀	39	39	12	40.5 ± 10.7	41.4 ± 8.6	46.4 ± 5.6	43.4 ± 2.9	WP	22.6	PL (no PR)
Ormsbee et al., 2018 [183]	USA	R, P, PC	Sedentary individuals	♂/♀	29	22	24	21.0 ± 3.2	20.3 ± 2.3	23.6 ± 1.5	25.7 ± 1.6	MP	84	CHD
Jonvik et al., 2019 [182]	Netherlands	R, P, DB, PC	Active men	♂	30	26	12	26 ± 6	26 ± 6	23.8 ± 2.9	24.3 ± 2.3	CP	41	CHD
Griffen et al., 2022 (a) [184]	UK	R, P, DB, PC	Healthy, active older men	♂	9	9	12	68 ± 3	67 ± 3	26.6 ± 2.4	25.1 ± 2.7	WP	50	MD
Griffen et al., 2022 (b) [184]	UK	R, P, DB, PC	Healthy, active older men	♂	9	9	12	66 ± 6	67 ± 6	25.0 ± 1.8	25.1 ± 3	WP	50	MD
Arnarson et al., 2013 [185]	Iceland	R, P, DB, PC	Elderly people	♂/♀	75	66	12	73.3 ± 6.0	74.6 ± 5.8	28.1 ± 4.4	29.4 ± 4.8	WP	8.57	CHO
Karelis et al., 2015 (a) [186]	Canada	R, P, DB, PC	Non-frail elderly individuals	♂/♀	34	33	19.3	69.9 ± 3.6	71.0 ± 4.6	24.9 ± 2.8	25.4 ± 2.8	WP	20	CP
Karelis et al., 2015 (b) [186]	Canada	R, P, DB, PC	Non-frail elderly individuals	♂/♀	33	34	19.3	71.0 ± 4.6	69.9 ± 3.6	25.4 ± 2.8	24.9 ± 2.8	CP	20	Cysteine-rich WP
Kirk et al., 2019 [187]	UK	R, P, CO	Older adults	♂/♀	22	24	16	69 ± 6	66 ± 4	27.4 ± 4.9	28.1 ± 7.4	WP	111.3	Exercise
Michel et al., 2022 [188]	USA	R, P, CO	Older adults	♂/♀	9	9	10	67.3 ± 8.9	72.1 ± 7.1	24.3 ± 4.3	27.2 ± 5.4	WP	75	Regular diet

Abbreviations: PC, placebo-controlled; SB, single-blinded; OB, obesity; DB, double-blinded; OW, overweight; CO, controlled; ♀, female; ♂, male; BP, blood pressure; TB, triple-blinded; BMI, body mass index; AO, abdominal obesity; HTN, hypertension; MetS, metabolic syndrome; PCOS, polycystic ovary syndrome; CHD, chronic heart disease; HIV, human immunodeficiency virus; WPH, whey protein hydrolysate; COPD, chronic obstructive pulmonary disease; ERD, energy-restricted diet; PRE, progressive resistance exercise; ITF, inulin-type fructans; WPC, whey protein concentrate; WP, whey protein; WPC-L, high-lactoferrin-containing whey protein concentrate; MP, milk protein; T2DM, type 2 diabetes mellitus; CF, cystic fibrosis; MPC, milk protein concentrate; CP, casein protein; CHO, carbohydrate; R, randomized; IG, intervention group; MD, maltodextrin; CG, control group; PL, placebo; P, parallel design; ALS, amyotrophic lateral sclerosis; SG, sleeve gastrectomy; WPI, whey protein isolate; USA, United States of America; OAGB, one anastomosis gastric bypass; NR, not reported; UK, United Kingdom; NI, no intervention; PR, protein; WLD, weight-loss diet.

**Table 2:** Subgroup analyses of the effects of MP supplementation on BC and anthropometric parameters.

Sub-Groups	Number of Effect Sizes	WMD (95%CI)	*p*-Value	Heterogeneity		
				*p*-Value Heterogeneity	*I*^2^ (%)	*p*-Value Between Sub-Groups
**BW (kg)**						
Overall effect	133	−22 (−0.52, 0.09)	0.160	<0.001	38.3	
Trial duration (weeks)						
>8	92	−0.14 (−0.53, 0.24)	0.459	<0.001	50.7	0.780
≤8	41	−0.22 (−0.63, 0.18)	0.279	0.900	0	
Intervention type						
CP	11	−0.32 (−1.08, 0.44)	0.408	0.999	0	0.902
WP	104	−0.22 (−0.61, 0.15)	0.248	<0.001	48.9	
MP	18	−0.14 (−0.53, 0.25)	0.489	0.877	0	
Supplement dose (g/day)						
>30	53	−0.13 (−0.42, 0.15)	0.356	0.530	0	0.995
≤30	80	−0.13 (−0.58, 0.31)	0.547	<0.001	51.6	
Baseline BMI						
Normal	56	0.13 (−0.14, 0.40)	0.346	0.335	6.6	0.005
OW	40	0.09 (−0.24, 0.44)	0.585	0.863	0.0	
OB	37	−1.50 (−2.46, −0.55)	**0.002**	<0.001	50.1	
Sex						
Both	67	−0.06 (−0.54, 0.41)	0.793	<0.001	60.0	0.239
Female	25	−0.55 (−1.01, −0.09)	**0.019**	0.811	0	
Male	41	−0.07 (−0.50, 0.34)	0.714	0.931	0	
Health status						
Healthy	110	−0.31 (−0.67, 0.05)	0.092	<0.001	39.9	0.290
Unhealthy	23	0.04 (−0.50, 0.59)	0.876	0.071	32.0	
Age						
≤60	93	−0.50 (−0.89, −0.11)	**0.011**	<0.001	40.7	0.001
>60	40	0.42 (0.03, 0.80)	**0.031**	0.318	8.5	
**BMI (kg/m^2^)**						
Overall effect	70	−0.03 (−0.14, 0.09)	0.626	0.309	7.2	
Trial duration (weeks)						
>8	45	−0.07 (−0.19, 0.05)	0.268	0.872	0	0.988
≤8	25	−0.06 (−0.31, 0.17)	0.586	0.022	39.9	
Intervention type						
CP	9	−0.02 (−0.33, 0.27)	0.853	0.951	0	0.047
WP	54	0.01 (−0.11, 0.14)	0.802	0.286	9.1	
MP	7	−0.55 (−0.99, −0.12)	**0.012**	0.389	4.9	
Supplement dose (g/day)						
>30	21	−0.06 (−0.29, 0.17)	0.609	0.339	9.2	0.782
≤30	49	−0.02 (−0.15, 0.11)	0.739	0.315	8.0	
Baseline BMI						
Normal	20	0.05 (−0.18, 0.29)	0.655	0.002	53.7	0.462
OW	25	0.01 (−0.18, 0.20)	0.902	0.999	0	
OB	25	−0.16 (−0.42, 0.10)	0.237	0.436	1.8	
Sex						
Both	38	0.08 (−0.05, 0.23)	0.219	0.301	9.6	0.010
Female	16	−0.36 (−0.65, −0.08)	**0.012**	0.811	0	
Male	16	−0.17 (−0.42, 0.08)	0.187	0.451	0	
Health status						
Healthy	50	−0.05 (−0.20, 0.09)	0.477	0.964	0	0.390
Unhealthy	20	0.08 (−0.19, 0.35)	0.552	0.002	54.2	
Age						
≤60	47	−0.02 (−0.18, 0.13)	0.779	0.040	28.2	0.800
>60	23	−0.05 (−0.28, 0.17)	0.612	0.984	0	
**WC (cm)**						
Overall effect	45	−0.69 (−1.16, −0.22)	**0.004**	<0.001	47.9	
Trial duration (weeks)						
>8	31	−0.84 (−1.40, −0.29)	**0.003**	<0.001	56.6	0.216
≤8	14	−0.19 (−1.06, 0.66)	0.652	0.326	11.6	
Intervention type						
CP	6	−0.25 (−0.73, 0.21)	0.286	0.955	0	0.481
WP	36	−0.71 (−1.29, −0.13)	**0.016**	<0.001	54.8	
MP	3	−0.69 (−3.33, 1.94)	0.605	0.096	57.4	
Supplement dose (g/day)						
>30	17	−1.19 (−2.22, −0.15)	**0.024**	<0.001	67.8	0.096
≤30	28	−0.27 (−0.59, 0.05)	0.105	0.404	4.0	
Baseline BMI						
Normal	6	−0.42 (−1.05, 0.20)	0.183	0.030	59.7	0.461
OW	14	−0.48 (−1.04, 0.07)	0.092	0.974	0	
OB	25	−1.15 (−2.18, −0.13)	**0.027**	<0.001	61.1	
Sex						
Both	25	−0.28 (−0.54, −0.02)	**0.035**	0.620	0	0.514
Female	15	−0.44 (−1.28, 0.38)	0.294	0.583	0	
Male	5	−2.06 (−5.24, 1.10)	0.202	<0.001	90.1	
Health status						
Healthy	35	−0.77 (−1.45, −0.09)	**0.026**	<0.001	48.5	0.416
Unhealthy	10	−0.42 (−0.94, 0.10)	0.114	0.110	37.3	
Age						
≤60	34	−0.73 (−1.29, −0.17)	**0.011**	<0.001	59.6	0.981
>60	11	−0.72 (−1.38, −0.06)	**0.032**	0.998	0	
**FM (kg)**						
Overall effect	111	−0.66 (−0.91, −0.41)	**<0.001**	<0.001	42.1	
Trial duration (weeks)						
>8	74	−0.44 (−0.70, −0.19)	**0.001**	0.098	18.0	0.042
≤8	37	−1.03 (−1.53, −0.52)	**<0.001**	<0.001	58.9	
Intervention type						
CP	12	−0.06 (−0.69, 0.55)	0.830	0.866	0	0.197
WP	85	−0.70 (−1.02, −0.38)	**<0.001**	<0.001	50.2	
MP	14	−0.52 (−0.81, −0.24)	**<0.001**	0.658	0	
Supplement dose (g/day)						
>30	51	−0.82 (−1.21, −0.43)	**<0.001**	<0.001	54.3	0.173
≤30	60	−0.47 (−0.78, −0.16)	**0.003**	0.054	23.8	
Baseline BMI						
Normal	35	−0.70 (−1.16, −0.24)	**0.003**	<0.001	63.8	0.014
OW	47	−0.25 (−0.50, −0.00)	**0.049**	0.965	0	
OB	29	−1.20 (−1.86, −0.54)	**<0.001**	0.022	37.8	
Sex						
Both	49	−0.28 (−0.50, −0.07)	**0.010**	0.833	0	0.017
Female	21	−1.09 (−1.71, −0.46)	**0.001**	0.005	50.2	
Male	41	−0.79 (−1.28, −0.31)	**0.001**	<0.001	55.8	
Health status						
Healthy	99	−0.72 (−0.98, −0.46)	**<0.001**	<0.001	42.7	0.109
Unhealthy	12	0.01 (−0.85, 0.88)	0.977	0.176	27.3	
Age						
≤60	78	−0.85 (−1.18, −0.53)	**<0.001**	<0.001	49.8	0.001
>60	33	−0.10 (−0.39, 0.18)	0.470	0.963	0	
**BFP (%)**						
Overall effect	80	−0.66 (−1.03, −0.28)	**0.001**	<0.001	71.2	
Trial duration (weeks)						
>8	55	−0.69 (−1.19, −0.19)	**0.006**	<0.001	76.8	0.466
≤8	25	−0.44 (−0.88, −0.00)	**0.046**	0.108	26.9	
Intervention type						
CP	9	−0.77 (−3.08, 1.53)	0.510	<0.001	94.5	0.475
WP	61	−0.73 (−1.09, −0.37)	**<0.001**	<0.001	45.4	
MP	10	−0.44 (−0.74, −0.15)	**0.003**	0.968	0	
Supplement dose (g/day)						
>30	27	−1.05 (−1.81, −0.29)	**0.006**	<0.001	88.5	0.268
≤30	53	−0.59 (−0.87, −0.32)	**<0.001**	0.716	0	
Baseline BMI						
Normal	28	−0.63 (−0.88, −0.39)	**<0.001**	0.860	0	0.100
OW	32	−0.28 (−0.57, 0.00)	0.051	0.659	0	
OB	20	−1.27 (−2.63, 0.08)	0.066	<0.001	89.1	
Sex						
Both	37	−0.75 (−1.36, −0.15)	**0.014**	<0.001	80.3	0.671
Female	14	−0.75 (−1.41, −0.09)	**0.026**	0.075	37.8	
Male	29	−0.41 (−1.02, 0.20)	0.188	<0.001	58.3	
Health status						
Healthy	70	−0.64 (−1.05, −0.22)	**0.003**	<0.001	73.7	0.344
Unhealthy	10	−0.98 (−1.57, −0.40)	**0.001**	0.328	12.4	
Age						
≤60	59	−0.69 (−1.16, −0.22)	**0.004**	<0.001	77.1	0.342
>60	21	−0.39 (−0.79, 0.00)	0.053	0.680	0	
**FFM (Kg)**						
Overall effect	40	0.67 (0.40, 0.94)	**<0.001**	0.483	0	
Trial duration (weeks)						
>8	26	0.81 (0.45, 1.17)	**<0.001**	0.881	0	0.194
≤8	14	0.34 (−0.26, 0.95)	0.272	0.087	36.1	
Intervention type						
CP	5	0.38 (−0.59, 1.36)	0.455	0.429	0	0.031
WP	32	0.84 (0.54, 1.15)	**<0.001**	0.805	0	
MP	3	−0.40 (−1.31, 0.50)	0.385	0.318	12.6	
Supplement dose (g/day)						
>30	15	0.43 (−0.25, 1.12)	0.213	0.099	33.6	0.682
≤30	25	0.59 (0.26, 0.93)	**0.001**	0.845	0	
Baseline BMI						
Normal	18	0.26 (−0.25, 0.78)	0.314	0.257	16.4	0.059
OW	13	0.54 (−0.04, 1.12)	0.069	<0.001	0	
OB	9	1.09 (0.62, 1.57)	**<0.001**	0.386	5.9	
Sex						
Both	16	0.64 (0.11, 1.18)	**0.018**	0.458	0	0.004
Female	6	1.15 (0.70, 1.60)	**<0.001**	0.385	5.0	
Male	18	0.05 (−0.41, 0.53)	0.816	0.987	0	
Health status						
Healthy	30	0.66 (0.37, 0.95)	**<0.001**	0.606	0	0.901
Unhealthy	10	0.72 (−0.24, 1.69)	0.141	0.197	26.9	
Age						
≤60	29	0.44 (0.02, 0.86)	**0.040**	0.149	21.7	0.311
>60	11	0.79 (0.25, 1.33)	**0.004**	0.988	0	
**LBM (kg)**						
Overall effect	65	0.41 (0.19, 0.62)	**<0.001**	0.036	25.5	
Trial duration (weeks)						
>8	46	0.32 (0.05, 0.58)	**0.018**	0.048	27.3	0.011
≤8	19	0.77 (0.55, 0.99)	**<0.001**	0.911	0	
Intervention type						
CP	7	1.10 (−0.62, 2.83)	0.211	0.002	71.6	0.560
WP	47	0.33 (0.09, 0.57)	**0.006**	0.132	19.0	
MP	11	0.46 (0.21, 0.72)	**<0.001**	0.906	0	
Supplement dose (g/day)						
>30	29	0.59 (0.18, 1.01)	**0.005**	0.012	41.1	0.271
≤30	36	0.34 (0.14, 0.53)	**<0.001**	0.399	4.1	
Baseline BMI						
Normal	20	0.64 (0.46, 0.81)	**<0.001**	0.607	0	0.002
OW	25	0.12 (−0.10, 0.35)	0.293	0.814	0	
OB	20	0.61 (−0.20, 1.42)	0.142	0.005	51.1	
Sex						
Both	37	0.27 (−0.02, 0.56)	0.069	0.008	39.7	0.026
Female	7	0.62 (0.19, 1.06)	**0.005**	0.844	0	
Male	21	0.86 (0.54, 1.17)	**<0.001**	0.842	0	
Health status						
Healthy	59	0.38 (0.14, 0.62)	**0.002**	0.037	26.2	0.072
Unhealthy	6	0.73 (0.44, 1.02)	**<0.001**	0.795	0	
Age						
≤60	41	0.65 (0.34, 0.96)	**<0.001**	0.073	25.4	0.024
>60	24	0.23 (0.03, 0.42)	**0.021**	0.433	2.1	
**MM (kg)**						
Overall effect	17	−0.07 (−0.33, 0.19)	0.588	1.000	0	
Trial duration (weeks)						
>8	12	−0.07 (−0.34, 0.19)	0.596	0.995	0	0.970
≤8	5	−0.05 (−0.96, 0.85)	0.906	0.995	0	
Intervention type						
CP	2	2.47 (−1.55, 6.49)	0.229	0.846	0	0.450
WP	14	−0.02 (−0.54, 0.49)	0.922	1.000	0	
MP	1	−0.10 (−0.39, 0.19)	0.511	-	-	
Supplement dose (g/day)						
>30	11	−0.08 (−0.34, 0.18)	0.542	1.000	0	0.708
≤30	6	0.14 (−1.03, 1.32)	0.805	0.858	0	
Baseline BMI						
Normal	7	−0.21 (−1.08, 0.66)	0.635	1.000	0	0.500
OW	7	−0.07 (−0.35, 0.19)	0.575	0.997	0	
OB	3	1.15 (−0.96, 3.27)	0.285	0.738	0	
Sex						
Both	10	−0.09 (−0.37, 0.18)	0.521	0.988	0	0.711
Male	7	0.04 (−0.63, 0.73)	0.888	0.998	0	
Health status						
Healthy	13	−0.09 (−0.35, 0.17)	0.492	1.000	0	0.329
Unhealthy	4	0.74 (−0.91, 2.39)	0.379	0.806	0	
Age						
≤60	11	0.03 (−0.68, 0.75)	0.929	0.995	0	0.761
>60	6	−0.08 (−0.36, 0.19)	0.539	0.992	0	

Abbreviations: MP, milk protein; BMI, body mass index; CI, confidence interval; MM, muscle mass; FM, fat mass; OW, overweight; WC, waist circumference; WP, whey protein; WMD, weighted mean difference; OB, obesity; LBM, lean body mass; CP, casein protein; FFM, fat-free mass; BC, body composition; BW, body weight; BFP, body fat percentage. Bold numbers indicate statistically significant differences (*p* < 0.05).

## Data Availability

The original contributions presented in this study are included in the article/Supplementary Materials. Further inquiries can be directed to the corresponding authors.

## Supplementary Materials

The following supporting information can be downloaded at https://www.mdpi.com/article/10.3390/nu17243877/s1. Figure S1: funnel plots; Figure S2: non-linear dose–response association between MP dose and mean differences in anthropometric parameters; Figure S3: non-linear dose–response association between duration of MP supplementation and mean differences in anthropometric parameters; Figure S4: linear dose–response association between MP dose and mean differences in anthropometric parameters; Figure S5: linear dose–response association between duration of MP supplementation and mean differences in anthropometric parameters; Table S1: Search strategy in MEDLINE (PubMed); Table S2: RoB assessment for included RCTs; Table S3: GRADE assessment.

## Abbreviations

The following abbreviations are used in this manuscript:

MP	Milk protein
BC	Body composition
MPC	Milk protein concentrate
WMD	Weighted mean difference
LBM	Lean body mass
WP	Whey protein
FFM	Fat-free mass
CI	Confidence interval
RT	Resistance training
WC	Waist circumference
FM	Fat mass
RCT	Randomized controlled trial
PROSPERO	Prospective Register of Systematic Reviews
BMI	Body mass index
PRISMA	Preferred Reporting Items for Systematic Reviews and Meta-Analyses
SD	Standard deviation
RoB	Risk of Bias
GRADE	Grading of Recommendations, Assessment, Development, and Evaluation
BFP	Body fat percentage
CP	Casein protein
BW	Body weight
AAs	Amino acids
BCAAs	Branched-chain amino acids
ALS	Amyotrophic lateral sclerosis
CF	Cystic fibrosis
CHD	Chronic heart disease
COPD	Chronic obstructive pulmonary disease
HIV	Human immunodeficiency virus
HTN	Hypertension
MetS	Metabolic syndrome
MM	Muscle mass
OAGB	One anastomosis gastric bypass
PCOS	Polycystic ovary syndrome
PICOS	Population, intervention, comparator, outcomes, study design
T2DM	Type 2 diabetes mellitus
WPH	Whey protein hydrolysates
GLP-1	Glucagon-like peptide-1
CCK	Cholecystokinin
BAT	Brown adipose tissue
WAT	White adipose tissue
OW	Overweight
OB	Obesity
AO	Abdominal obesity
BP	Blood pressure
WPI	Whey protein isolate
WPC	Whey protein concentrate
PL	Placebo
WPC-L	High-lactoferrin-containing WPC
ERD	Energy-restricted diet
CHO	Carbohydrate
MD	Maltodextrin
PRE	Progressive resistance exercise
ITF	Inulin-type fructans
SG	Sleeve gastrectomy
UK	United Kingdom
USA	United States of America
DPP-4	Dipeptidyl peptidase 4
