# Supplementary material for: Effects of Supplementation with Milk Proteins on Body Composition and Anthropometric Parameters: A Systematic Review and Dose–Response Meta-Analysis
Source: Nutrients. 2025 Dec 12;17(24):3877. doi: 10.3390/nu17243877 (PMC12736298; doi:10.3390/nu17243877)
Supplement: Supplementary file 1 [file nutrients-17-03877-s001.zip › nutrients-3994141-supplementary.pdf]

Supplementary Materials

Table S1. Search strategy in MEDLINE (PubMed)

	Keywords	Search strategy
#1	**Milk Proteins**	(“milk protein” [tiab] OR “milk proteins” [tiab] OR “milk protein supplementation” [tiab] OR “whey protein” [tiab] OR “whey” [tiab] OR “whey supplementation” [tiab] OR “casein” [tiab] OR “casein supplementation” [tiab] OR “milk protein concentrate” [tiab] OR “MPC” [tiab] OR “whey protein hydrolysate” [tiab] OR “WPH” [tiab] OR “dairy protein” [tiab])
#2	**Body composition and anthropometric parameters**	(“body composition” [tiab] OR “lean body mass” [tiab] OR “LBM” [tiab] OR “fat-free mass” [tiab] OR “FFM” [tiab] OR “fat mass” [tiab] OR “FM” [tiab] OR “body fat percentage” [tiab] OR “BFP” [tiab] OR “muscle mass” [tiab] OR “MM” [tiab] OR “body weight” [tiab] OR “BW” [tiab] OR “BMI” [tiab] OR “body mass index” [tiab] OR “waist circumference” [tiab] OR “WC” [tiab])
#3	**Study design**	(“randomized controlled trial” [tiab] OR “RCT” [tiab] OR “clinical trial” [tiab])
#4	**Final search combination**	#1 AND #2 AND #3

Table S2. Risk of bias assessment for included RCTs in the meta-analysis

Reference	Bias arising from the randomization process (Allocation bias)	Bias due to deviations from the intended interventions (Performance bias)	Bias due to missing outcome data (Attrition bias)	Bias in the measurement of the Outcome (Detection bias)	Bias in the selection of reported results (Reporting bias)	The overall risk of bias
Claessens et al. (2009)	H	U	L	L	U	H
Pal et al. (2010)	U	U	U	L	U	H
Fluegel et al. (2010)	H	U	L	L	U	H
Takahira et al. (2011)	L	L	L	L	L	L
Aldrich et al. (2011)	H	U	L	L	U	H
Hodgson et al. (2012)	L	L	L	L	L	L
Gouni-Berthold et al. (2012)	L	L	L	L	L	L
Agin et al. (2001)	U	U	U	L	U	H
Ahmadi Kani Golzar et al. (2012)	U	U	L	L	U	H
Sheikholeslami Vatani et al. (2012)	U	U	L	L	U	H
Figueroa et al. (2014)	L	L	L	L	L	L
Tahavorgar et al. (2015)	L	L	L	L	L	L
Arciero et al. (2016)	H	U	U	L	U	H
Larsen et al. (2018)	U	U	U	L	L	H
Demling & DeSanti (2000)	U	U	L	L	U	H
Grey et al. (2003)	L	L	L	L	L	L
Nabuco et al. (2019)	L	L	L	L	L	L
Moon et al. (2020)	L	L	L	L	L	L
Lefferts et al. (2020)	L	L	L	L	L	L
Hudson et al. (2020)	L	L	L	L	L	L
Fuglsang-Nielsen et al. (2021)	L	L	L	L	L	L
Weinheimer et al. (2012)	L	L	L	L	L	L
Kjølbæk et al. (2017)	L	L	L	L	L	L
Jeong et al. (2019)	H	U	L	L	U	H
Yang et al. (2019)	U	U	L	L	U	H
Kataoka et al. (2016)	H	U	L	L	U	H
Ormsbee et al. (2015)	L	L	L	L	L	L
Sun et al. (2022)	U	U	L	L	U	H
Nouri et al. (2022)	L	L	L	L	L	L
Nabuco et al. (2019)	L	L	L	L	L	L
Frestedt et al. (2008)	L	L	L	L	L	L

Table S2. *Cont.*

Reference	Bias arising from the randomization process (Allocation bias)	Bias due to deviations from the intended interventions (Performance bias)	Bias due to missing outcome data (Attrition bias)	Bias in the measurement of the Outcome (Detection bias)	Bias in the selection of reported results (Reporting bias)	The overall risk of bias
Silva et al. (2010)	L	L	L	L	L	L
Sohrabi et al. (2016)	U	U	L	L	U	H
Sharp et al. (2018)	L	L	L	L	L	L
Bumrungpert et al. (2018)	L	L	L	L	L	L
Derosa et al. (2020)	L	L	L	L	L	L
Ahmadi et al. (2020)	U	U	L	L	U	H
Burke et al. (2001)	L	L	L	L	L	L
Rankin et al. (2004)	H	U	L	L	U	H
Samadi et al. (2021)	L	L	L	L	L	L
Teixeira et al. (2022)	L	L	L	L	L	L
Pettersson et al. (2021)	L	L	L	L	L	L
Gryson et al. (2014)	L	L	L	L	L	L
Hulmi et al. (2015)	L	L	L	L	L	L
Maltais et al. (2016)	L	L	L	L	L	L
Keogh & Clifton (2008)	L	L	L	L	L	L
Fernandes et al. (2018)	L	L	L	L	L	L
Rambousková et al. (2014)	H	U	L	L	U	H
Piccolo et al. (2015)	L	L	L	L	L	L
Brown et al. (2004)	L	L	L	L	L	L
Hartman et al. (2007)	H	U	L	L	U	H
Cribb et al. (2007)	L	L	L	L	L	L
Sattler et al. (2008)	L	L	L	L	L	L
Eliot et al. (2008)	L	L	L	L	L	L
Josse et al. (2010)	U	U	L	L	U	H
Mojtahedi et al. (2011)	L	L	L	L	L	L
Arazi et al. (2011)	L	L	L	L	L	L
Baer et al. (2011)	L	L	L	L	L	L
Elahikhah et al. (2024)	U	U	L	L	U	H
Giglio et al. (2019)	L	L	L	L	L	L
DeNysschen et al. (2009)	L	L	L	L	L	L
Haidari et al. (2020)	H	U	L	L	U	H
Hambre et al. (2012)	H	U	L	L	U	H
Ottestad et al. (2017)	L	L	L	L	L	L

Table S2. *Cont.*

Reference	Bias arising from the randomization process (Allocation bias)	Bias due to deviations from the intended interventions (Performance bias)	Bias due to missing outcome data (Attrition bias)	Bias in the measurement of the Outcome (Detection bias)	Bias in the selection of reported results (Reporting bias)	The overall risk of bias
Lopes Gomes et al. (2017)	H	U	L	L	U	H
Sugawara et al. (2012)	L	L	L	L	L	L
Björkman et al. (2012)	H	U	L	L	U	H
Joy et al. (2013)	L	L	L	L	L	L
Herda et al. (2013)	L	L	L	L	L	L
Volek et al. (2013)	L	L	L	L	L	L
Chalé et al. (2013)	L	L	L	L	L	L
Babault et al. (2014)	L	L	L	L	L	L
Duff et al. (2014)	L	L	L	L	L	L
Zhu et al. (2015)	L	L	L	L	L	L
Hulmi et al. (2009)	L	L	L	L	L	L
Kerstetter et al. (2015)	L	L	L	L	L	L
Hector et al. (2015)	L	L	L	L	L	L
Malekian et al. (2015)	H	U	L	L	U	H
Taylor et al. (2016)	L	L	L	L	L	L
Reidy et al. (2016)	L	L	L	L	L	L
Naclerio et al. (2017)	L	L	L	L	L	L
Naclerio et al. (2017)	L	L	L	L	L	L
Stojkovic et al. (2017)	L	L	L	L	L	L
Hwang et al. (2017)	L	L	L	L	L	L
Dudgeon et al. (2017)	U	U	L	L	U	H
Mobley et al. (2017)	L	L	L	L	L	L
Reimer et al. (2017)	L	L	L	L	L	L
Hassan & Hassan (2017)	H	U	L	L	U	H
Hassan (2017)	H	U	L	L	U	H
Dirks et al. (2017)	H	U	L	L	U	H
Gjevestad et al. (2017)	L	L	L	L	L	L
Mori et al. (2018)	U	U	L	L	U	H
Gaffney et al. (2018)	L	L	L	L	L	L
Holwerda et al. (2018)	L	L	L	L	L	L
McAdam et al. (2018)	L	L	L	L	L	L
Englund et al. (2018)	L	L	L	L	L	L
Sahathevan et al. (2018)	H	U	L	L	U	H
Park et al. (2019)	L	L	L	L	L	L
Forbes et al. (2019)	L	L	L	L	L	L

Table S2. *Cont.*

Reference	Bias arising from the randomization process (Allocation bias)	Bias due to deviations from the intended interventions (Performance bias)	Bias due to missing outcome data (Attrition bias)	Bias in the measurement of the Outcome (Detection bias)	Bias in the selection of reported results (Reporting bias)	The overall risk of bias
Amasene et al. (2019)	L	L	L	L	L	L
Cereda et al. (2019)	U	U	L	L	U	H
Ten Haaf et al. (2019)	L	L	L	L	L	L
Kang et al. (2019)	H	U	L	L	U	H
Rakvaag et al. (2019)	L	L	L	L	L	L
Brown et al. (2020)	L	L	L	L	L	L
McAdam et al. (2022)	L	L	L	L	L	L
Obradović et al. (2020)	L	L	L	L	L	L
Lynch et al. (2020)	L	L	L	L	L	L
Boutry-Regard et al. (2020)	L	L	L	L	L	L
Mori et al. (2021)	H	U	L	L	U	H
Biesek et al. (2021)	U	U	L	L	U	H
Dulac et al. (2021)	L	L	L	L	L	L
Roberson et al. (2021)	L	L	L	L	L	L
Nakayama et al. (2021)	L	L	L	L	L	L
Koopmans et al. (2024)	L	L	L	L	L	L
Azhar et al. (2021)	L	L	L	L	L	L
Li et al. (2021)	H	U	L	L	U	H
Mizubuti et al. (2021)	L	L	L	L	L	L
Mertz et al. (2021)	L	L	L	L	L	L
Bach et al. (2022)	L	L	L	L	L	L
Henriques et al. (2023)	L	L	L	L	L	L
Zbinden-Foncea et al. (2023)	U	U	L	L	U	H
Yapici et al. (2023)	H	U	L	L	U	H
Kim et al. (2023)	L	L	L	L	L	L
Zong et al. (2023)	H	U	L	L	U	H
Nouri et al. (2024)	L	L	L	L	L	L
Furtado et al. (2024)	L	L	L	L	L	L
Kemmler et al. (2018)	H	U	L	L	U	H
Kirk et al. (2021)	H	U	L	L	U	H
Santos et al. (2023)	U	U	L	L	U	H
Kasim-Karakas et al. (2009)	U	U	L	L	U	H
Lockwood et al. (2017)	L	L	L	L	L	L
Knuiman et al.2019	L	L	L	L	L	L
Mhamed et al.2024	H	U	L	L	U	H
Bodaghabadi et al.2023	H	U	L	L	U	H
Soares et al.2023	L	L	L	L	L	L
Ferguson-Stegall et al.2011	H	U	L	L	U	H

Table S2. *Cont.*

**Reference**	Bias arising from the randomization process (Allocation bias)	Bias due to deviations from the intended interventions (Performance bias)	Bias due to missing outcome data (Attrition bias)	Bias in the measurement of the Outcome (Detection bias)	Bias in the selection of reported results (Reporting bias)	The overall risk of bias
Wilborn et al.2013	L	L	L	L	L	L
Reljic et al.2022	L	L	L	L	L	L
Reljic et al.2024	L	L	L	L	L	L
Murray et al.2025	L	L	L	L	L	L
Yıldız et al.2025	H	U	L	L	U	H
Sabooni et al.2025	L	L	L	L	L	L
Ormsbee et al.2018	H	U	L	L	U	H
Jonvik et al.2019	L	L	L	L	L	L
Griffen et al. 2022	L	L	L	L	L	L
Arnarson et al. 2013	L	L	L	L	L	L
Karelis et al. 2015	L	L	L	L	L	L
Kirk et al. 2019	H	U	L	L	U	H
Michel et al. 2022	H	U	L	L	U	H

Abbreviations: L, low risk of bias; H, high risk of bias; U, unclear risk of bias (some concerns)

Table S3. GRADE assessment

Outcomes	Risk of bias	Inconsistency	Indirectness	Imprecision	Publication Bias	Quality of evidence
BW	No Serious limitation	No Serious limitation	No serious limitation	No serious limitation	No serious limitation	⊕⊕⊕⊕ High
BMI	No Serious limitation	No Serious limitation	No serious limitation	No serious limitation	No serious limitation	⊕⊕⊕⊕ High
WC	No Serious limitation	No Serious limitation	No serious limitation	No serious limitation	No serious limitation	⊕⊕⊕⊕ High
FM	No Serious limitation	No Serious limitation	No serious limitation	No serious limitation	No serious limitation	⊕⊕⊕⊕ High
BFP	No serious limitation	Serious limitation[1]	No serious limitation	No serious limitation	No serious limitation	⊕⊕⊕⊖ Moderate
FFM	No serious limitation	No Serious limitation	No serious limitation	No serious limitation	No serious limitation	⊕⊕⊕⊕ High
LBM	No serious limitation	No Serious limitation	No serious limitation	No serious limitation	No serious limitation	⊕⊕⊕⊕ High
MM	No serious limitation	No Serious limitation	No serious limitation	No serious limitation	No serious limitation	⊕⊕⊕⊕ High

Abbreviations: BMI, body mass index; WC, waist circumference; FFM, fat-free mass; FM, fat mass; LBM, lean body mass; BFP, body fat percentage; BW, body weight; FM, fat-free mass; LBM, lean body mass.

[1]It was downgraded due to the presence of significant heterogeneity (I^2>50%).

A) BW

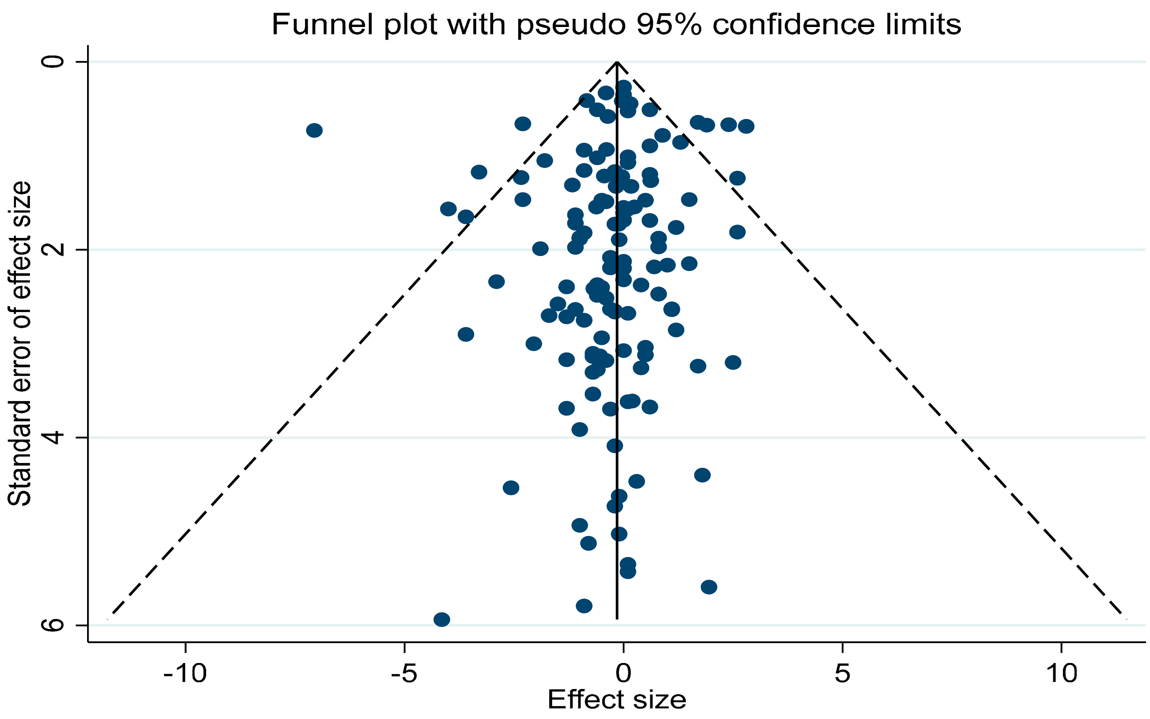
Funnel plot with pseudo 95% confidence limits
Standard error of effect size
0
2
4
6
-10
-5
0
5
10
Effect size

B) BMI

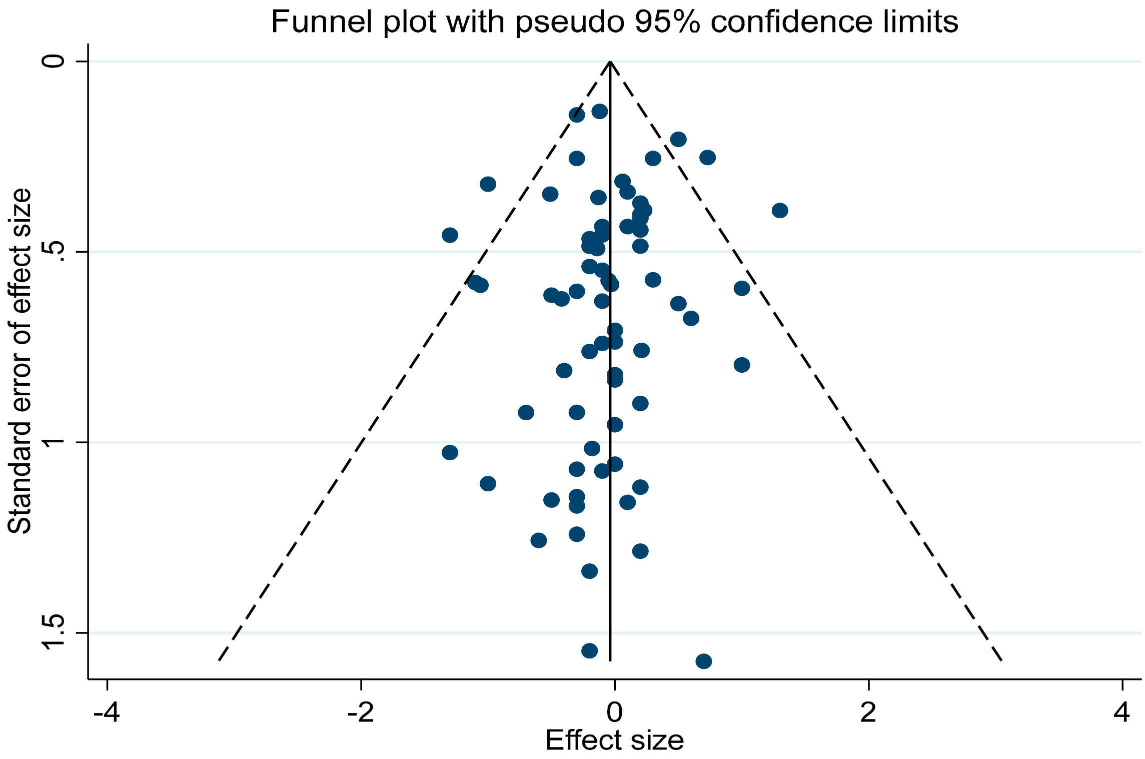
Funnel plot with pseudo 95% confidence limits
Standard error of effect size
0
.5
1
1.5
-4
-2
0
2
4
Effect size

C) WC

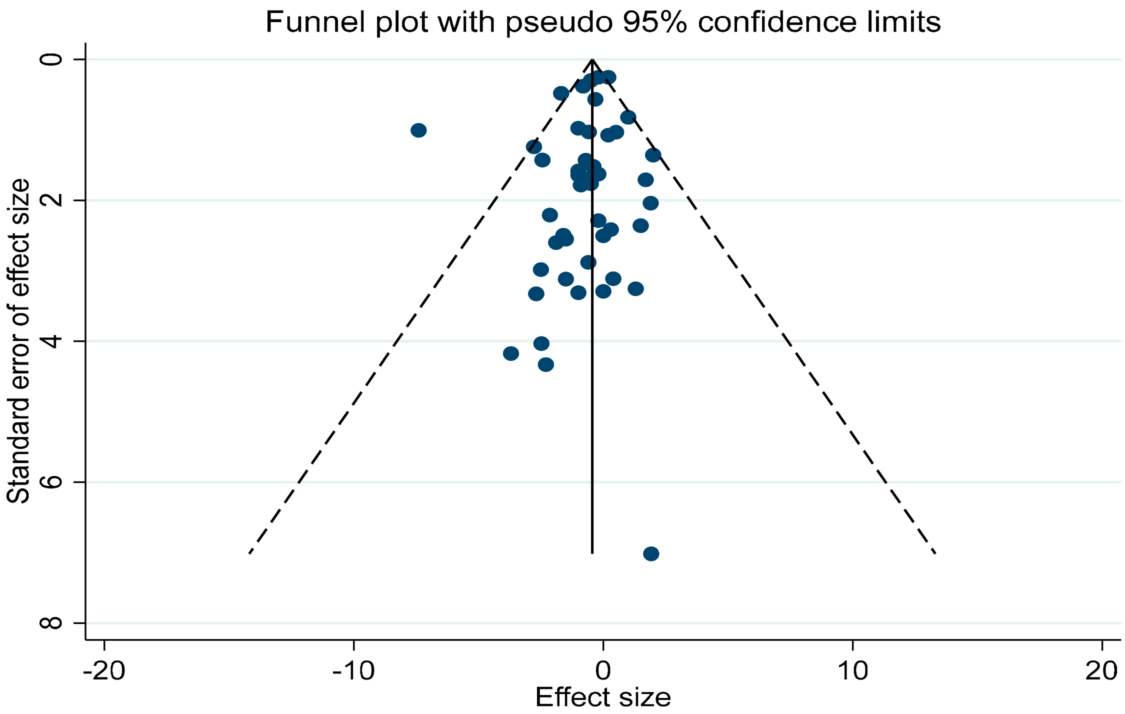
Funnel plot with pseudo 95% confidence limits
Standard error of effect size
0
2
4
6
8
-20
-10
0
10
20
Effect size

D) FM

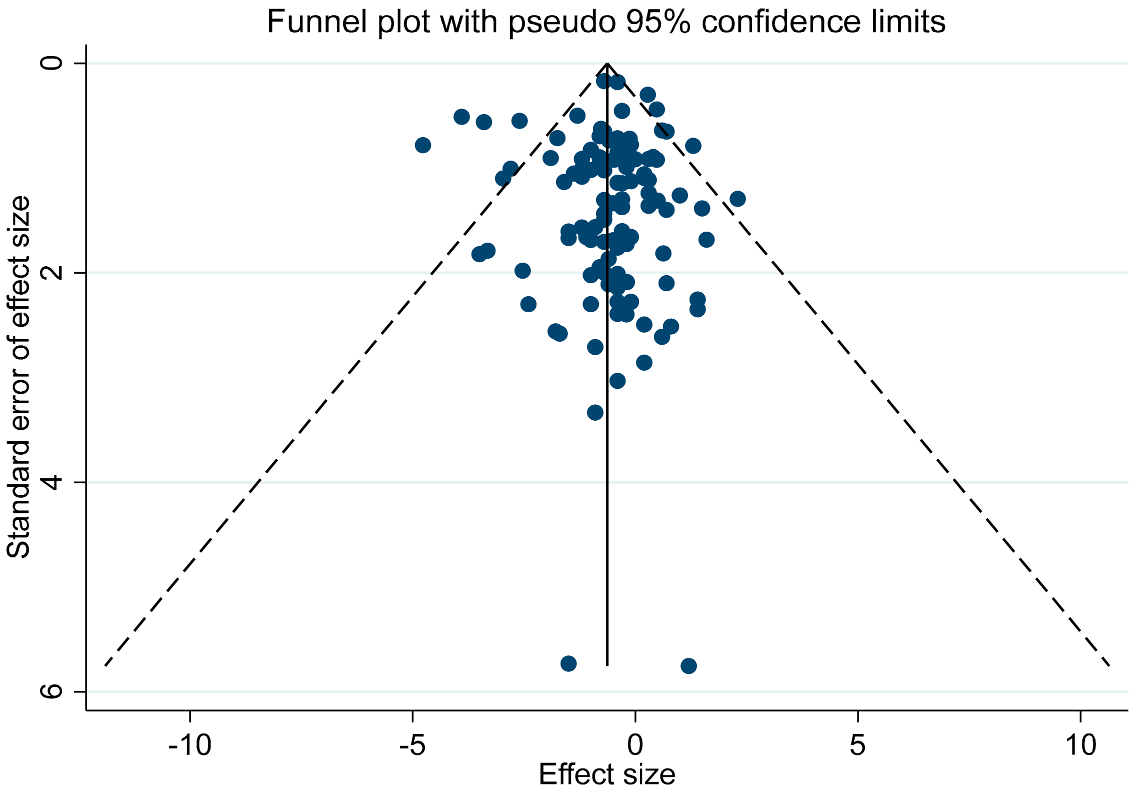
Funnel plot with pseudo 95% confidence limits
Standard error of effect size
0
2
4
6
-10
-5
0
5
10
Effect size

E) BFP

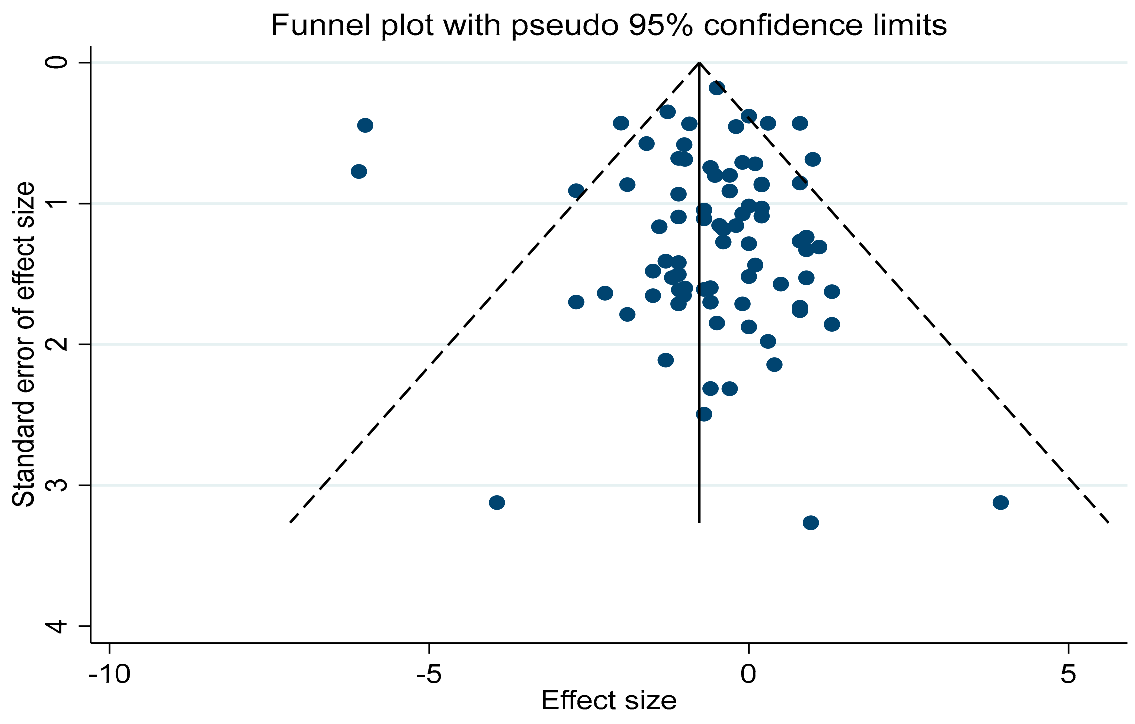
Funnel plot with pseudo 95% confidence limits
Standard error of effect size
0
1
2
3
4
-10
-5
0
5
Effect size

F) FFM

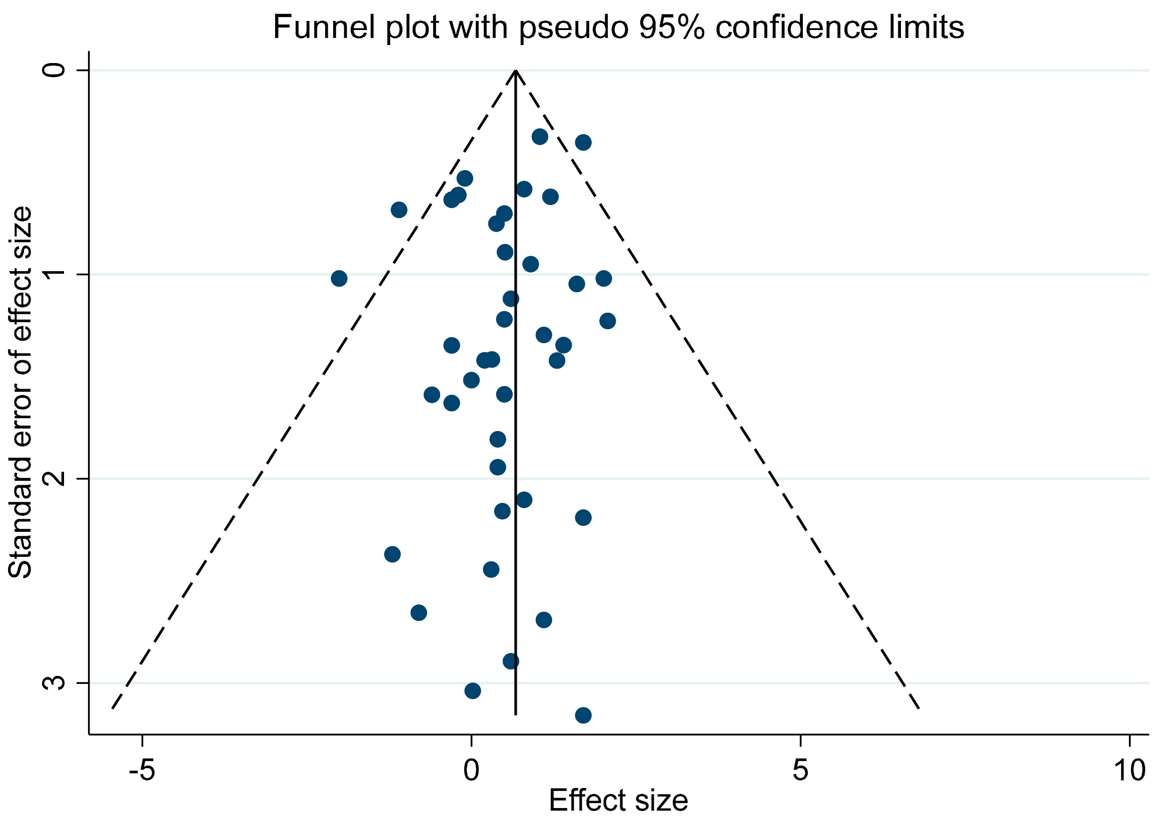
Funnel plot with pseudo 95% confidence limits
Standard error of effect size
0
1
2
3
-5
0
5
10
Effect size

G) LBM

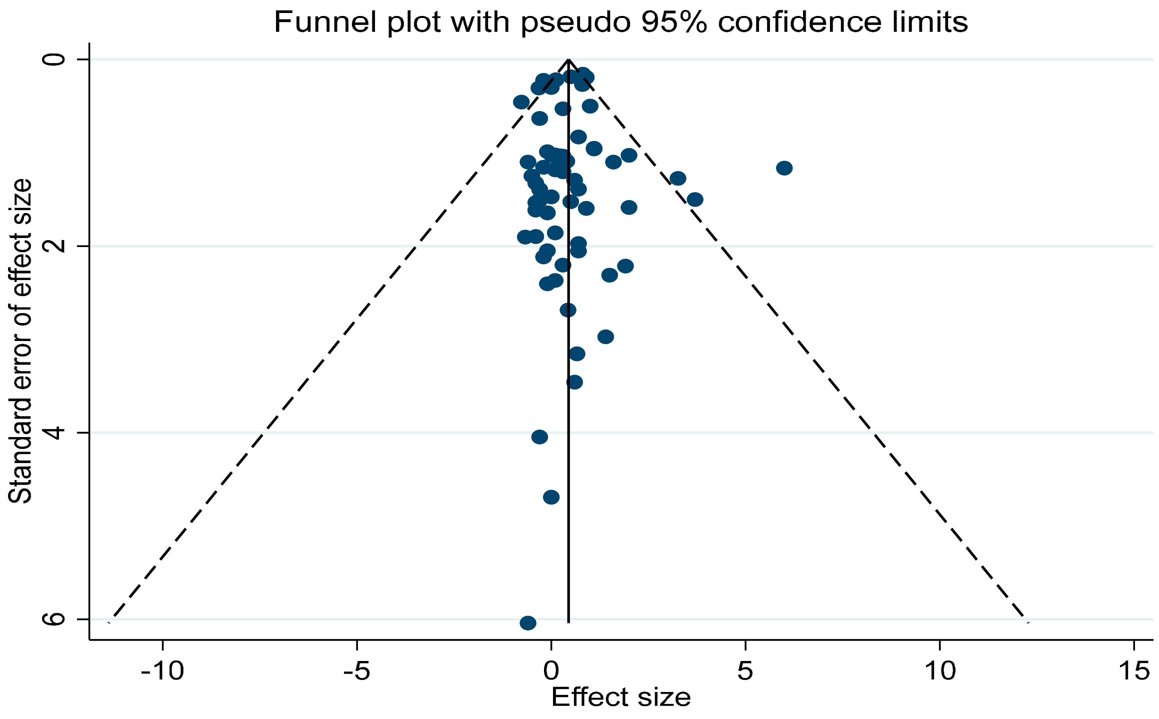

H) MM

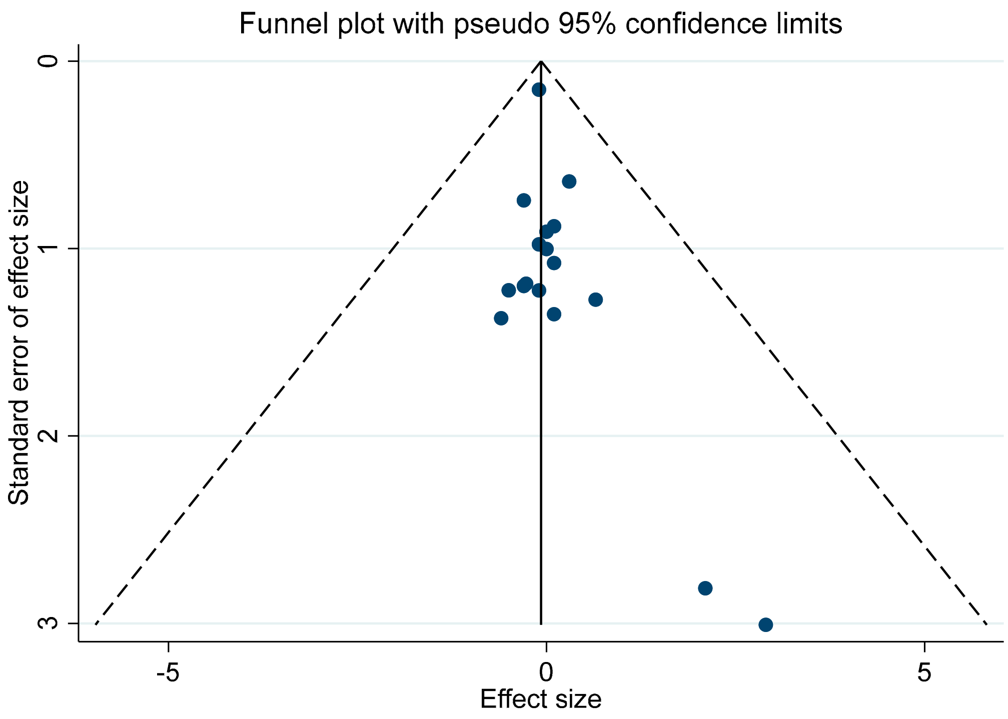

Figure S1. Funnel plots for the effects of milk protein supplementation on **(A)** BW, **(B)** BMI, **(C)** WC, **(D)** FM, **(E)** BFP, **(F)** FFM, **(G)** LBM, and **(H)** MM.

A) BW

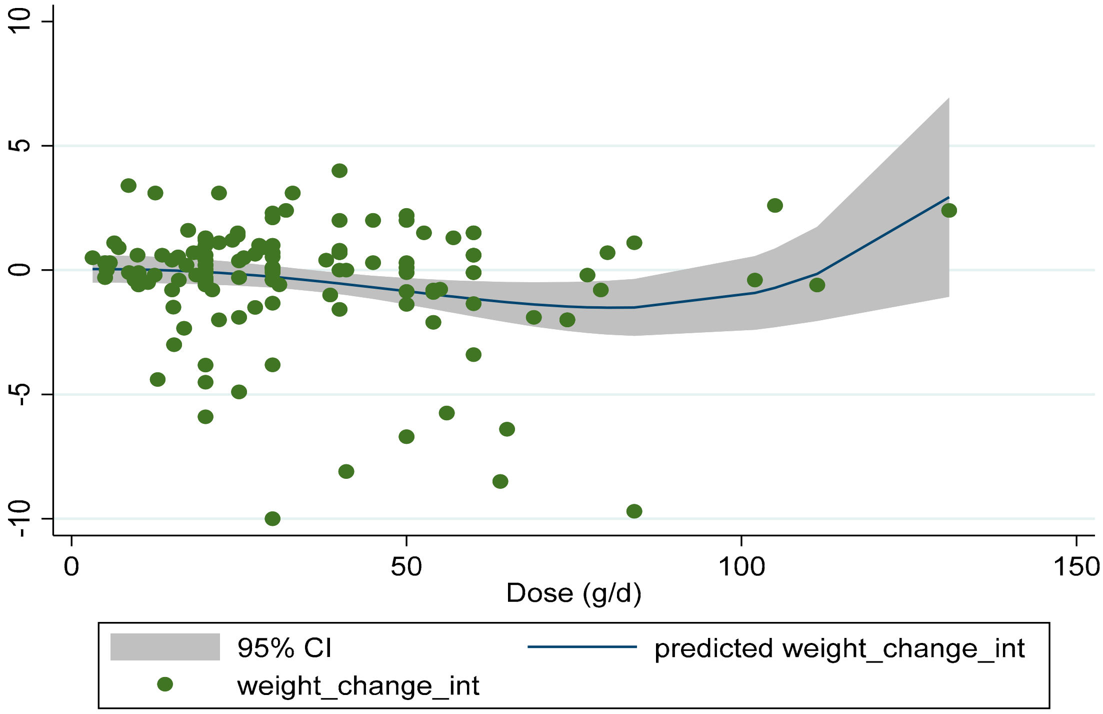
10
5
0
-5
-10
0
50
100
150
Dose (g/d)
95% CI
predicted weight_change_int
weight_change_int

B) BMI

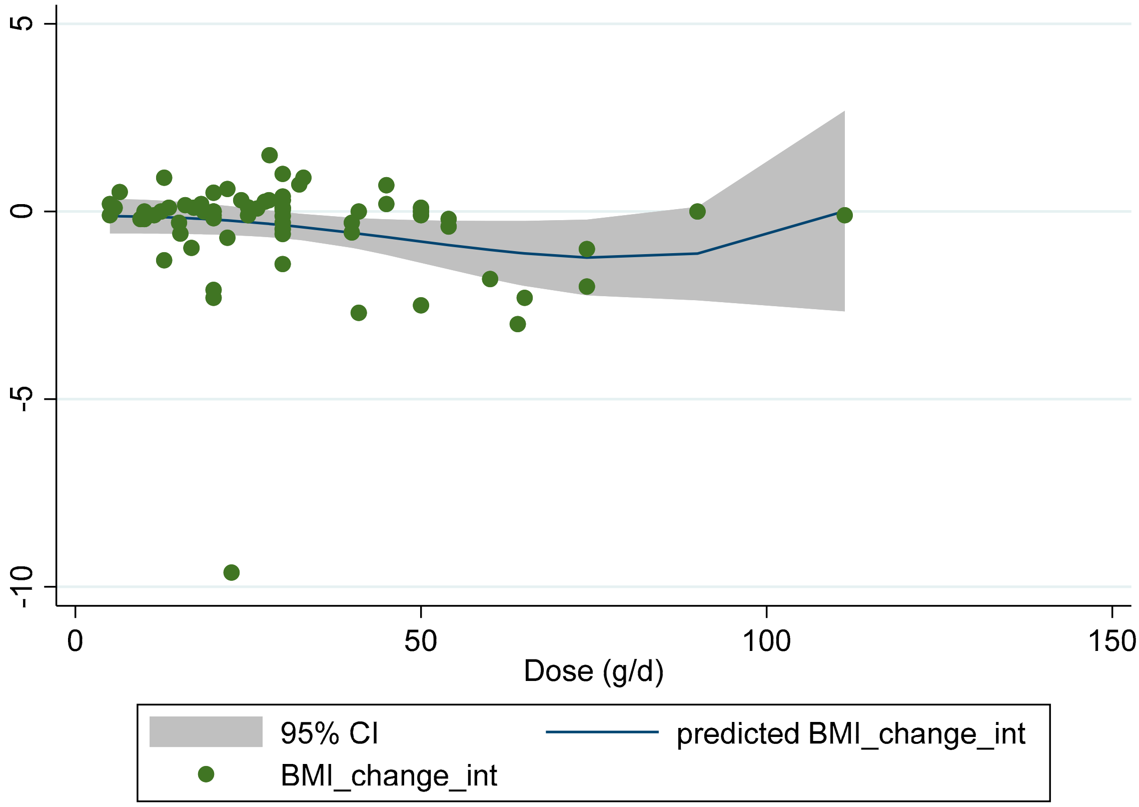
5
0
-5
-10
0
50
100
150
Dose (g/d)
95% CI
predicted BMI_change_int
BMI_change_int

C) WC

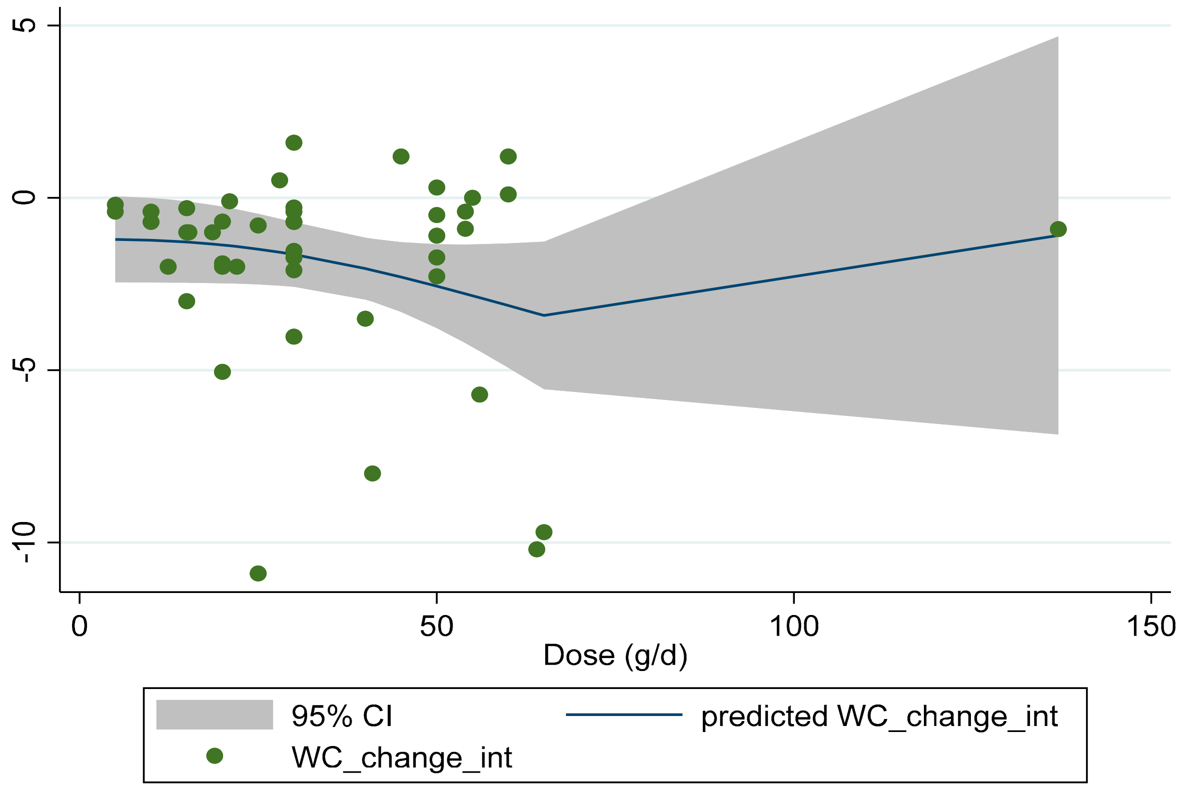
5
0
-5
-10
0
50
100
150
Dose (g/d)
95% CI
predicted WC_change_int
WC_change_int

D) FM

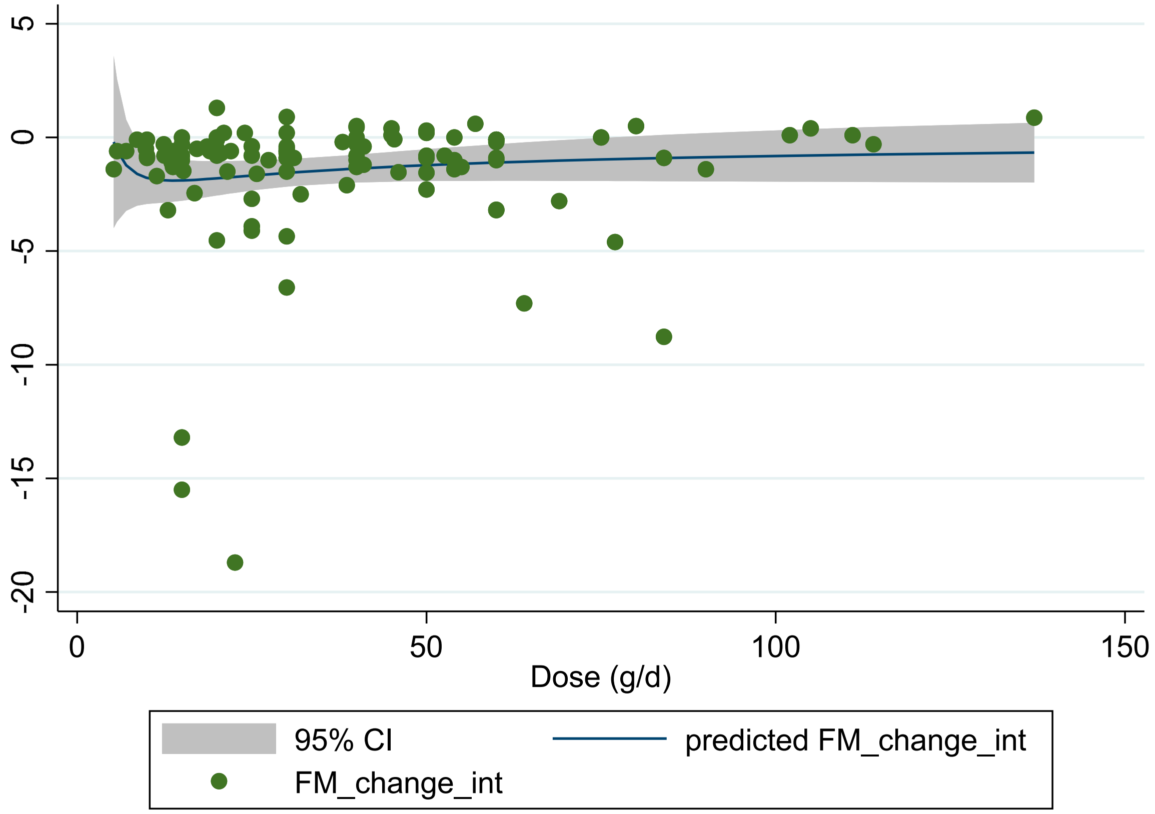
5
0
-5
-10
-15
-20
0
50
100
150
Dose (g/d)
95% CI
predicted FM_change_int
FM_change_int

E) **BFP**

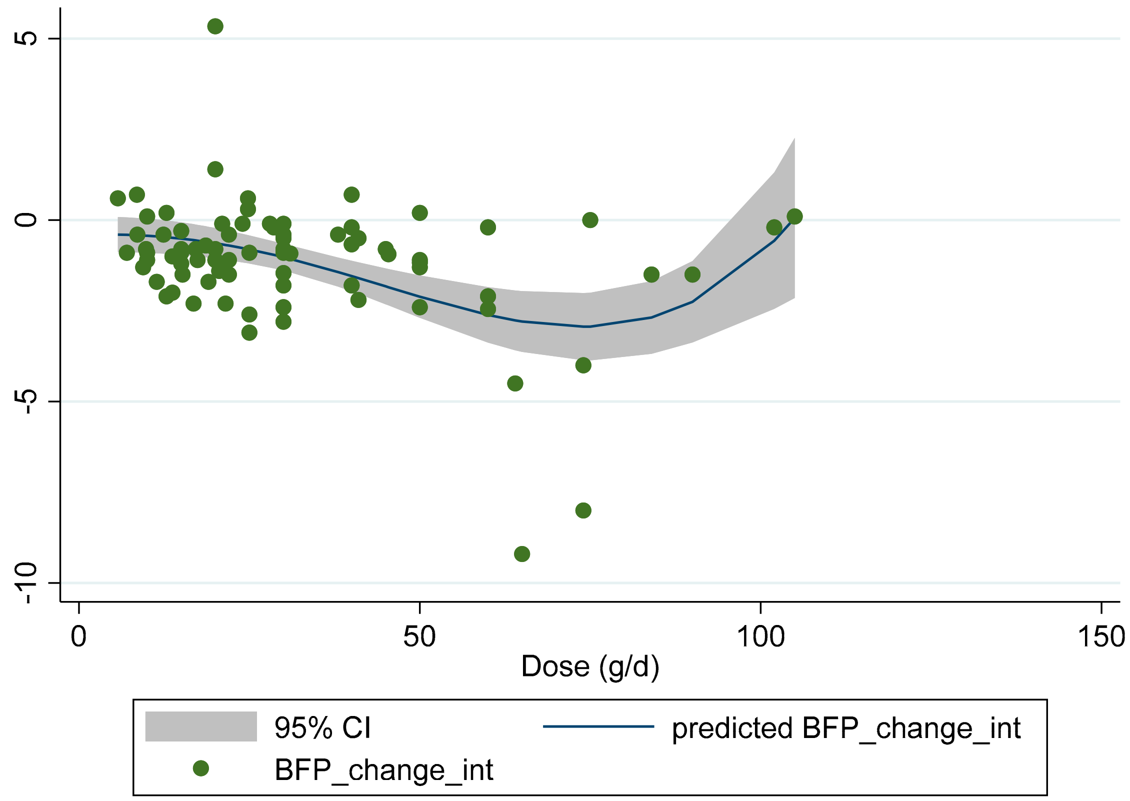
5
0
-5
-10
0
50
100
150
Dose (g/d)
95% CI
predicted BFP_change_int
BFP_change_int

F) **FFM**

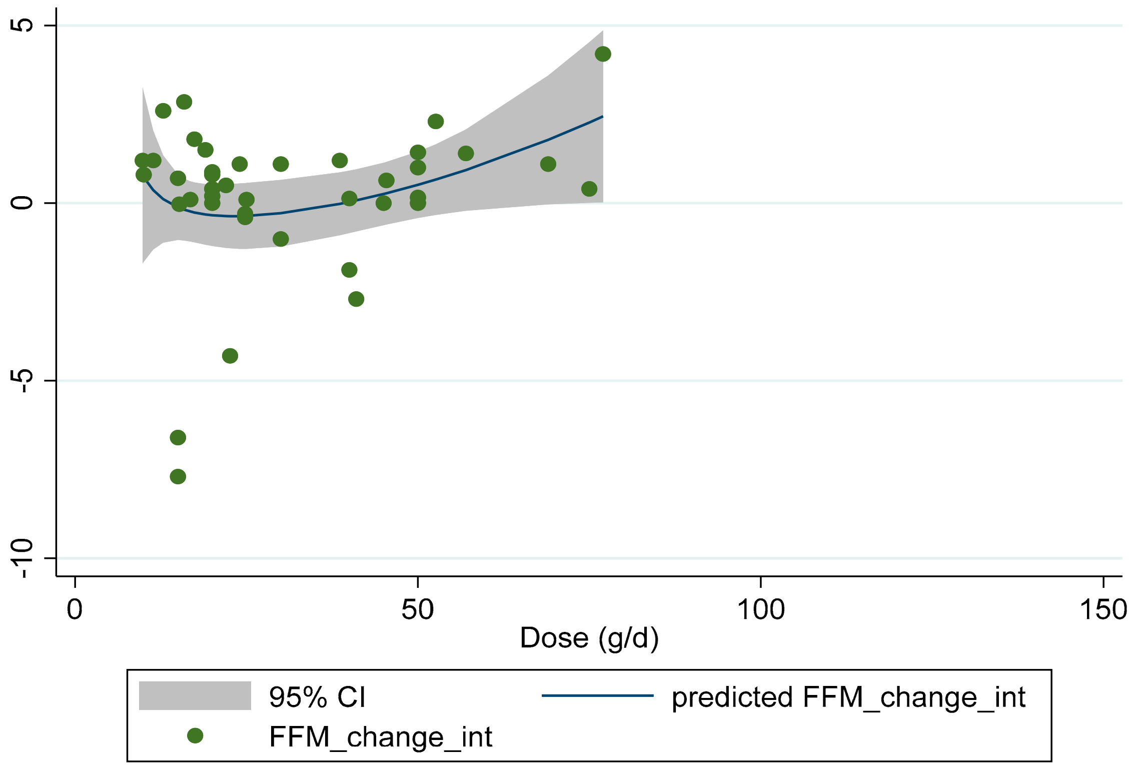
5
0
-5
-10
0
50
100
150
Dose (g/d)
95% CI
predicted FFM_change_int
FFM_change_int

G) LBM

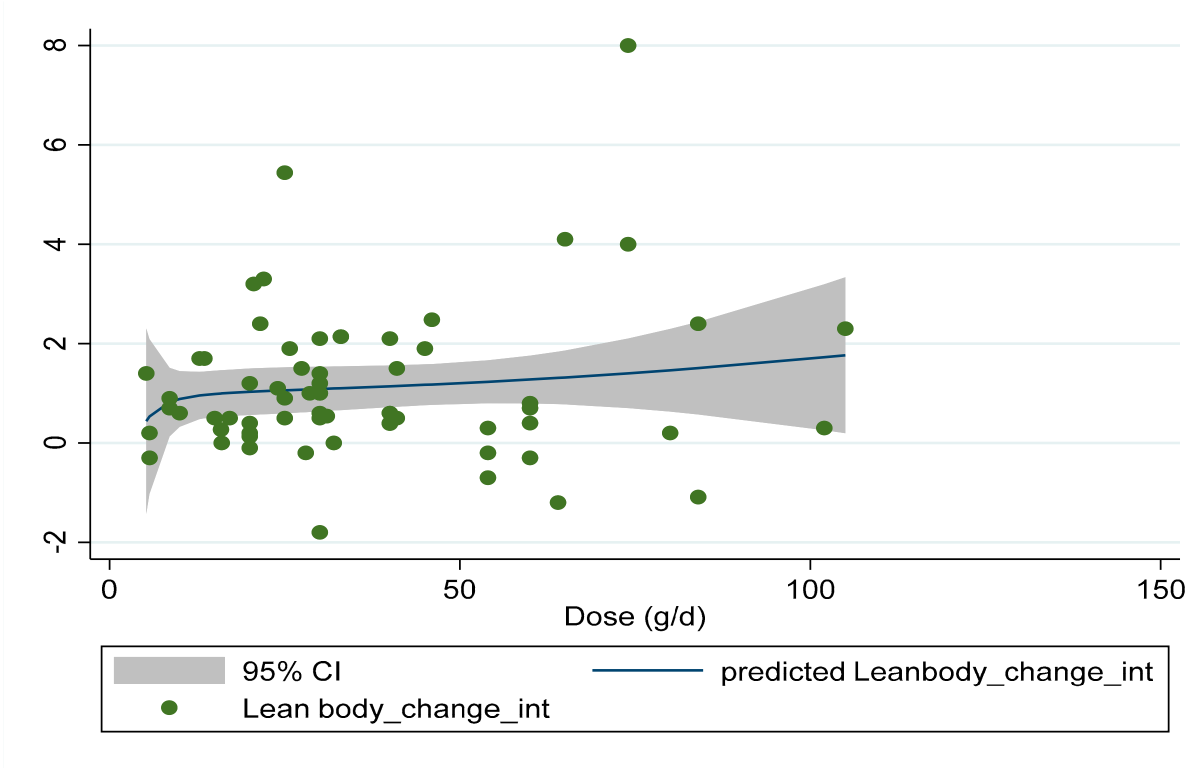

(H) MM

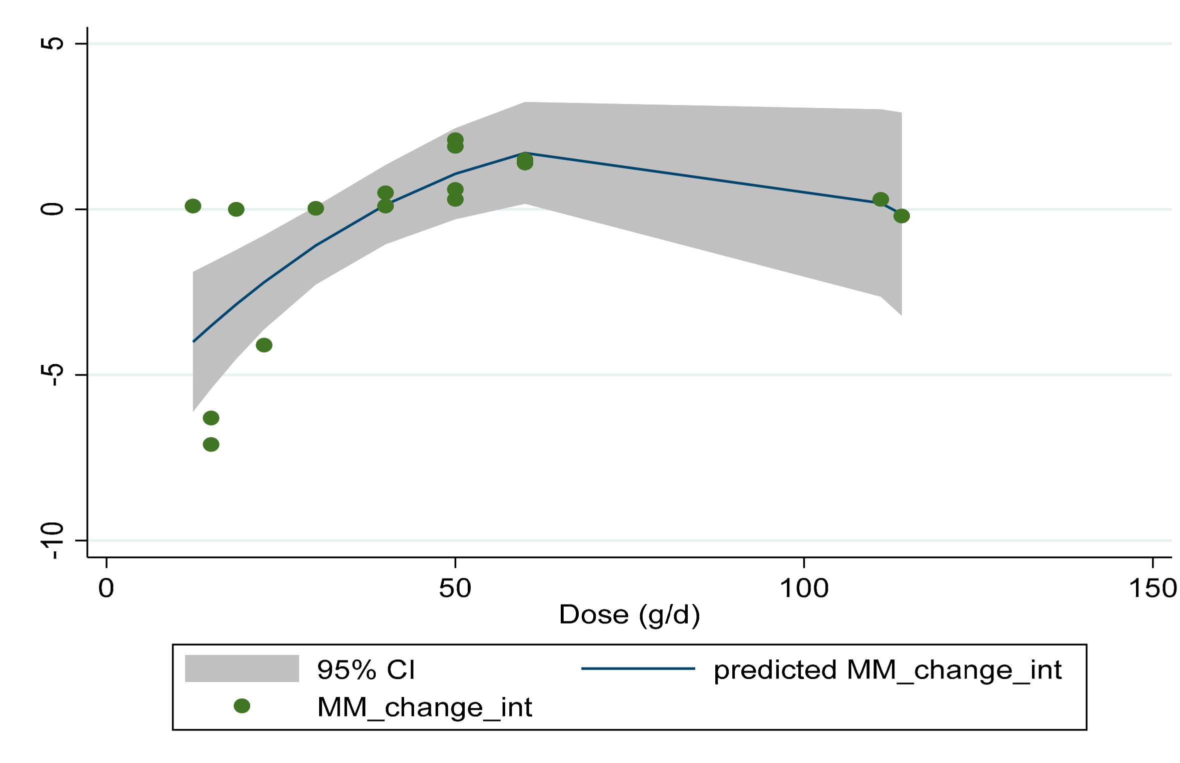

Figure S2. Non-linear dose-response association between dose (g/day) of supplementation with milk protein and absolute mean differences in **(A)** BW (Kg), **(B)** BMI (kg/m²), **(C)** WC (cm), **(D)** FM (kg), **(E)** BFP (%), **(F)** FFM (Kg), **(G)** LBM (kg), and **(H)** MM (kg). The 95% CI is depicted in the shaded parts.

A) BW

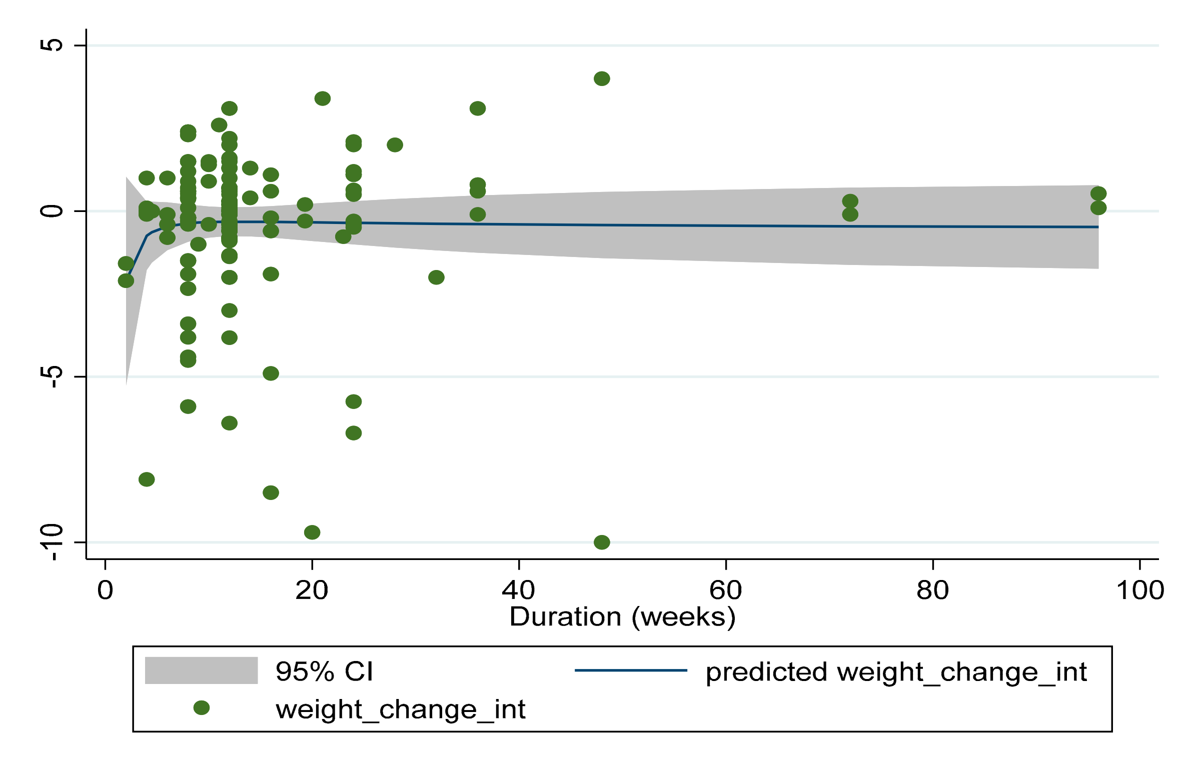
5
0
-5
-10
0
20
40
60
80
100
Duration (weeks)
95% CI
predicted weight_change_int
weight_change_int

B) BMI

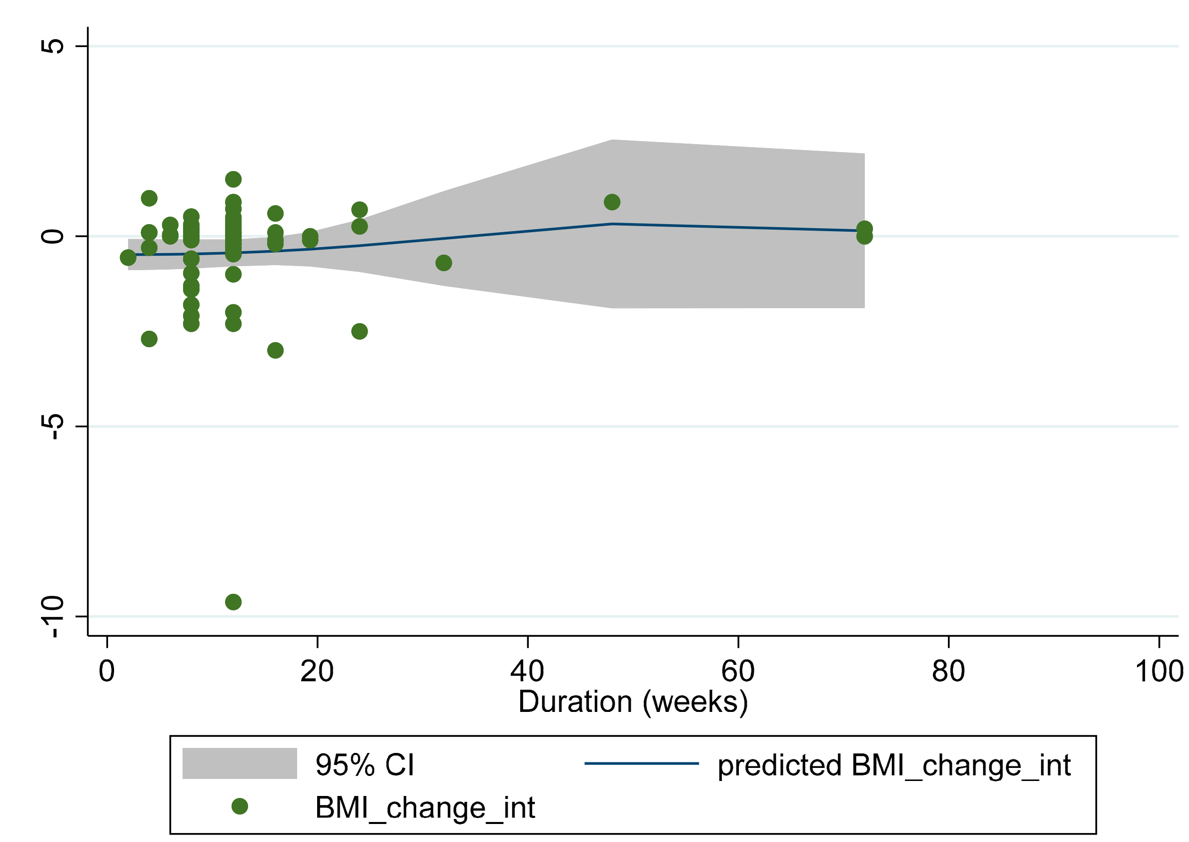
5
0
-5
-10
0
20
40
60
80
100
Duration (weeks)
95% CI
predicted BMI_change_int
BMI_change_int

C) WC

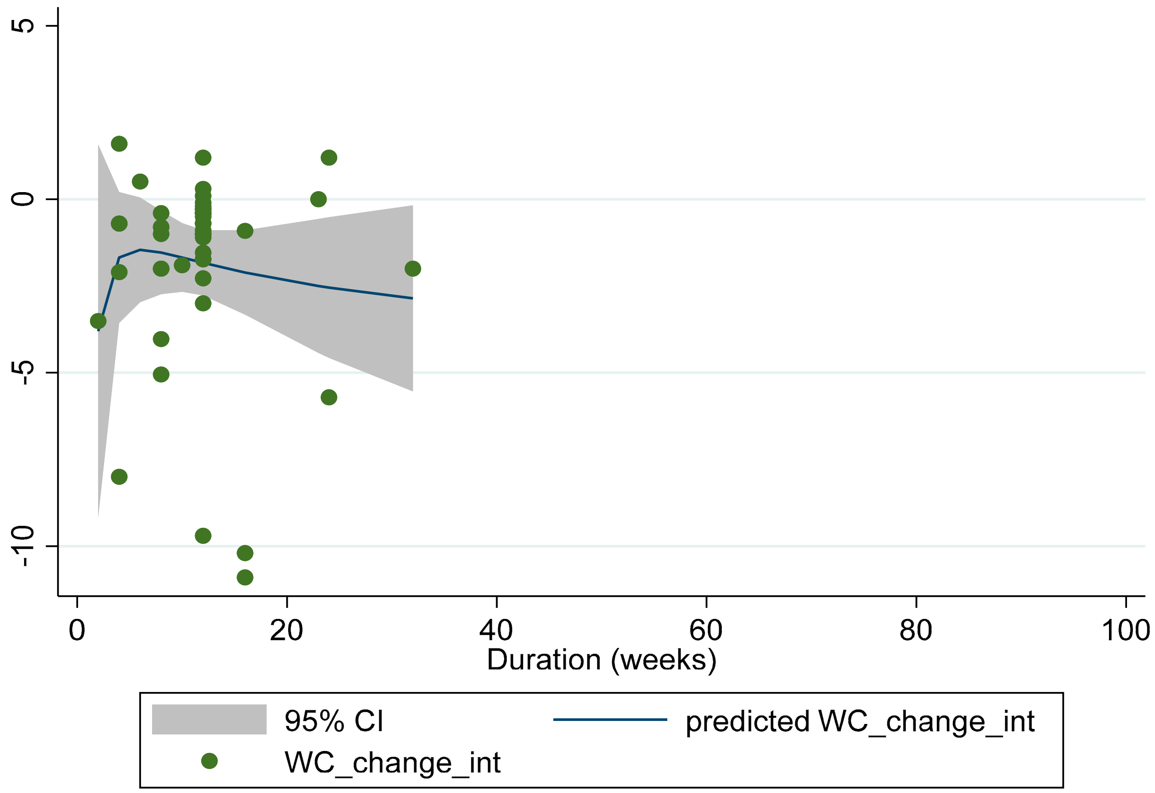
5
0
-5
-10
0
20
40
60
80
100
Duration (weeks)
95% CI
predicted WC_change_int
WC_change_int

D) FM

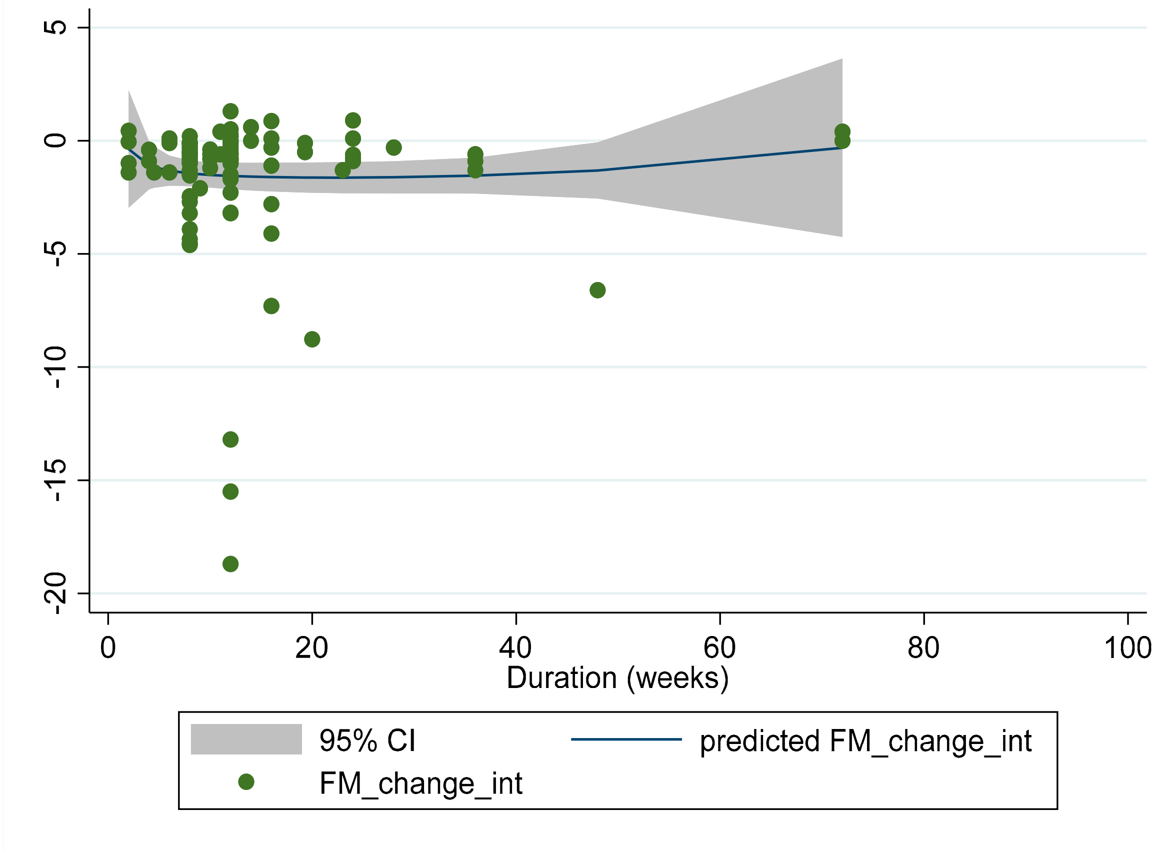
5
0
-5
-10
-15
-20
0
20
40
60
80
100
Duration (weeks)
95% CI
predicted FM_change_int
FM_change_int

E) **BFP**

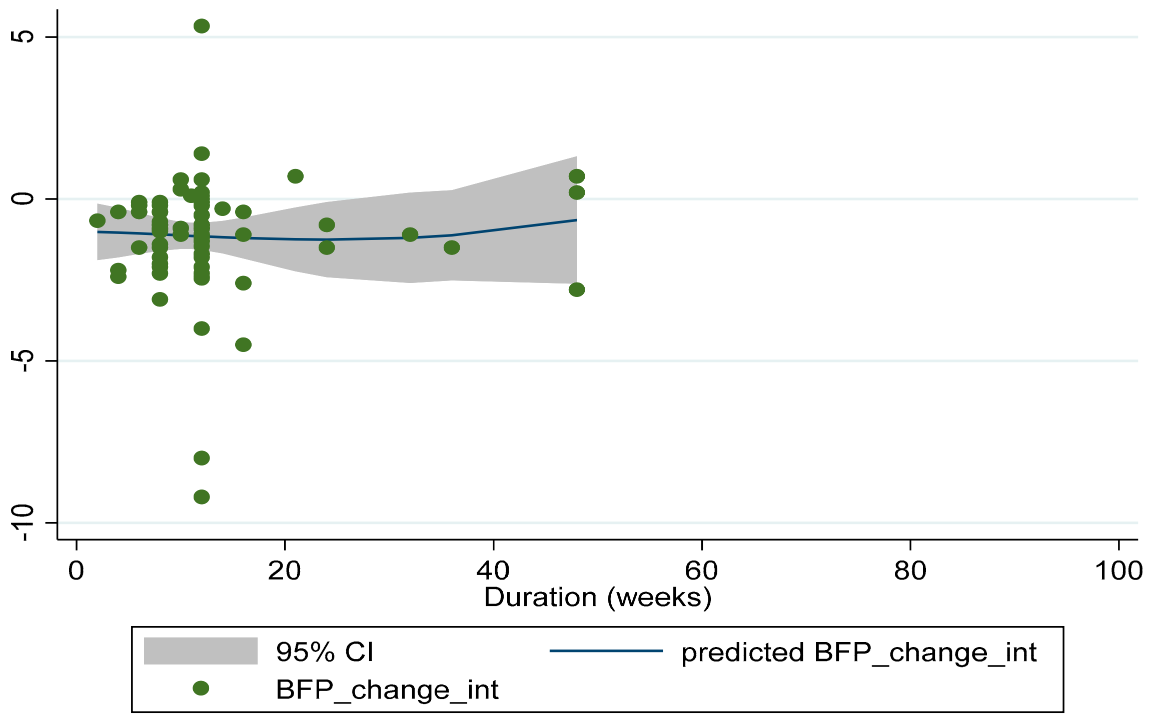
5
0
-5
-10
0
20
40
60
80
100
Duration (weeks)
95% CI
predicted BFP_change_int
BFP_change_int

F) **FFM**

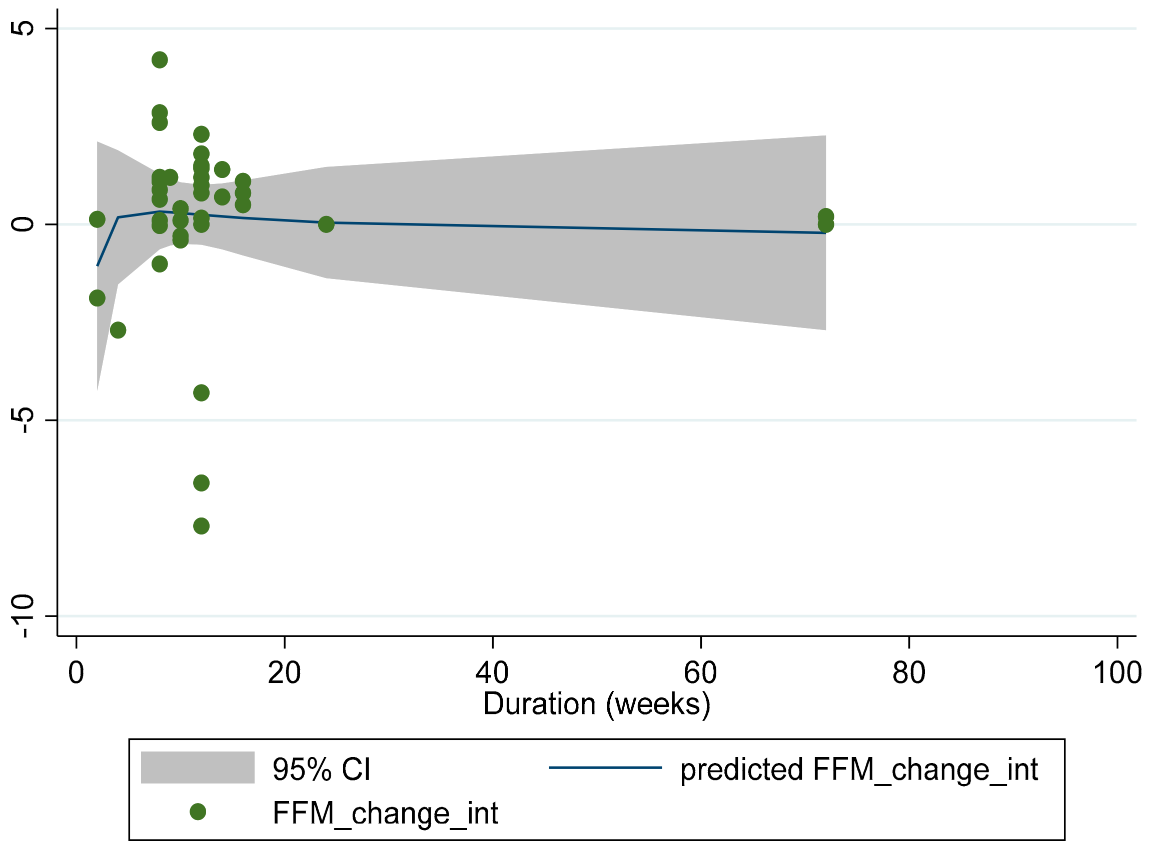
5
0
-5
-10
0
20
40
60
80
100
Duration (weeks)
95% CI
predicted FFM_change_int
FFM_change_int

G) LBM

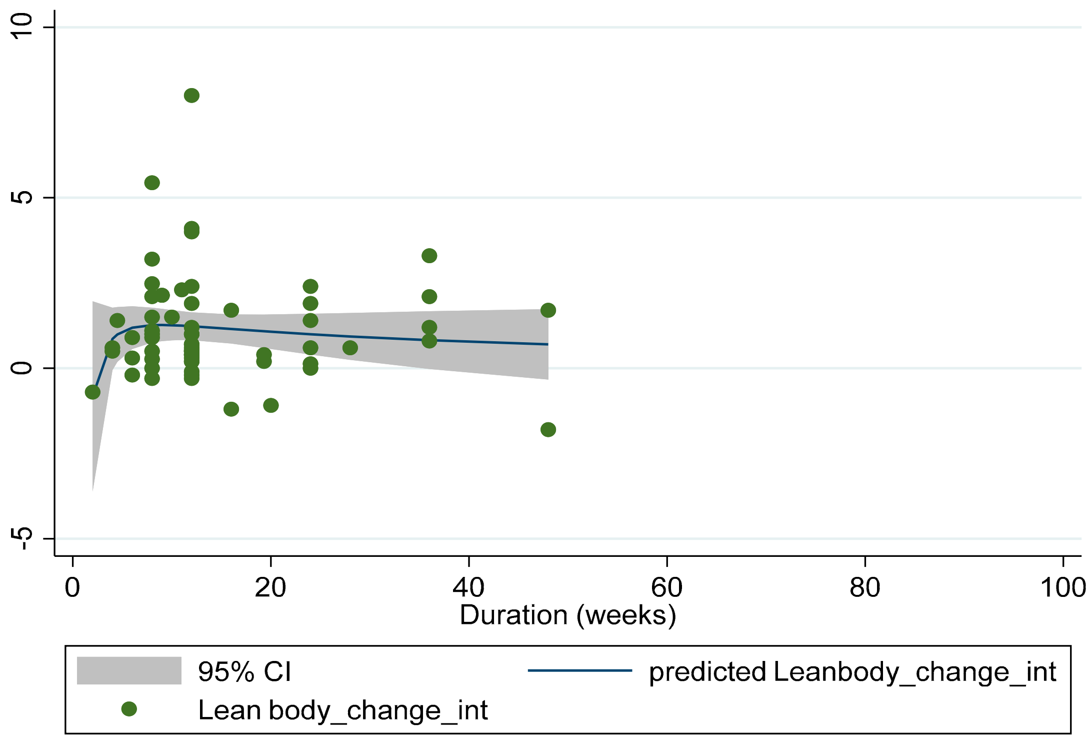

(H) MM

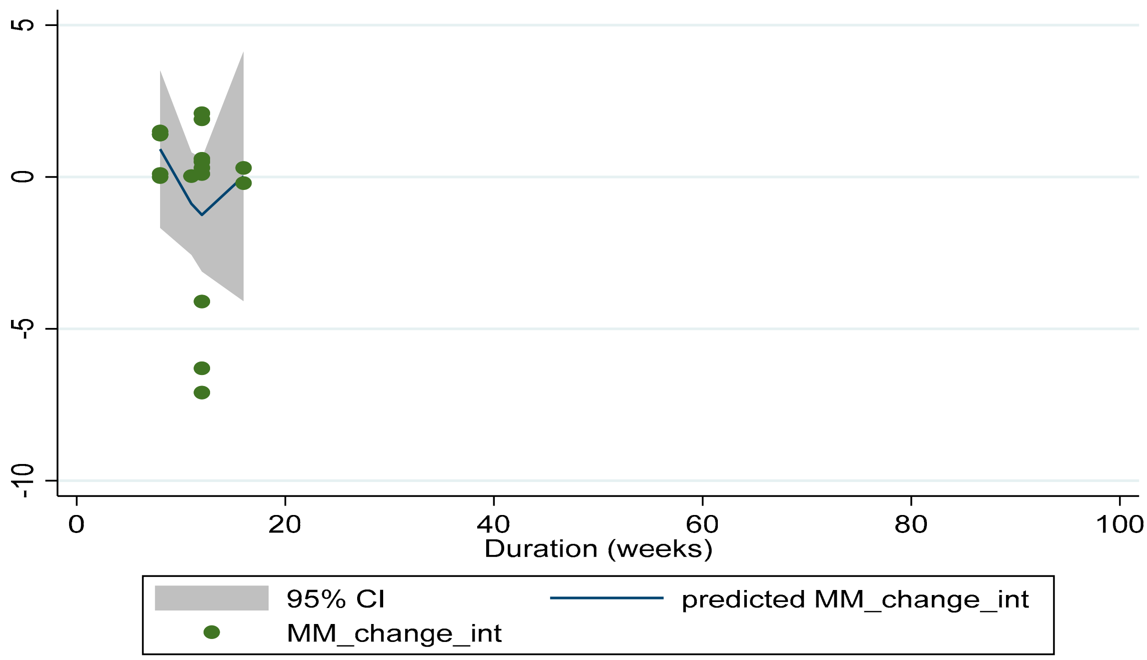

Figure S3. Non-linear dose-response association between the duration of supplementation with milk protein (weeks) and absolute mean differences in **(A)** BW (Kg), **(B)** BMI (kg/m²), **(C)** WC (cm), **(D)** FM (kg), **(E)** BFP (%), **(F)** FFM(Kg), **(G)** LBM (kg), and **(H)** MM (kg). The 95% CI is depicted in the shaded parts.

A) BW

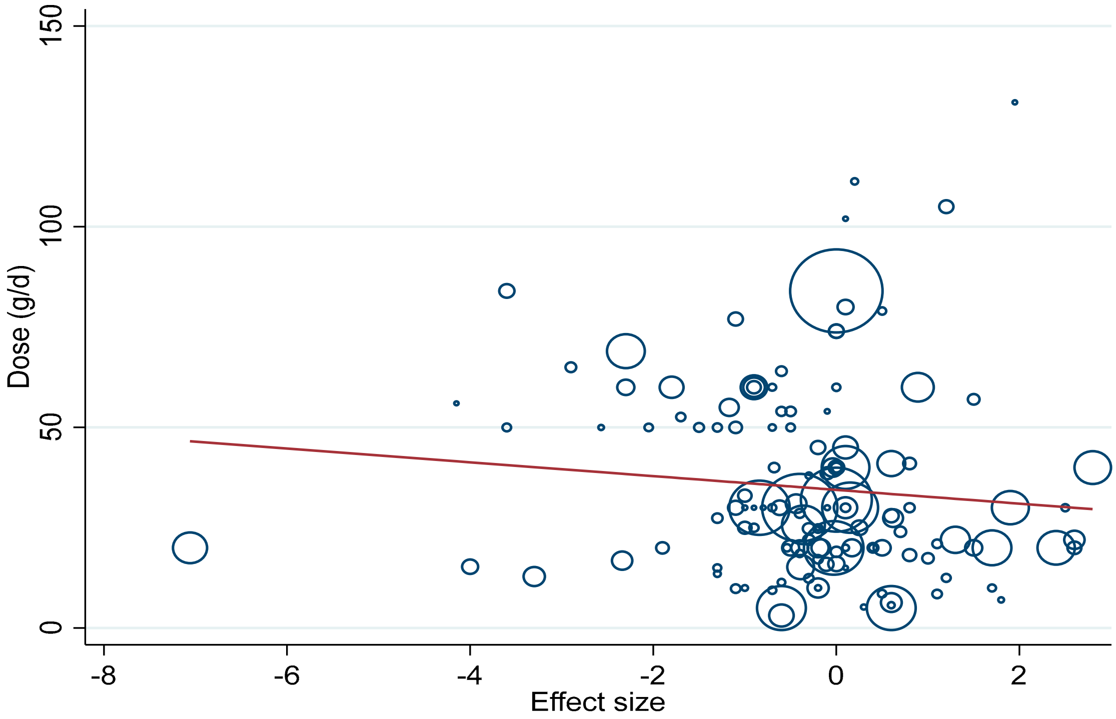
Dose (g/d)
150
100
50
0
-8
-6
-4
-2
0
2
Effect size

B) BMI

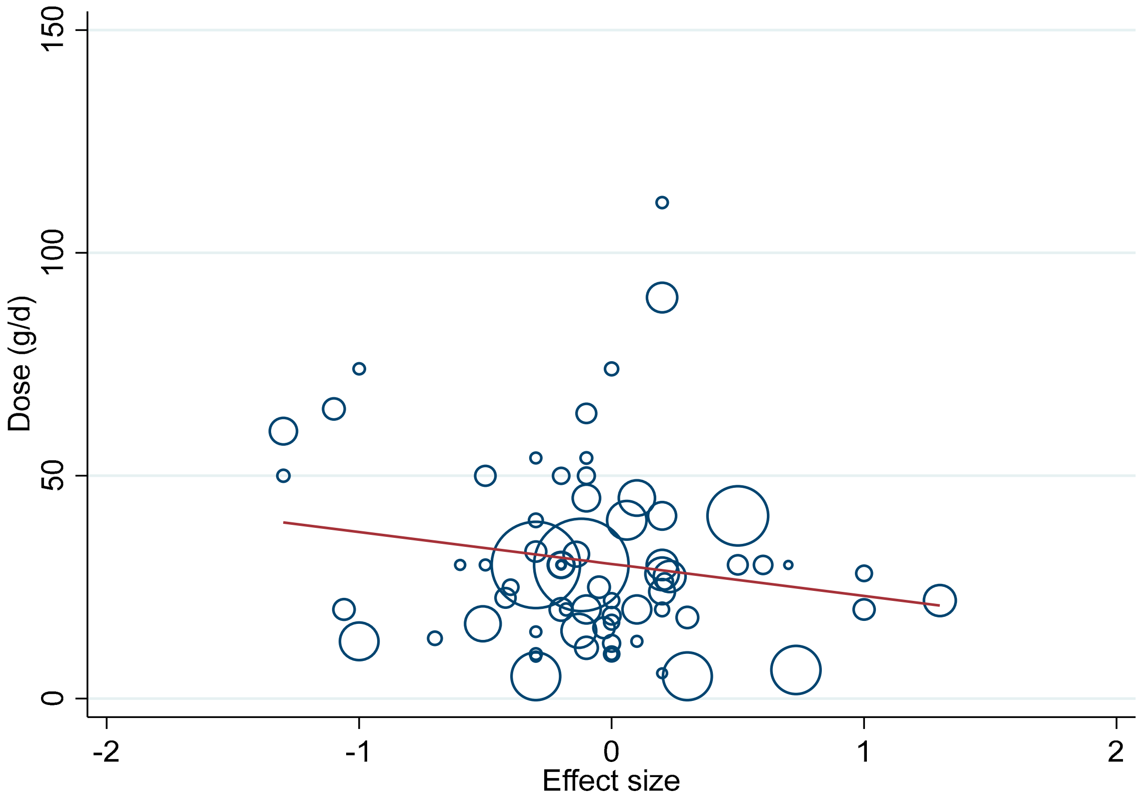
Dose (g/d)
150
100
50
0
-2
-1
0
1
2
Effect size

C) WC

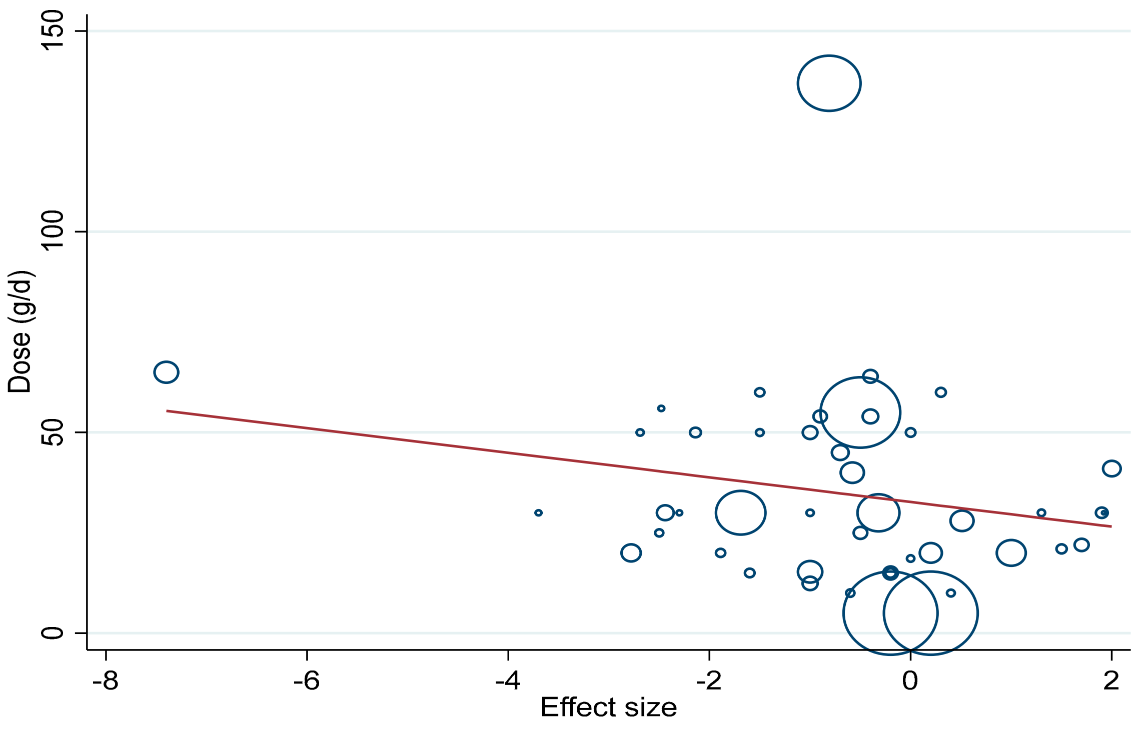
Dose (g/d)
150
100
50
0
-8
-6
-4
-2
0
2
Effect size

D) FM

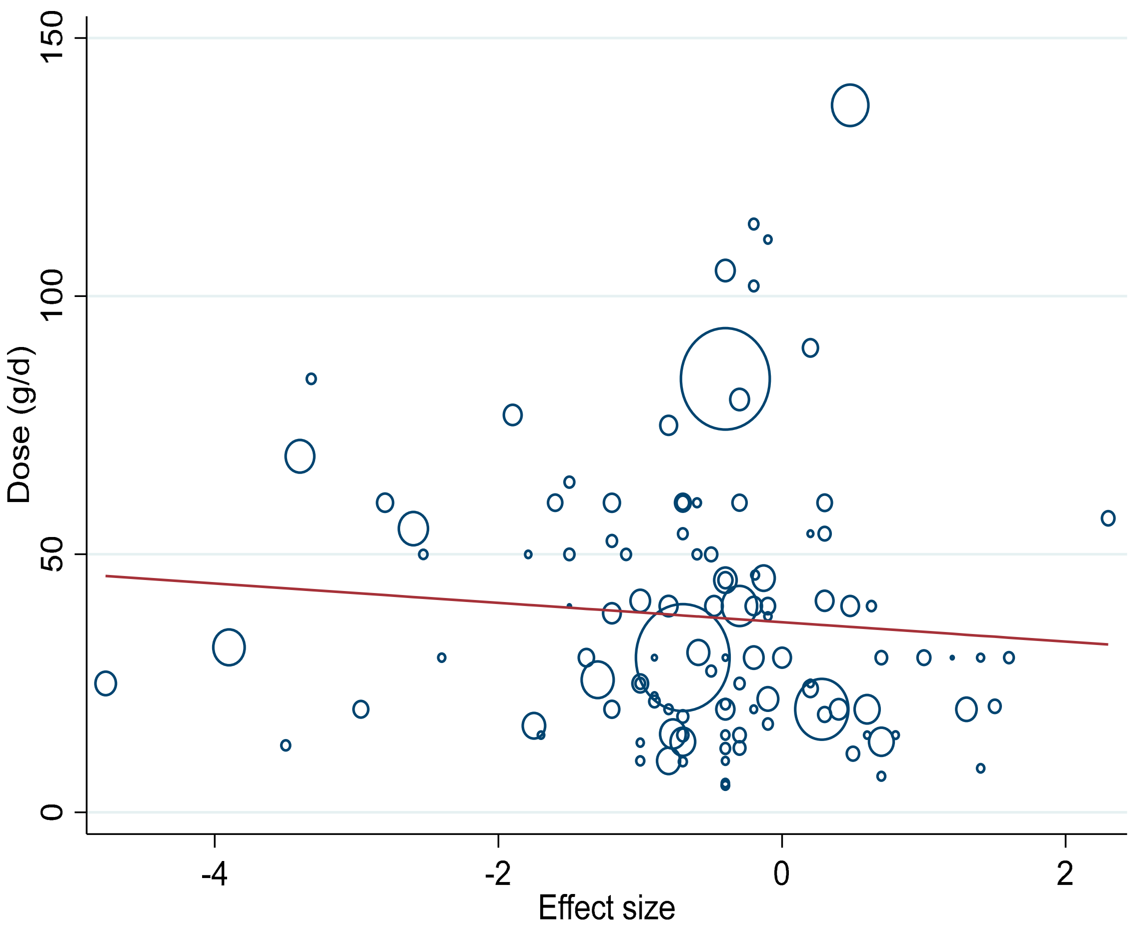
Dose (g/d)
150
100
50
0
-4
-2
0
2
Effect size

E) BFP

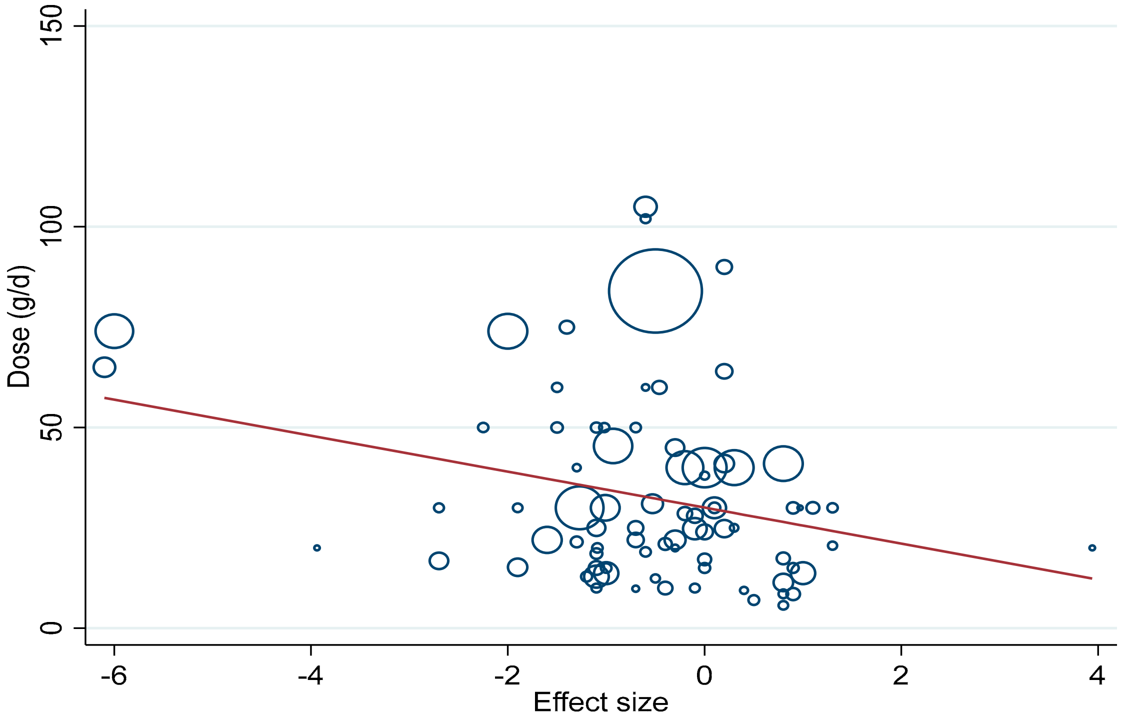

150
100
50
0
Dose (g/d)
-6
-4
-2
0
2
4
Effect size

F) FFM

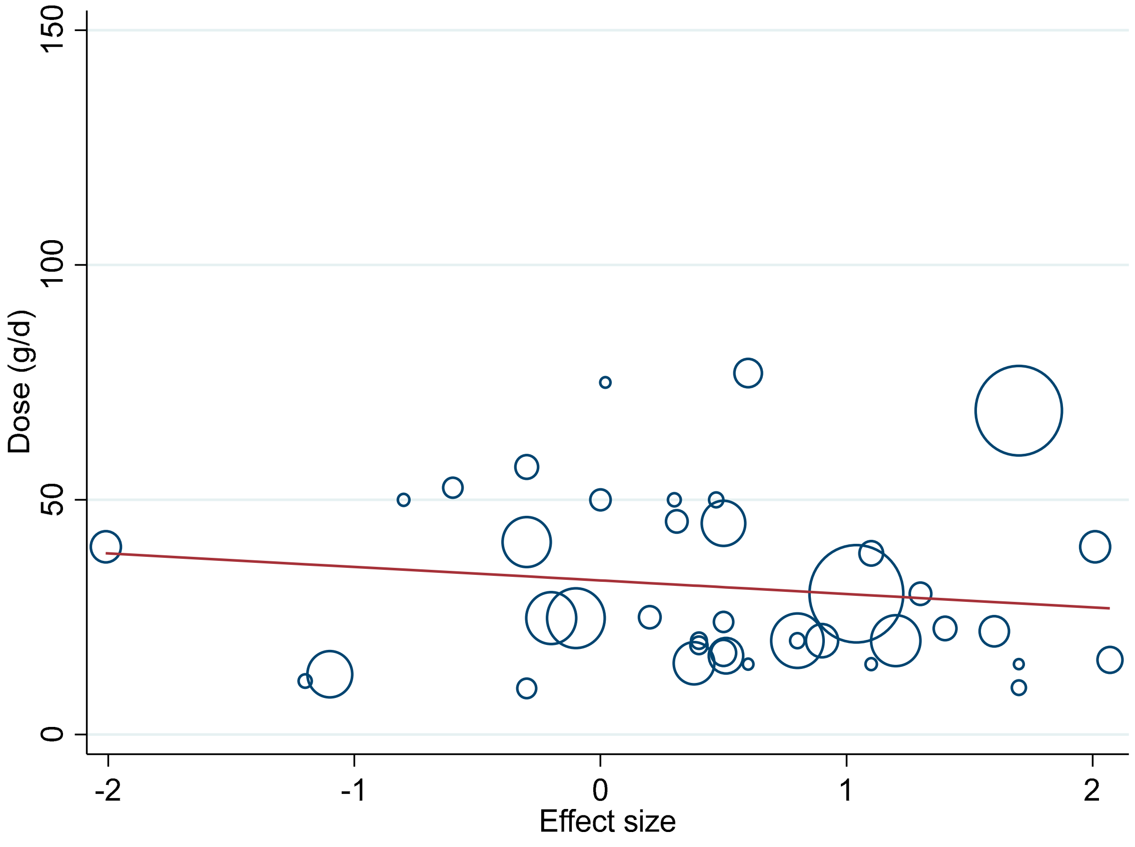

150
100
50
0
Dose (g/d)
-2
-1
0
1
2
Effect size

G) LBM

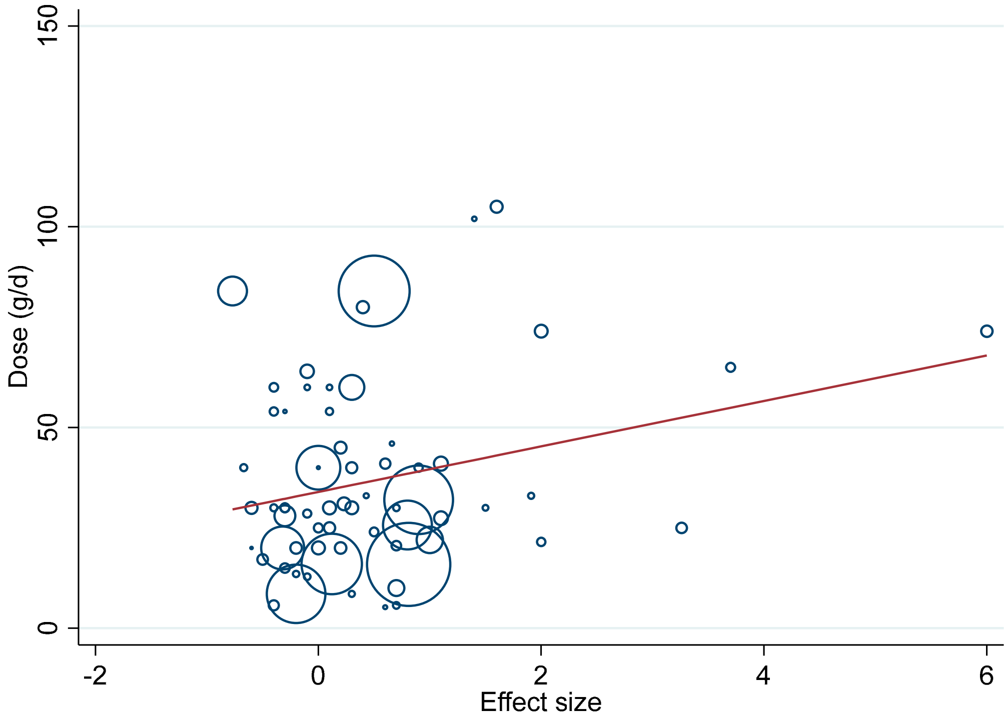

(H) MM

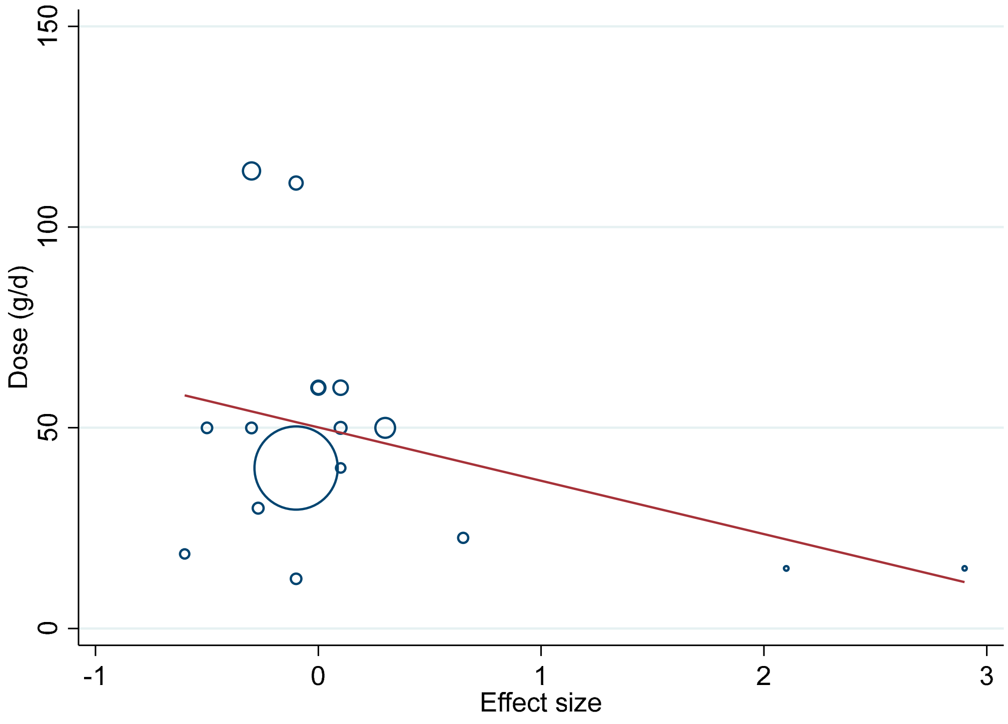

Figure S4. Linear dose-response association between dose (g/day) of milk protein supplementation and absolute mean differences in **(A)** BW (Kg), **(B)** BMI (kg/m²), **(C)** WC (cm), **(D)** FM (kg), **(E)** BFP (%), **(F)** FFM (Kg), **(G)** LBM (kg), and **(H)** MM (kg).

A) BW

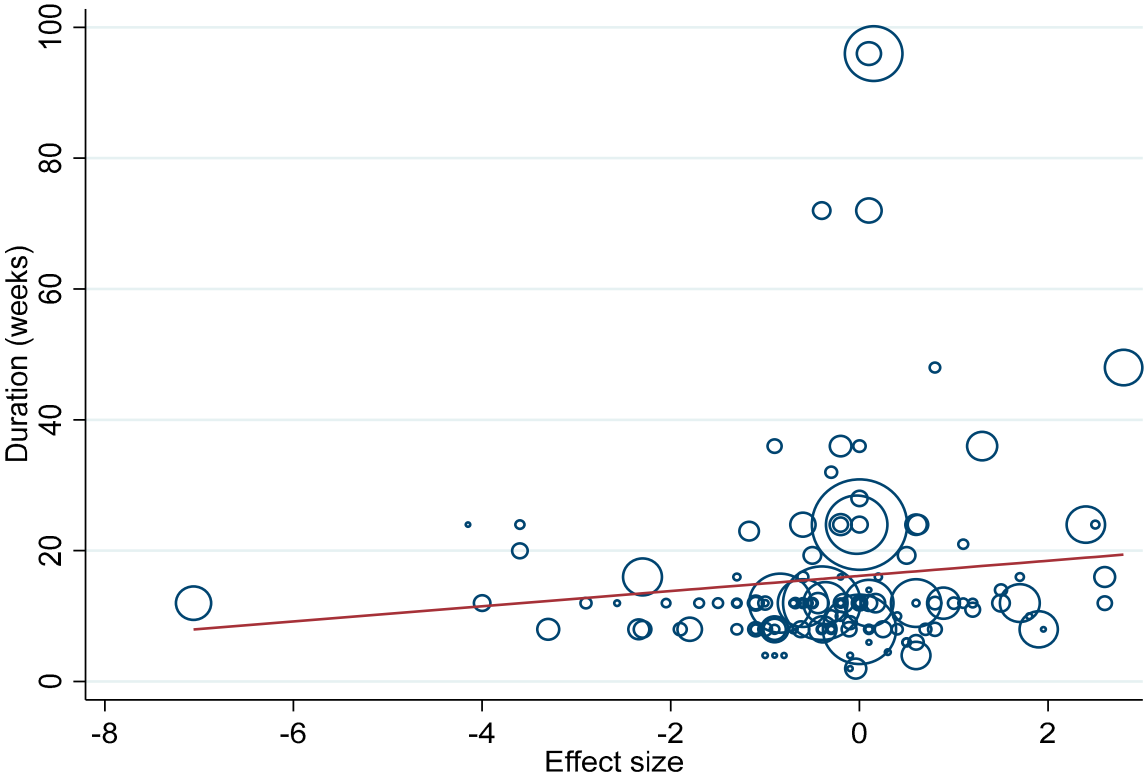
Duration (weeks)
0
20
40
60
80
100
-8
-6
-4
-2
0
2
Effect size

B) BMI

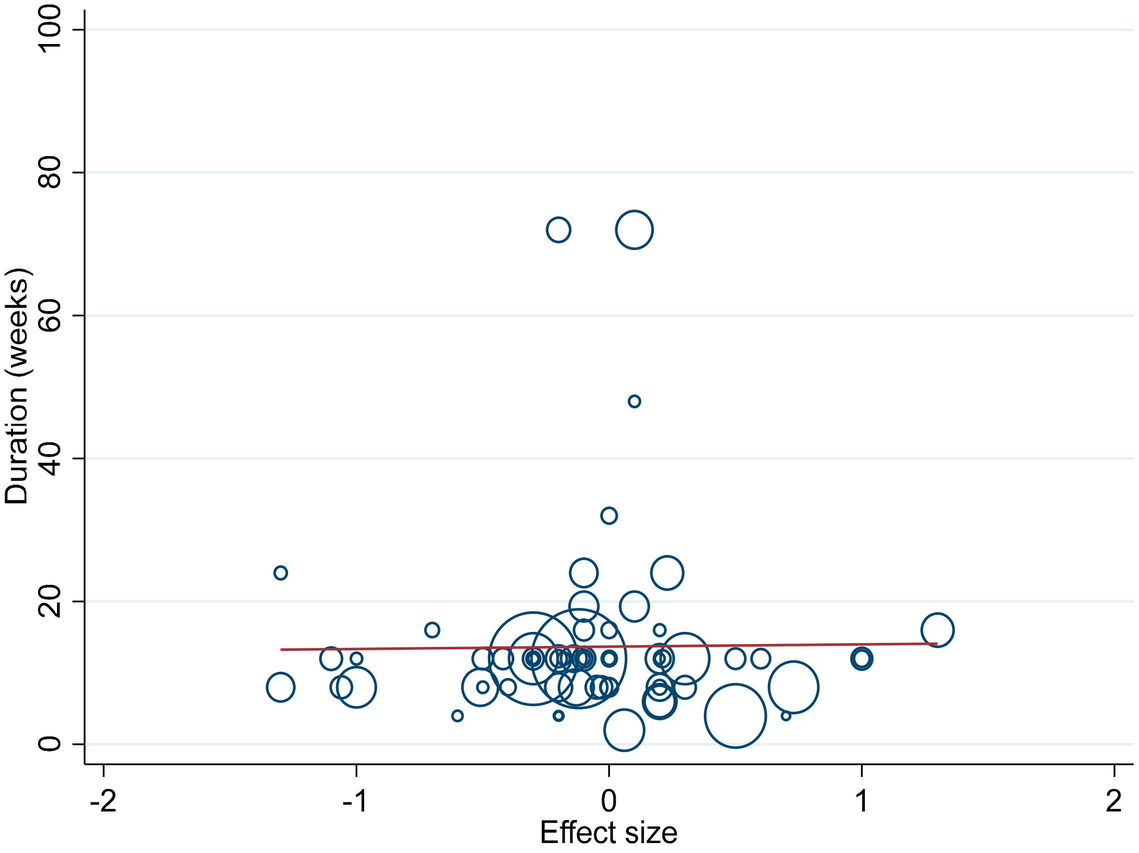
Duration (weeks)
0
20
40
60
80
100
-2
-1
0
1
2
Effect size

C) WC

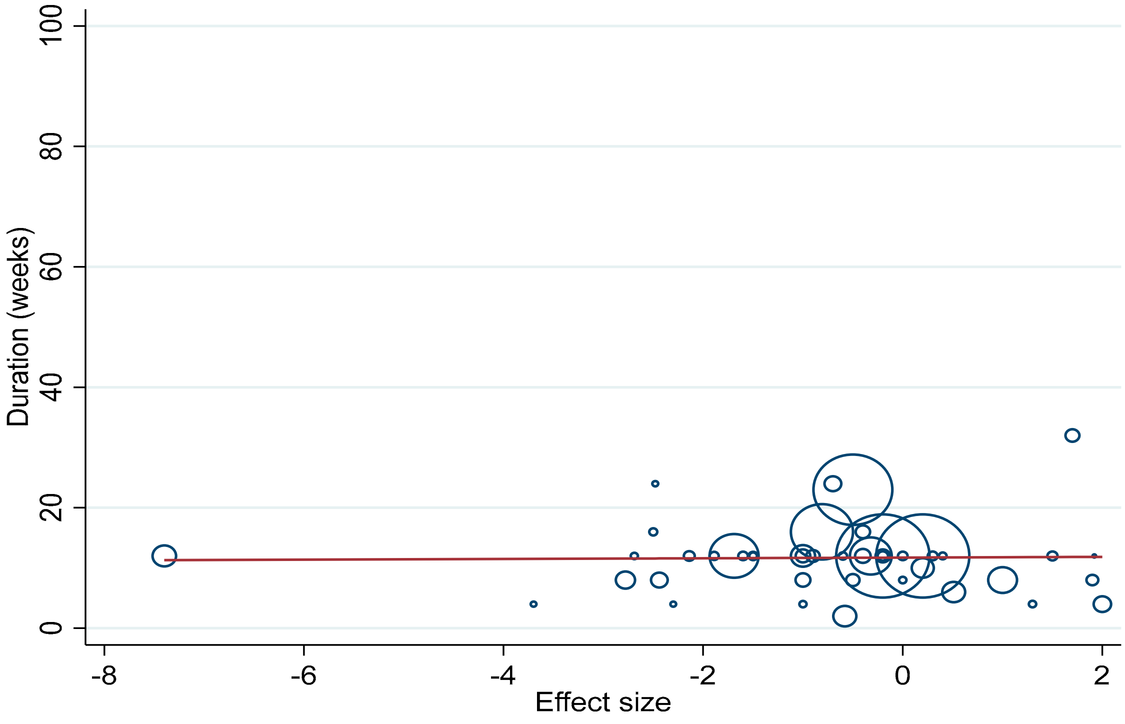
Duration (weeks)
0
20
40
60
80
100
-8
-6
-4
-2
0
2
Effect size

D) FM

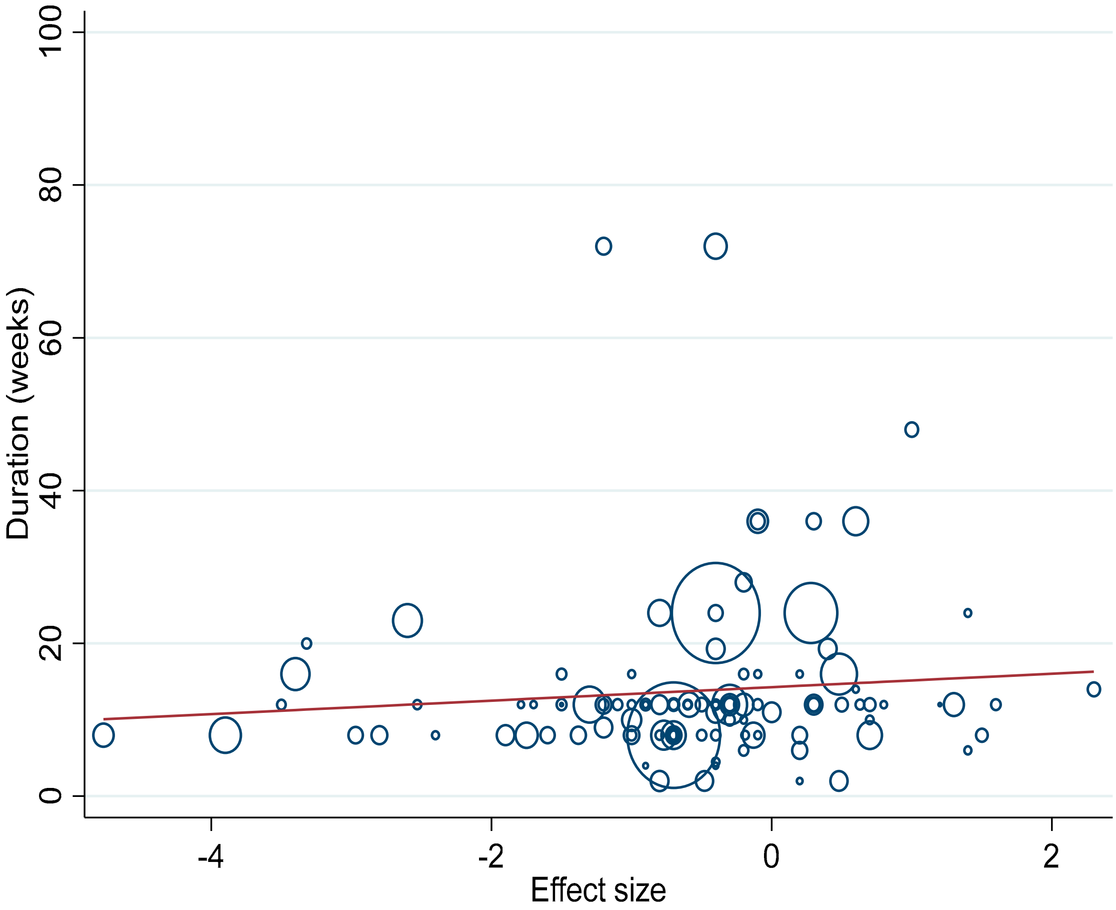
Duration (weeks)
0
20
40
60
80
100
-4
-2
0
2
Effect size

E) BFP

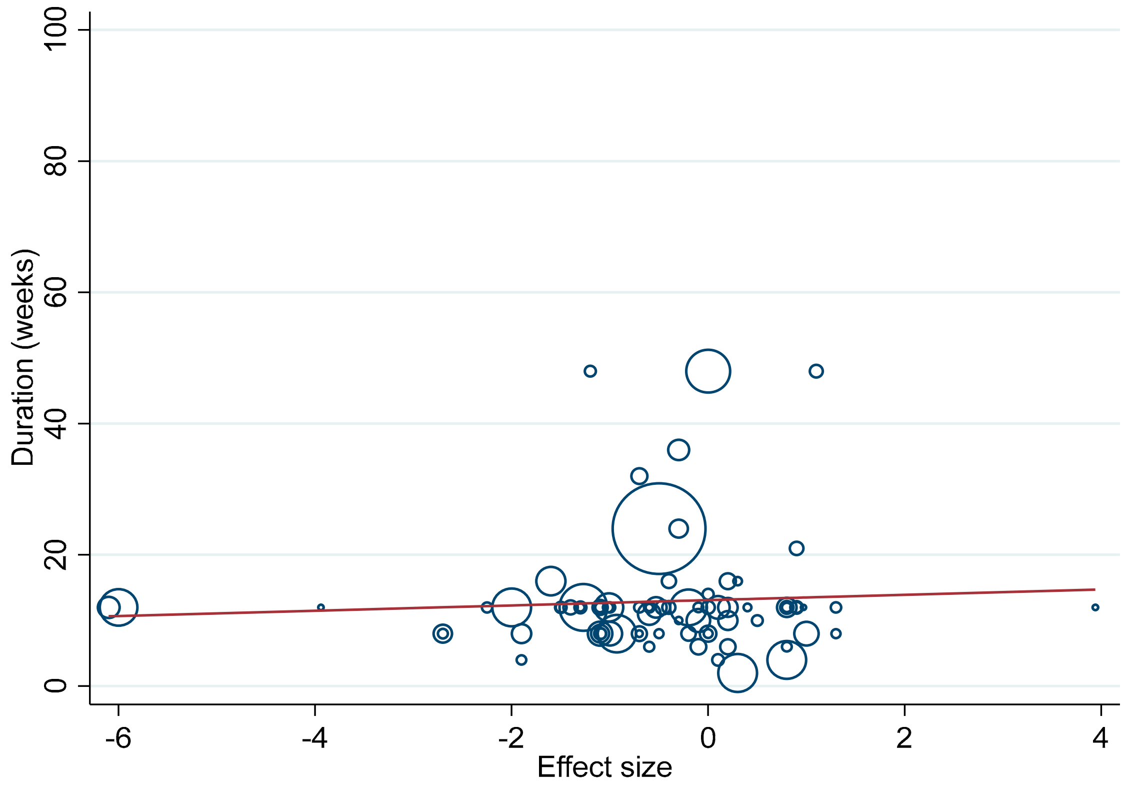

Duration (weeks)
0
20
40
60
80
100
-6
-4
-2
0
2
4
Effect size

F) FFM

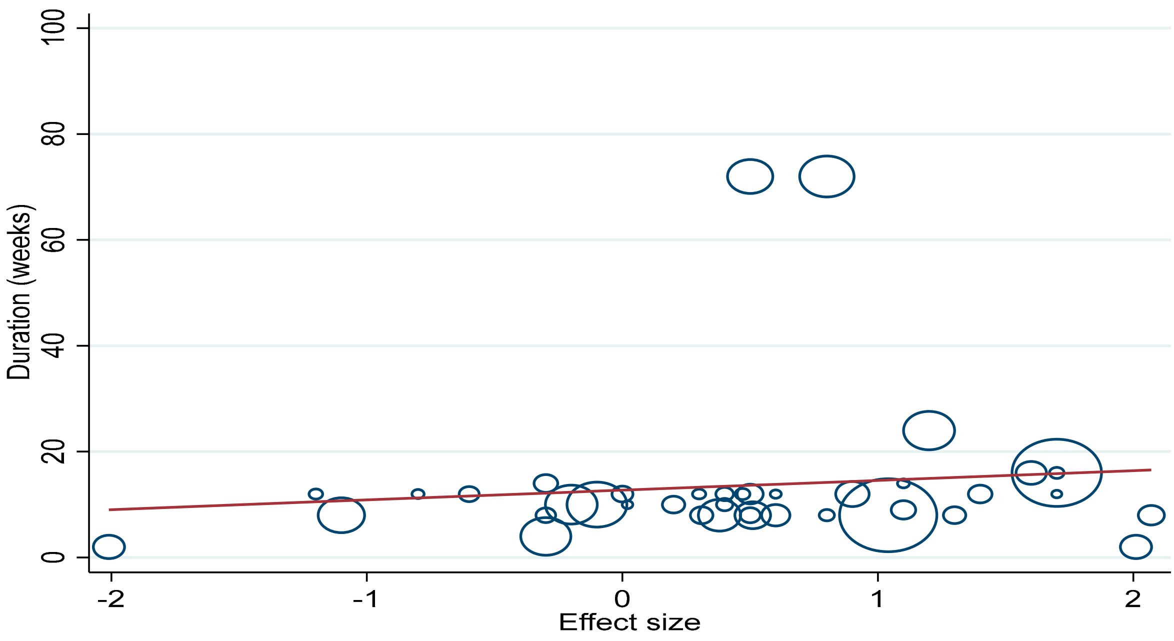

Duration (weeks)
0
20
40
60
80
100
-2
-1
0
1
2
Effect size

G) LBM

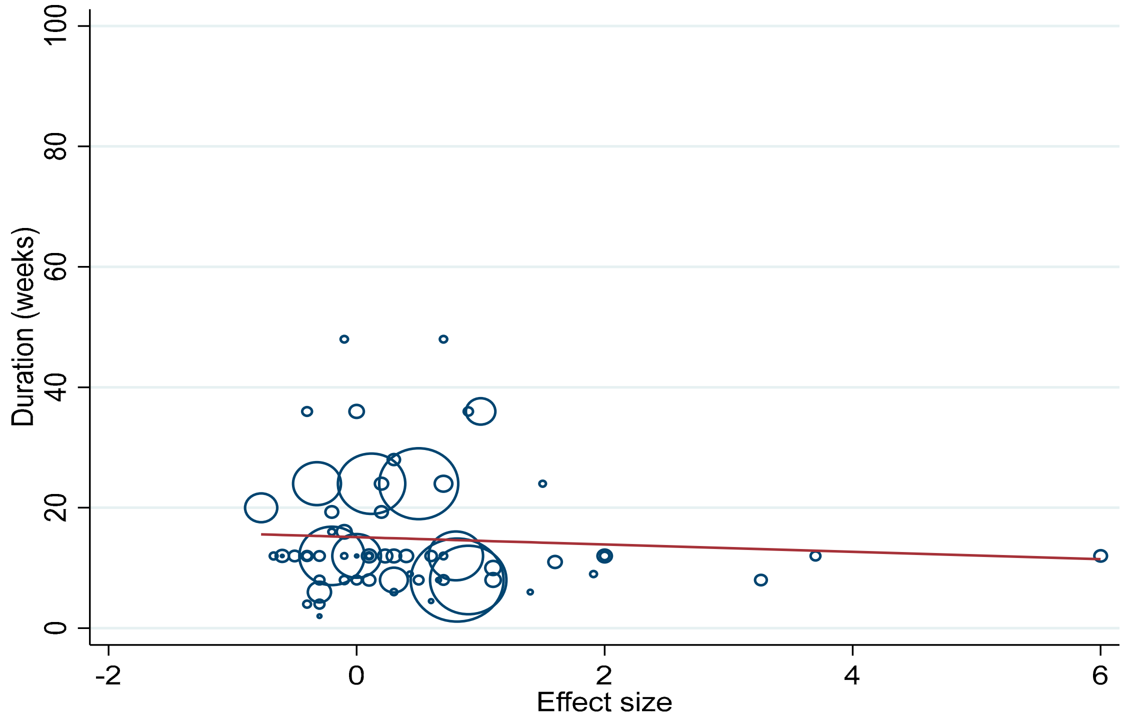

H) MM

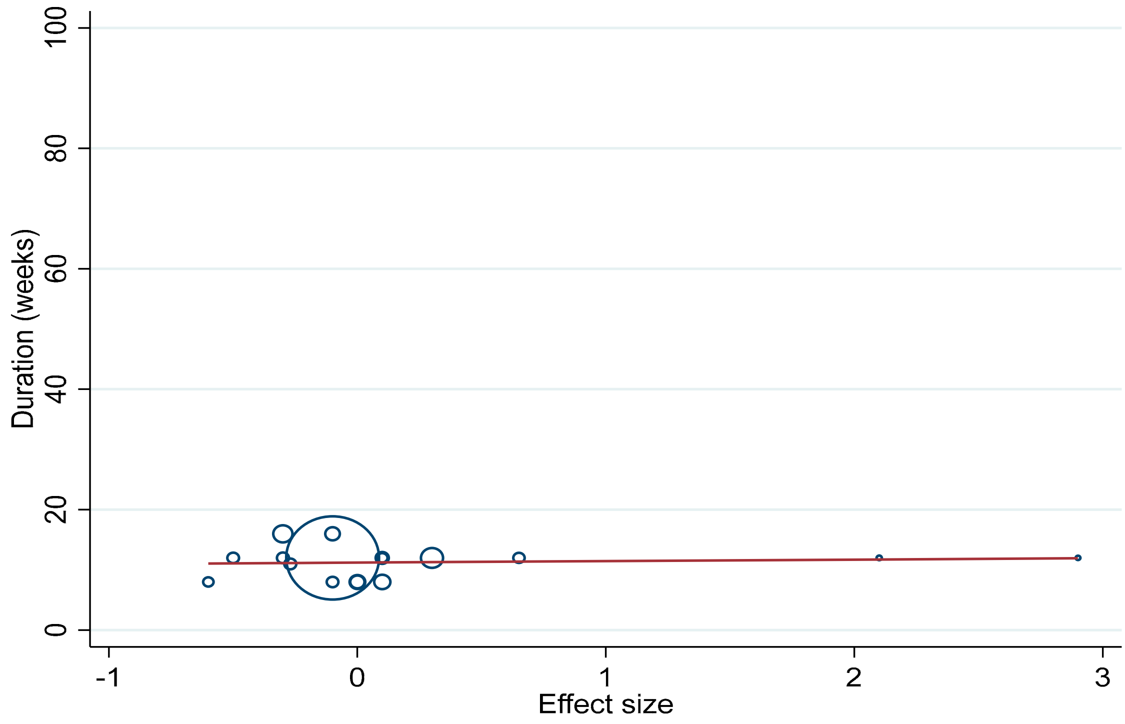

Figure S5. Linear dose-response association between the duration of supplementation with milk protein (weeks) and absolute mean differences in **(A)** BW (Kg), **(B)** BMI (kg/m^2), **(C)**WC (cm), **(D)** FM (kg), **(E)** BFP (%), **(F)** FFM(Kg), **(G)** LBM (kg), and **(H)** MM (kg).
